# Isomerization of Poly(ethylene glycol): A Strategy for the Evasion of Anti-PEG Antibody Recognition

**DOI:** 10.1021/jacs.5c02716

**Published:** 2025-06-13

**Authors:** Philip Dreier, Rebecca Matthes, Fabian Fuß, Julian Schmidt, Dominik Schulz, Gregor M. Linden, Ramona D. Barent, Sandra Schüttner, Barry W. Neun, Edward Cedrone, Marina A. Dobrovolskaia, Matthias Bros, Holger Frey

**Affiliations:** † Department of Chemistry, 9182 Johannes Gutenberg University Mainz, 55128 Mainz, Germany; ‡ Nanotechnology Characterization Laboratory, Cancer Research Technology Program, 437329 Frederick National Laboratory for Cancer Research Sponsored by the National Cancer Institute, 8560 Progress Dr., Frederick, Maryland 21701, United States; § University Medical Centre, Johannes Gutenberg University Mainz, 55101 Mainz, Germany

## Abstract

PEGylation, the conjugation of poly­(ethylene glycol) (PEG) to nanocarriers or protein-based active pharmaceutical ingredients (APIs), is a key strategy in nanomedicine to extend the circulation time of therapeutics in the bloodstream based on the stealth effect of PEG. However, the growing prevalence of anti-PEG antibodies in the population can lead to pronounced immune responses upon drug administration and accelerated blood clearance of PEGylated drugs, resulting in the loss of the stealth effect. We introduce the randomized PEG (rPEG) technology designed to strongly reduce the antigenicity of PEG while preserving its core benefits. This conceptually novel approach is based on an introduction of hydrophilic side chains along the PEG backbone. The synthesis is performed via anionic ring-opening copolymerization of ethylene oxide (EO) and glycidyl methyl ether (GME), resulting in constitutional isomers of PEG. By optimization of the reaction conditions, an ideally random distribution of the side chains in the polymer backbone could be achieved. Since previous studies show a relation between polymer chain regularity and immune system response, our approach specifically aims at introducing an irregular comonomer sequence via copolymerization, while translating the hydrophilicity and low toxicity of PEG to rPEG. Biocompatibility was evaluated using peripheral blood mononuclear cells (PBMC). Increasing the GME content in the copolymers did not decrease cell viability. Furthermore, all rPEG samples did not show complement activation in vitro at all tested concentrations. Enzyme-linked immunosorbent assays (ELISA) utilizing backbone- and end group-selective anti-PEG antibodies showed drastically reduced recognition and antibody binding for the constitutional isomers of PEG.

## Graphical abstract

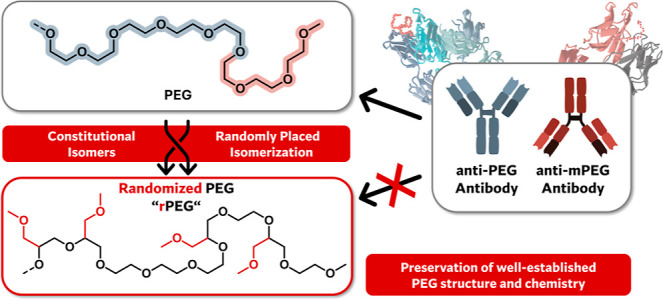

## Introduction

PEGylation, the conjugation of poly­(ethylene glycol) (PEG) to proteins, small molecules or lipids, is a crucial strategy for the delivery of peptide and protein drugs.^1,2^ It is frequently used in liposomal formulations^3,4^ and in PEGylated lipids as solubilizing and crucial stabilizing components of lipid nanoparticles, e.g. SARS-CoV-2 vaccines.^5,6^ Presently, over 40 PEGylated therapeutics are on the market or in clinical phase III, with market introduction pending.^7^ It is appropriate to state that PEGylation represents a key technology of current nanomedicine. The nonionic PEG provides a hydrophilic shield that protects conjugates from recognition by the patient’s immune system, commonly referred to as “stealth effect”. It effectively increases the size of the biomolecule, consequently reducing clearance from the bloodstream.^8^ The investigation of PEG-coated nanocarriers additionally revealed the impact of PEG on the composition of the protein corona formed at the particles, which is important to prevent nonspecific cellular uptake.^9^ However, whereas it was initially believed that PEG is immunologically inert, it has become evident in recent decades that an increasing number of individuals have developed anti-PEG antibodies (APAs), extensively boosted by PEG lipid-containing SARS-CoV-2 lipid nanoparticles (LNPs) used for mRNA vaccinations.^10–12^ The induction of APAs in humans may not only result from PEGylated therapeutics but could also arise from exposure to PEG present in food, cosmetics and other common sources. The potential antigenicity of PEG has been confirmed by the existence of APAs in healthy individuals who have never received PEGylated therapeutics systemically.^13,14^ A recent study revealed that 83% of individuals in a typical western population are positive for either anti-PEG IgG or IgM.^15^

The presence of APAs results in the recognition and accelerated blood clearance of PEGylated therapeutics, premature drug release from PEGylated nanocarriers, and hypersensitivity reactions, including complement activation-related pseudoallergy (CARPA) and, in severe cases, anaphylactic shock.^16^ A phase III clinical study regarding anticoagulation factor IXa RNA PEG-conjugated aptamer had to be interrupted since anaphylactic reactions in 0.6% of the patients were observed, most likely caused by the pre-existence of APAs in the bloodstream.^17^ Recent clinical works regarding Pegaspargase have shown that the presence of APAs permits the prediction of allergic reactions and failure of rechallenge, emphasizing the clinical relevance of APA-Fabs for the success of treating leukemia in this case.^18^ Based on these concerns, the search for PEG replacement structures has intensified in recent years. Several alternatives based on polymer classes other than polyethers, i.e. polysarcosine, polyoxazolines, and polymethacrylates have been discussed.^7,8,19^ These alternatives feature well-studied biocompatibility.^20,21^ However, it is important to note that antigenicity was also observed for some homopolymers of polymer classes other than PEG^22,23^ due to the flexibility of the adaptive immune system.

In contrast to the aforementioned approaches, the presented study targets the “PEG alternative dilemma” via a fundamentally different “PEG isomerization” approach. Our strategy aims to preserve the wide spectrum of beneficial properties of PEG, while diminishing its immunological drawbacks by the introduction of statistical heterogenicity, retaining precise control over the chain length. Additionally, we aim at a PEG-based alternative that could be seamlessly integrated into existing PEG manufacturing and PEGylation processes.

Recently, crystal-structures of two types of APAs that bind to the PEG backbone^24^ or the methoxy end group of mPEG^25^ were determined for the first time. In both cases, an open ring-like substructure of the APA Fab paratope recognizes ethylene glycol (EO) units of PEG via multiple van der Waals and polar interactions. While the backbone-selective APA binds 16 EO units, seven units and the methoxy residue at the chain end are recognized by the investigated end group-selective APA. Additionally, a third type of APA has been reported which interacts with a core PEG fragment in combination with a PEG satellite fragment.^26^ Inspired by these observations, we hypothesized that an interruption of the periodic and linear PEG structure by randomly distributed branching points should significantly alter the interaction of the APA with the PEG epitope. This random disruption of the regular PEG structure may be viewed as an introduction of ‘synthetic point mutations’ along the polyether backbone. In this context, we chose methoxy methylene side chains as branching points to preserve the carbon-hydrogen-oxygen ratio and properties of PEG while altering the underlying architecture of the polyether (Figure 1A).

**Figure 1:**
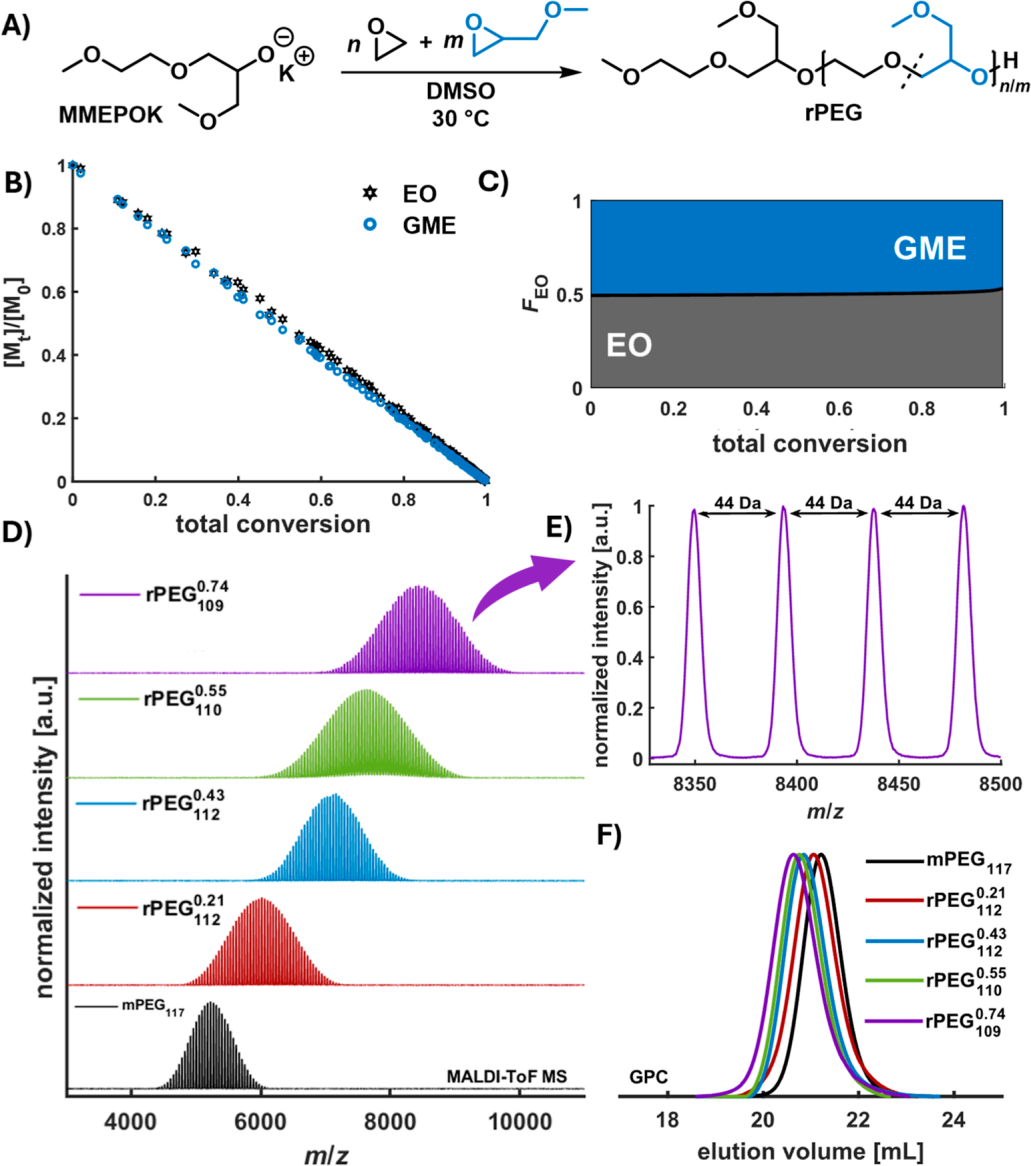
Synthesis and characterization of rPEGs. (A) Applied synthesis strategy for rPEG by copolymerization; (B) comonomer consumption ([*M*_t_]/[*M*_0_]) vs total conversion obtained from in situ ^1^H NMR kinetics in DMSO-*d*_6_; (C) molar-based composition diagram of rPEG (50 mol % GME) derived from the in situ ^1^H NMR kinetics data in DMSO-*d*_6_; (D) stacked MALDI ToF mass spectra of investigated rPEGs; (E) zoom-in of rPEG_109_^0.74^ MALDI ToF MS; (F) stacked GPC traces of investigated rPEGs.

## Results and Discussion

The repeating unit can be introduced via the epoxide monomer glycidyl methyl ether (GME). As GME is a formal dimer of EO (C_2*n*_H_4*n*_O_*n*_; *n* = 1 (EO), 2 (GME)), all copolymers of EO and GME represent constitutional isomers of PEG, independent of their monomer composition. We assumed that these randomized PEG (rPEG) structures hold potential as a highly suitable nonantigenic alternative to PEG, albeit preserving the polyether backbone and the beneficial physicochemical properties of PEG. The random incorporation of EO and GME units into the polymer backbone is an essential parameter to decrease the occurrence of EO-rich epitope segments in the polyether chains. In principle, the incorporation of GME repeating units can be achieved via copolymerization of EO and GME. Noteworthy, anionic homo- and copolymerization of GME has rarely been investigated to date due to synthetic challenges and impurities (Scheme S2), resulting in ill-defined polymers that are not suitable for pharmaceutical applications. PGME homopolymers were either obtained as oligomers^27^ or with high dispersity.^28^ EO-GME copolymer structures are accessible using an activated monomer mechanism^28^ albeit resulting in ill-defined materials with poor end group fidelity and nontolerable traces of the aluminum catalyst in the products. It is important to note that for a polymer to be regarded as a viable alternative to PEG, it must meet the standard specifications of pharmaceutical-grade PEG. Specifically, it must possess high purity and a dispersity (*M*_w_/*M*_n_) lower than 1.10. High end group fidelity (>99%) is critical for the subsequent conjugation of the polymers to proteins, nanocarriers or surfaces.

Via utilization of analytically pure GME (>99%) (Scheme S1, Figures S1, S4 and S5) and anionic ring-opening (co)­polymerization (AROP) of EO and GME (Figure 1A), the first successful synthesis of well-defined rPEGs was accomplished, circumventing the abovementioned synthetic challenges (Scheme S2). AROP was selected as polymerization method of choice, as it represents the standard polymerization technique for pharmaceutical grade PEG, characterized by the absence of toxic side products or catalysts, high end-group fidelity and the ability to precisely control molar masses due to quantitative conversion of the epoxide monomers.^29^ The method is also utilized in the industrially established synthesis of pharmaceutical-grade PEG. Therefore, it is the key for the potential synthesis of a polyether-based PEG alternative in existing PEG production facilities.

In the AROP of epoxides, the choice of solvent is a crucial reaction parameter because it has a direct influence on the copolymerization kinetics and the resulting microstructure of the copolymer.^30^ Therefore, in a first optimization step, we elucidated the incorporation of EO and GME into the polymer backbone by following the mean composition at all chain positions during the copolymerization. This was achieved by performing and evaluating in situ ^1^H NMR kinetics measurements in various nonprotic solvents suitable for AROP of monosubstituted epoxides (Figures S8–S39 and Tables S1 and S2). In the case of DMSO-*d*_6_, an almost linear decrease of comonomer concentration with progressing conversion (Figure 1B) for both comonomers is observed. This demonstrates that the incorporation of EO and GME units occurs in an ideally random manner (*r*_EO_ ≈ *r*_GME_ ≈ 1) (Figure 1C) independent of temperature and degree of deprotonation (Figures S15–S18, S24–S26). In the case of less polar solvents, a slightly preferred incorporation of GME over EO was observed, resulting in soft gradient microstructures (Figures S19–S23, S27–S31). As a random distribution of GME along the polymer backbone is preferred to statistically minimize the occurrence of EO-rich segments, we chose DMSO as a suitable solvent for the optimization of the rPEG synthesis on multigram scale.

To ensure systematic comparability of the side groups’ influence on the physicochemical properties and their behavior in the bioassays, copolymers in analogy to 5000 g mol^–1^ mPEG were synthesized. This translates to a constant degree of polymerization of 114, while varying the GME content, leading to higher molar masses that depend on the introduced GME content. In this context, we designed and utilized the potassium salt of 1-methoxy-3-(2-methoxyethoxy)­propan-2-ol (MMEPOK) as initiator (Figure 1A) for the synthesis of the rPEG samples to lock a ‘synthetic point mutation’ at the second repeating unit of each polymer chain (Supporting Information). The design of the initiator structure was derived from preliminary enzyme-linked immunosorbent assay (ELISA) experiments of PGME samples with differing initiator molecules (Figure S40). We further designed and conducted an additional purification protocol via analytical (Figures S41 and S42) and semipreparative high-performance liquid chromatography (HPLC) (Supporting Information) to obtain rPEG samples in high purity which is an essential requirement for potential pharmaceutical applications (Figure S43, Table S5). Additionally, the purification protocol was applied using a commercial mPEG sample to allow for comparison between mPEG and rPEG in the following studies. Hence, rPEGs with varying molar compositions of 21–74 mol % GME and molar masses of 6.0–8.4 kg mol^–1^ were synthesized and purified (Tables S3 and S4). All isomeric rPEGs and mPEG were analyzed by ^1^H NMR spectroscopy (Figure S44) and MALDI ToF MS. The latter reveals one distinct distribution which is assigned to the potassium-ionized and methoxy-initiated species ([rPEG + K]^+^) carrying an alcohol group at the ω-chain end exclusively (Figures 1D,E, S45 and S46). In addition, primary and secondary hydroxyl end group functionalities were quantified with ^31^P NMR spectroscopy to verify the ideally random copolymerization (Figure S54). As an example, rPEG_110_^0.55^ was investigated and the expected content of secondary hydroxyl end groups (54%) was confirmed.^31^ This supports the purity and an excellent end group fidelity of the synthesized rPEGs. It is important to note that due to the isomeric character of the rPEGs, only a single distribution with intervals of 44 *m*/*z* is observed, despite the copolymer structure. Additionally, all synthesized rPEGs show monomodal distributions and dispersity <1.10 (GPC) independent of their molar comonomer composition (Figures 1F, S47). Therefore, from a synthetic point of view, the requirements of rPEGs as a PEG alternative are fully met.

The application of PEG alternatives in polymer–protein conjugates and in lipid-nucleic acid formulations under physiological conditions necessitate sufficient solubility of the polymer in aqueous solutions significantly above body temperature. Since the formation of a hydration shell is crucial for the stealth effect and shielding of the active pharmaceutical ingredient (API) or nanoparticle, a collapse of the PEGylation alternative in aqueous solutions must be prevented.^32,33^

Therefore, we investigated the thermoresponsive behavior of the synthesized rPEG samples in PBS buffer via turbidimetry. In the measurements, the cloud point temperatures (*T*_cp_), marking phase separation of polymers and the solvent of the rPEGs with varying amounts of GME were determined at a concentration of 2 mg mL^–1^ (Figure 2A, Table S6). The investigation shows that no cloud point is observed up to a molar GME content of 43% in the rPEG samples. The observed *T*_cp_ at a transmittance of 50% for rPEG_110_^0.55^ and rPEG_109_^0.74^ at 86 and 75 °C, respectively, demonstrate that even for high content of GME no coil-globule transition is to be expected at physiological temperatures. Additionally, cloud point temperatures for rPEG_109_^0.74^ at varying polymer concentrations (0.5–10.0 mg mL^–1^) were investigated showing merely a slight decrease of the *T*_cp_ to 71 °C (10.0 mg mL^–1^) with increasing polymer concentration (Figure 2B, Table S6). For comparison, the PGME homopolymer exhibits a *T*_cp_ of 66 °C (Figure S61) in PBS buffer at a concentration of 5 mg mL^–1^. These findings underline the critical role of the EO/GME comonomer combination in modulating the overall hydrophilicity of the rPEGs.

**Figure 2:**
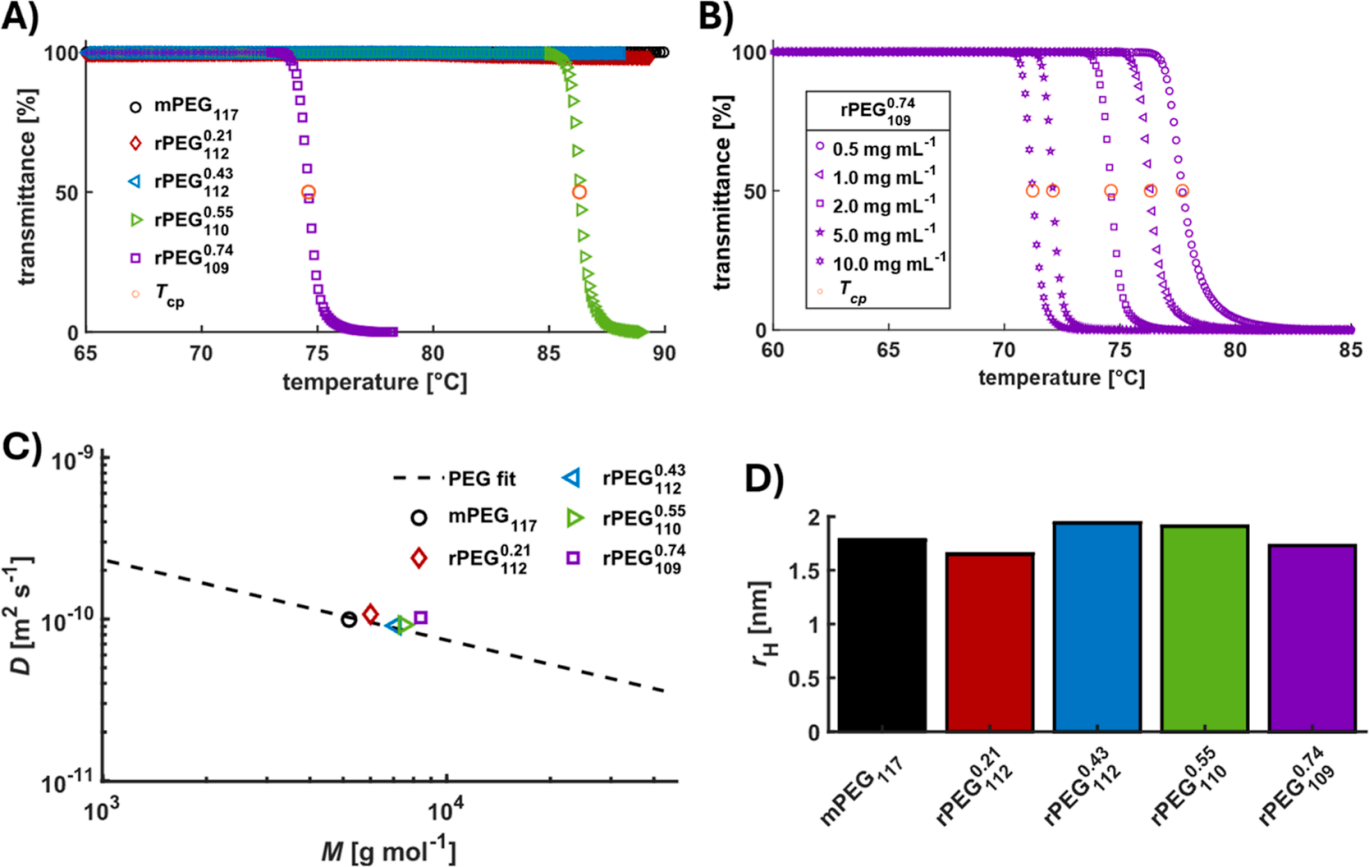
Physicochemical properties of rPEGs. (A) Stacked turbidimetry plots (heating curves) (2.0 mg mL^–1^) of mPEG_117_ and rPEG samples in PBS buffer; (B) stacked turbidimetry plots (heating curves) of rPEG_109_^0.74^ at different concentrations in PBS buffer; (C) diffusion coefficients in dependence of the molar mass for mPEG and rPEG samples in D_2_O obtained via DOSY NMR; dashed line shows PEG fit determined from different well-defined PEG samples; (D) comparison of hydrodynamic radii of mPEG and rPEG samples based on the diffusion coefficients obtained from DOSY experiments.

Pursuing, we utilized diffusion-ordered NMR spectroscopy (DOSY NMR) in D_2_O (Figures S48–S52) to evaluate the influence of the methoxy methylene side chains on the properties of rPEGs in solution. The obtained data (Table S7) was referenced to a PEG fit (Figure S53) to elucidate differences of the rPEG samples to conventional PEG. In this context, the rPEG samples exhibit slight deviations from the expected PEG fit values, whereas the mPEG_117_ sample aligns closely with the fit (Figure 2C). This confirms that the methoxy end group of the investigated samples has no significant influence on the diffusion of the polymers in the investigated molar mass range. Calculation of the hydrodynamic radii (*r*_H_) from the diffusion data (Supporting Information) reveals unexpected behavior. Despite the significant increase in molar mass with higher content of GME, the *r*_H_ of the rPEG samples remains comparable to that of the mPEG reference (Figure 2D, Table S7). In comparison, a PEG sample with the same molar mass as rPEG_109_^0.74^ (*r*_H_ = 1.73 nm) possesses a *r*_H_ of ≈2.23 nm based on the determined PEG fit (Figure S53). From these observations, we conclude that the primary contribution to the hydrodynamic radius of the rPEG polymer coil derives from the hydration of the backbone, while the methoxy methylene side groups play a rather minor role.

In summary, the study of the thermoresponsive properties and hydrodynamic radii of rPEGs in buffer solution and D_2_O, respectively, show similar results as for PEG, hinting at comparable shielding capability for drug- or protein-conjugates and nanoparticles. Naturally, the preservation of the stealth effect in a biological system can only be determined by in vivo studies, which are currently in progress and will be published in due course.

Further, we extensively assessed the in vitro biocompatibility of rPEG. Therefore, we analyzed cell viability, immunostimulatory effects as well as the hematological profile. It was shown in earlier studies that the PGME homopolymer and other glycidyl ether-based polyethers show high biocompatibility.^27,34^ To establish a PEG-like safety profile for rPEG, two independent series were tested by the University Medical Centre of the Johannes Gutenberg University Mainz (Germany) and the National Cancer Institute’s Nanotechnology Characterization Laboratory (NCL) (United States). In the first series peripheral blood mononuclear cells (PBMCs) of four healthy German donors were exposed to rPEGs and mPEG_117_ with three concentrations (0.1–5.0 mg mL^–1^) for 16 h to evaluate the cell viability and immunostimulation (Figures 3A, B, and S67–S69).

**Figure 3:**
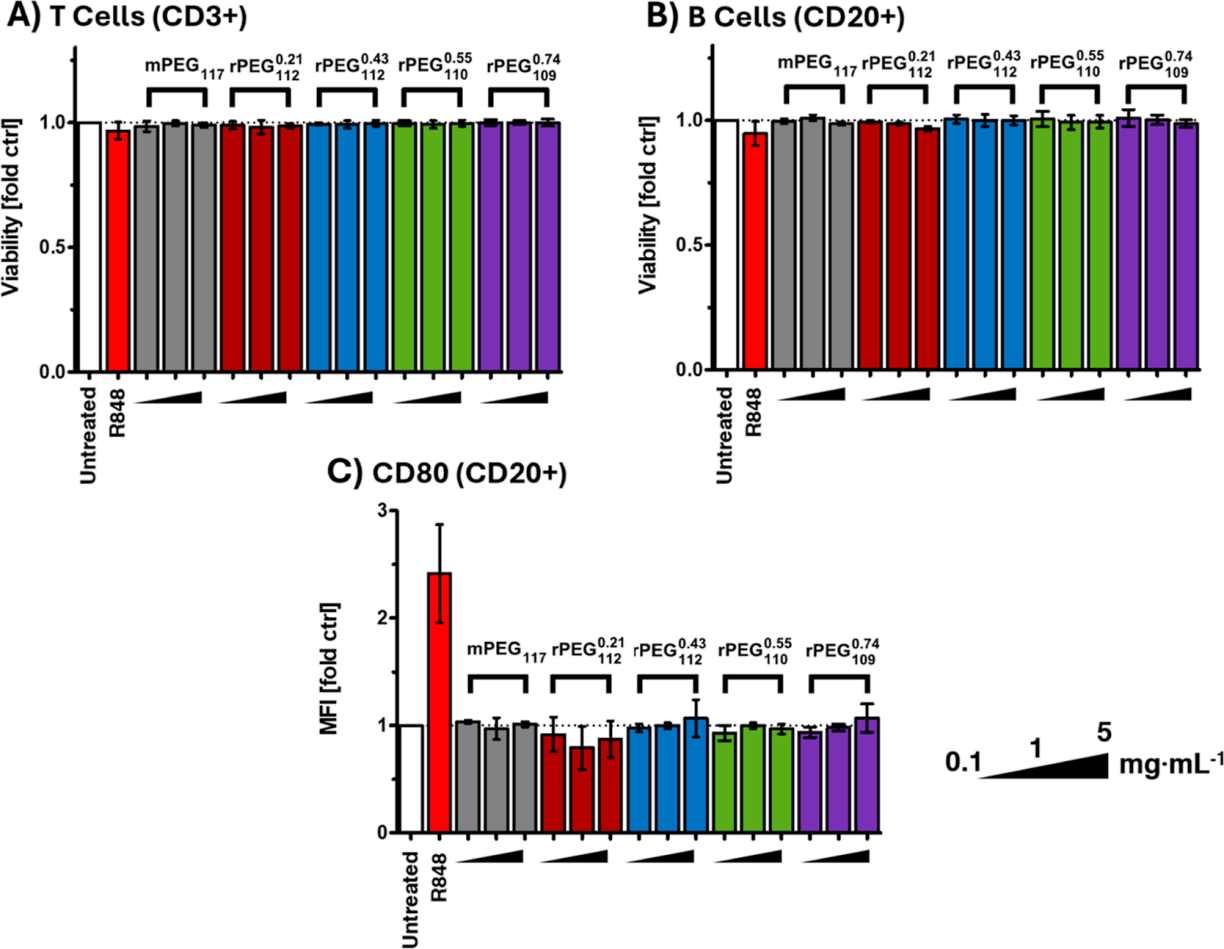
PBMC cell viability & B cell activation after exposure of mPEG_117_ and rPEGs. (A,B) Cell viability of human leukocytes; (C) CD80 expression on B Cells (CD20 positive) as a biomarker for immunostimulatory effects; determined via flow cytometry at three different concentrations (0.1–5.0 mg mL^–1^).

No influence on the viability of B cells, T cells, neutral killer cells (NK) as well as monocytes was observed for rPEGs, and no difference was noted between mPEG and rPEG samples. At the highest concentration of 5 mg mL^–1^, which is equivalent to an extremely high in vivo human dose of 400 mg kg^–1^, some rPEG samples showed a slight effect on dendritic cells (DC) and polymorphonuclear leukocytes. Nevertheless, their viability remains above 75% (fold ctrl) (Figures 3A,B, and S67). It can be assumed that this effect can be attributed to the high polymer concentration above any clinically relevant dose. A reduction of cell viability with increasing GME content is not observed. Additionally, the expression of surface activation markers, such as CD80 and CD86, remained at basal levels (Figures 3C, S68), providing the first indication that rPEG does not induce immunostimulation.

In the second series, slightly different samples were used for immunostimulatory and hematological testing, as these experiments were independently conducted by the NCL. Specifically, rPEG_120_^0.45^ was used instead of rPEG_112_^0.43^, which also complied with the acceptance criteria as the other rPEG samples. rPEG_110_^0.55^ was excluded due to its compositional similarity to rPEG_120_^0.45^. The results of the in vitro studies suggest that the rPEG technology does not trigger innate immune responses under the tested conditions. When human PBMC from three healthy donors were exposed to various concentrations of rPEG, induction of cytokines responsible for the pyrogenic response and fever-like reactions (TNF, IL-1β, and IL-6), produced by activated dendritic cells (IFNα, and IFNλ), and by activated T-cells (IL-2, IFNγ, and IP-10) was not observed at all tested concentrations (Figure 4). Therefore, it can be established that rPEG does not induce pyrogenic biomarkers, does not activate primary T-cells, resting B cells, and innate immune responses in agreement with the results from the series with the German donor population (Figures S67–69).

**Figure 4:**
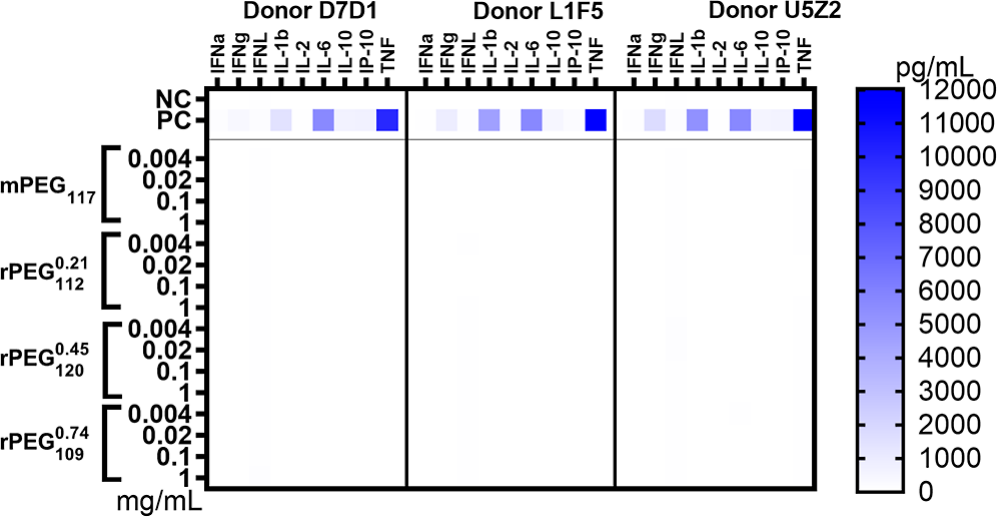
Cytokine biomarkers for activated myeloid and lymphoid cells in PBMC cultures exposed to mPEG117, rPEG_112_^0.21^, rPEG_120_^0.45^ and rPEG_109_^0.74^. PBS, and a combination of PHA-M, LPS, and ODN2216 were used as negative control (NC) and positive control (PC), respectively. The same data with masked positive control to highlight insignificant background cytokine levels is shown in the Supporting Information (Figure S75).

To investigate rPEG compatibility with human blood, we followed the ISO standard 10993-4 for medical devices, which is also generally adapted for hemocompatibility assessment of nanomedicines.^35,36^ None of the four samples (mPEG and rPEGs) with low (21 mol %), medium (45 mol %) and high GME content (74 mol %) lead to complement activation in vitro at all tested concentrations (Figure 5), and no difference was noted between the mPEG and rPEG samples. Similar results were obtained in plasma coagulation and platelet aggregation assays: all test samples did not induce platelet aggregation, did not affect collagen-induced platelet aggregation, and did not alter human plasma coagulation time in prothrombin, activated partial thromboplastin, and thrombin time assays (Figure 5). The data indicate rPEG would not induce complement activation when present in the blood at these concentrations. The highest tested concentration, 1 mg mL^–1^, is equivalent to a rPEG in vivo human dose of 80 mg kg^–1^. This suggests that the risk of complement activation-related pseudoallergy (CARPA) at rPEG doses up to 80 mg kg^–1^ is marginal. In contrast, Cremophor-EL tested at a concentration equivalent to that of the clinical dose of Taxol (Cremophor-formulated paclitaxel) resulted in complement activation consistent with the current knowledge of CARPA in sensitive Taxol recipients. Likewise, the risk of thrombogenicity due to platelet activation, and hemorrhage due to the inhibition of platelet or coagulation factor functions is also low for rPEG at all tested concentrations equivalent to doses up to 80 mg kg^–1^. To conclude, all investigated rPEGs are noncytotoxic, do not activate innate immune responses, are compatible with blood, and are not immunostimulatory at clinically relevant concentrations. Thus, rPEG appears to be suitable for utilization in in vivo experiments and additional preclinical studies.

**Figure 5:**
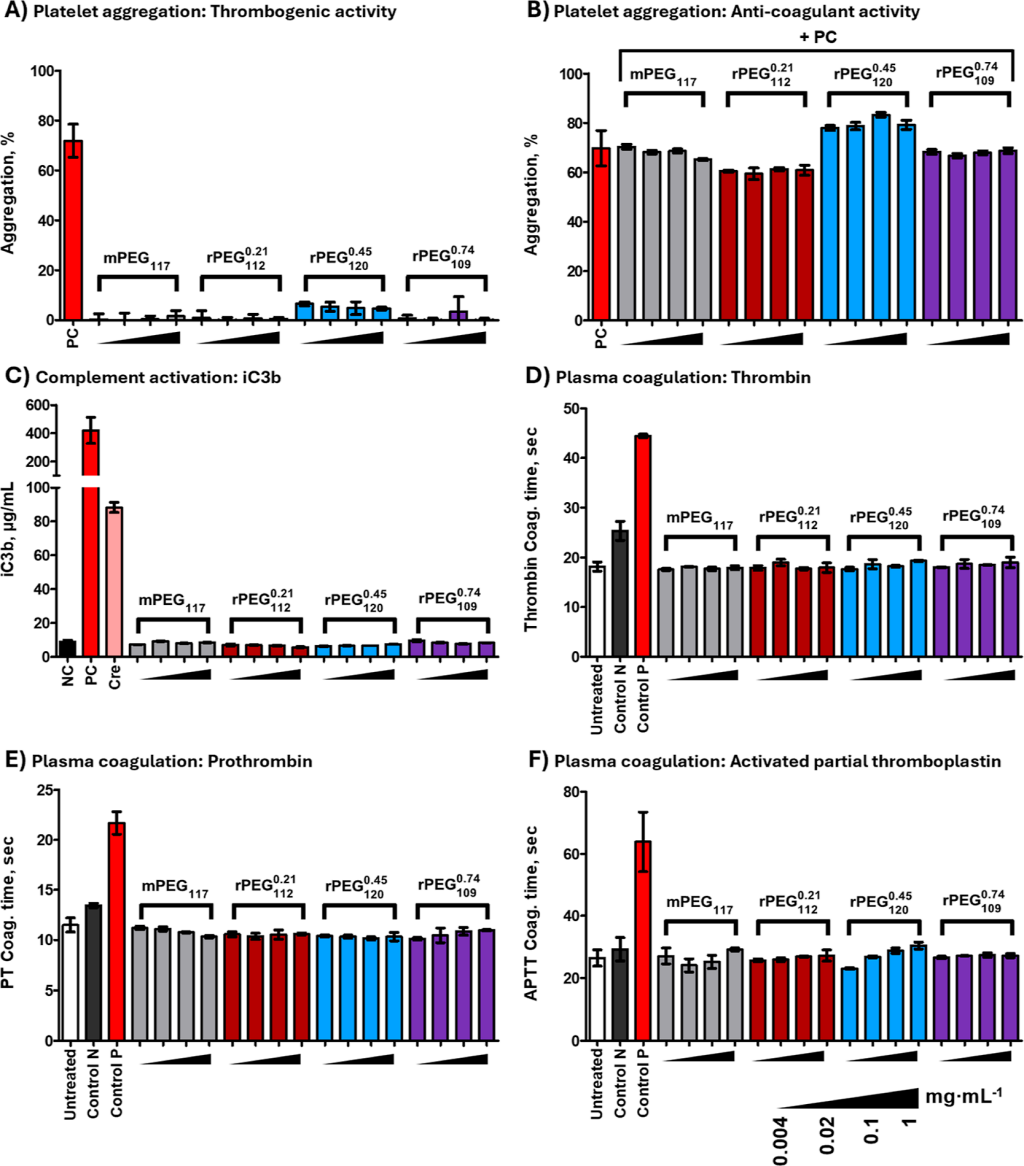
Assessing rPEG hemocompatibility in human blood in vitro. mPEG_117_, rPEG_112_^0.21^, rPEG_120_^0.45^ and rPEG_109_^0.74^ were evaluated for their potential to induce/inhibit platelet aggregation, activate the complement system and affect normal plasma coagulation times. Each sample was tested at four concentrations (0.004–1 mg mL^–1^). Two (complement assay) or three (all other tests) independent samples were prepared for each concentration and analyzed in duplicate (% CV < 20 for all assays and % CV < 5 for plasma coagulation assay). Shown is the mean response ±SD (A,B) Platelet aggregation: (A) particles alone were tested to verify their potential thrombogenic effect; (B) in combination with the assay positive control collagen (PC) to assess their anticoagulant activity. (C) Complement activation. PBS was used as the negative control (NC). Cobra venom factor (PC), and Cremophor-EL (Cre) were used as positive controls. (D–F) Plasma coagulation was assessed in thrombin, prothrombin (PT), and activated partial thromboplastin (APTT) time assays. WHO standard normal (Control N) and abnormal plasma (Control P) were used to qualify the instrument’s performance.

Based on the reactivity ratios determined in DMSO-*d*_6_ (*r*_EO_ ≈ *r*_GME_ ≈ 1) and considering an epitope of 16 or more consecutive EO repeating units, calculations and simulations with different molar compositions were performed (Supporting Information) to illustrate the random distribution of the ‘synthetic point mutations’ along the rPEG chains (Figures 6A,B, S62–S64). The targeted chemical heterogeneity within the polymer sample is evident from the simulation, as each distinct chain possesses a different sequence of repeating units (Figure 6B). The methoxy methylene groups as “synthetic point mutations” are randomly distributed in the polyether backbone, and the statistical diversity within each chain within one sample is tremendously high. As an example, for a copolymer with 114 monomer units, among which 23 monomer units are GME (20 mol %), the number of possible chain isomers with different monomer sequences is 7.3 × 10^23^.

**Figure 6:**
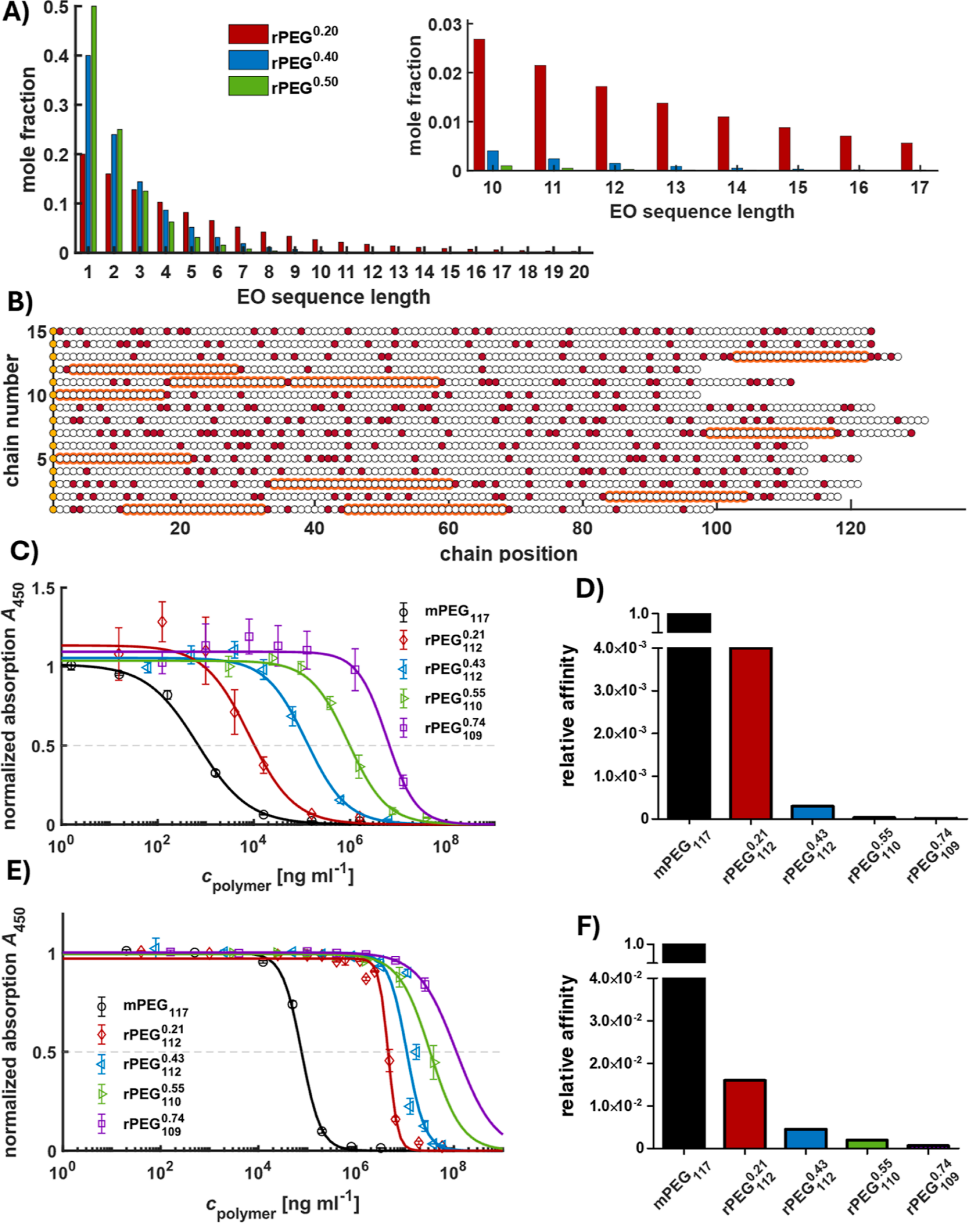
Calculation and influence of ‘synthetic point mutations’. (A) Mole fractions of EO sequence lengths for different comonomer compositions; (B) section of 15 chains from the simulation (10^4^ chains) of EO (white) and GME (red) repeating unit distribution at different chain positions of rPEG_114_^0.20^; repeating patterns with 16 consecutive EO repeating units are highlighted in orange; yellow circles represent the initiator; (C) backbone-selective APA ELISA of investigated rPEGs and mPEG_117_; the dashed gray line represents the half-maximal effective concentration (EC_50_) value; (D) relative affinity (1/EC_50_) of backbone-selective APA for investigated rPEGs and mPEG_117_ based on EC_50_ values obtained from backbone-selective APA ELISA; (E) end group-selective APA ELISA of investigated rPEGs and mPEG_117_; the dashed gray line represents the EC_50_ value; (F) relative affinity of end group-selective APA for investigated rPEGs and mPEG_117_ based on EC_50_ values obtained from end group-selective APA ELISA.

This illustrates that the induction of polymer-selective antibodies targeting a regular chain segment should be highly hampered or even impossible. The calculations show that a decrease in the molar fractions of longer, merely EO containing segments with increasing GME amount occurs (Figure 6A). This is confirmed by the simulations (Figures 6B, S62–S64) where the probability of finding at least one repeating pattern of 16 or more consecutive EO repeating units (highlighted in orange) in a rPEG chain decreases from 46% for rPEG_114_^0.20^ to below 0.1% in the case of rPEG_114_^0.50^ (Table S8). Besides the decrease in consecutive EO units, the increased spatial requirement of rPEGs and the random distribution of methoxy methylene side chains should additionally impede or even disable interaction with APAs according to the specific “lock and key principle”. For rPEGs, the GME monomer is utilized as a racemic mixture. In addition to employing two different comonomers and their sequence in the polymer chain, the stereochemistry of GME introduces another randomization parameter within one sample, rendering adaptation of the immune system and antibody formation even more improbable. This sets our system further apart from other approaches using hydrophilic homopolymers.

To investigate our hypothesis regarding APAs, we tested the effect of a varied concentration of synthetic point mutations (0–74 mol % GME) on the binding capability of backbone- and end group-selective APAs via competitive ELISA. The concentration-dependent interaction between the APA and polyether is detected by a decrease of absorbance intensity. In summary, the backbone-selective APA ELISA results confirm the effect of the incorporation of methoxy methylene groups along the PEG backbone on the APA recognition, in alignment with the simulations.

A major increase of the half maximal effective concentration (EC_50_) value is observed with increasing GME content. Comparing the relative affinities (1/EC_50_) of the APA toward mPEG_117_ and rPEGs (Figure 6C,D, Table S9) underlines the significance of the diminished antigenicity. With incorporation of 21–74 mol % GME within the polyether, its affinity drops to 0.4 to 4.6 × 10^–6^% relative to mPEG_117_, respectively. Following the simulations, the random nature of “synthetic point mutation” incorporation in the PEG chains reduces the length of undisrupted PEG segments, thereby minimizing the probability of forming a PEG epitope. Nevertheless, binding of the APA is still observed at very high rPEG concentrations. We consider two possible explanations for this behavior: (i) an interaction of a smaller epitope may already cause a weak recognition; (ii) the initial interaction between APA and PEG is based on van der Waals forces before trapping the polymer by a conformational change.^24^ At polymer concentrations as high as tested, an unspecific aggregation or entanglement of polymer and APA could cause a decrease in binding affinity to the competitive PEG coated on the ELISA plate wells. Considering the overall polymer portion of PEGylated therapeutics, antigenic concentrations of rPEGs in the performed studies are magnitudes higher than those of PEG in current clinical applications.^37^ While the introduction of pendant methoxy methylene groups strongly reduces the antigenicity of rPEGs toward backbone-selective APAs, this assay does not provide information regarding end group-specific APA interactions. In principle, each GME repeating unit contains a methoxy group which could theoretically be detected by the end group-selective APAs. To address this, a separate ELISA assay was conducted to evaluate interaction of the end group-selective APA with the rPEG polymers.

Compared to the backbone-selective APA, the EC_50_ value of mPEG_117_ in the end group-selective APA assay is shifted to higher concentrations, indicating an overall lower polymer affinity. In accordance with the backbone-specific APA ELISA, a notable shift of EC_50_ values of all rPEGs in relation to mPEG_117_ is observed (Figure 6E). The relative APA affinity drops to 1.6 to 6 × 10^–2^% (Figure 6F, Table S9). While the EC_50_ value of rPEG_112_^0.21^ significantly differs from mPEG_117_, a further increase of the GME content has a minor impact on the APA binding relative to rPEG_112_^0.21^. This can be explained with the two different binding domains of the end group-selective APA. The APAs’ end group binding domain cannot contribute to the overall binding, as all rPEGs are furnished with a blocked α-chain end (Figure 1A). Consequently, merely the domain that interacts with PEG_7_ sequences along the polymer backbone can mediate binding. Since the change in affinity is significantly affected by blocking of the end group binding domain, this appears to be the primary binding site for the end group-selective APA. The results further confirm that the methoxy groups of the side chains do not enable binding of the end group-selective APAs, in stark contrast to a methoxy end group.

## Conclusion

The increasing abundance of anti-PEG antibodies (APA) in the population leads to undesired immune responses to PEGylated products, thereby altering their efficacy (e.g., via inducing premature drug release and causing accelerated blood clearance) and safety (e.g., by activating the complement system and innate immune responses). This has become an increasingly severe issue over the years, since it renders the stealth effect of PEG ineffective. Recently, this issue has increased drastically, as the containment of the global COVID-19 pandemic strongly relied on PEGylated lipids for the transport of mRNA vaccines. We introduce the concept of random polyether copolymers that are structural PEG isomers, i.e., randomized PEGs (rPEG), containing ‘synthetic point mutations’ as a key alternative to PEG for applications in therapeutic nanomedicine.

Detailed in situ ^1^H NMR kinetics experiments revealed an ideally random introduction of sterically demanding branching points in PEG by random copolymerization of EO and the comonomer glycidyl methyl ether (GME). The highly hydrophilic rPEGs demonstrate drastically reduced recognition by both backbone- and end group-selective anti-PEG antibodies at pharmaceutically relevant concentrations in competitive enzyme-linked immunosorbent assays (ELISA). Simulations and calculations regarding the microstructure of such copolymers further support the experimental ELISA results. A wide structural diversity, due to the statistical copolymerization process, is present in rPEG samples, despite the precise control of chain length and excellent end group fidelity. This circumvents the formation of structural regularity within the polymer chains. Minimizing identifiable, repeated features should highly impede selective recognition of the polymer.

The presented rPEG strategy can rely on PEG technology at every stage, enabling the application of established good manufacturing practice (GMP) and integration into existing supply chains. Versatile conjugation chemistry has been established for PEG for more than three decades and is, in principle, fully transferable to rPEG. Furthermore, applying rPEGs exclusively for medical and pharmaceutical purposes, distinct from PEG’s widespread use in everyday products such as surfactants, can be advantageous and help avoid undesirable side effects triggered by the anti-PEG antibodies. It remains to be determined whether the immune system can effectively adapt to a deliberately heterogeneous system and generate anti-rPEG antibodies. This important question is the focus of ongoing studies. We firmly believe that this unprecedented statistical variation approach bears universal potential to suppress undesired recognition for nanomedicine treatments ranging from protein bioconjugation to PEGylated nanocarriers such as LNPs.

## Supplementary Material



## Notes

Additional analytical data (NMR, SEC, MALDI TOF MS, HPLC), biocompatibility data (ELISA, cytokine release) and experimental procedures and methods and supplementary text and simulation plots (PDF)
