# Supplementary material for: Isomerization of Poly(ethylene glycol): A Strategy for the Evasion of Anti-PEG Antibody Recognition
Source: J Am Chem Soc. 2025 Jun 13;147(25):21538–48. doi: 10.1021/jacs.5c02716 (PMC12203584; doi:10.1021/jacs.5c02716)
Supplement: Supplementary file 1 [file ja5c02716_si_001.pdf]

Supporting Information for

Isomerization of poly(ethylene glycol): A strategy for the evasion of anti-PEG antibody recognition

Authors: Philip Dreier[1,†], Rebecca Matthes[1,†], Fabian Fuß[1,†], Julian Schmidt[1,†], Dominik Schulz[1], Gregor M. Linden[1], Ramona D. Barent[1], Sandra Schüttner[1], Barry W. Neun[2], Edward Cedrone[2], Marina A. Dobrovolskaia[2], Matthias Bros[3], Holger Frey[1,*]

Affiliations:

[1]Department of Chemistry, Johannes Gutenberg University, Mainz; 55128 Mainz, Germany

[2]Nanotechnology Characterization Lab., Cancer Research Technology Program, Frederick National Laboratory for Cancer Research sponsored by the National Cancer Institute, 8560 Progress Dr., Frederick, MD, 21701 USA

[3]University Medical Centre, Johannes Gutenberg University; 55101 Mainz, Germany

*Corresponding author. Email: hfrey@uni-mainz.de

†Philip Dreier, Rebecca Matthes, Fabian Fuß and Julian Schmidt contributed equally to this work.

1. Materials and Instrumentation

1.1. Reagents and Equipment

All chemicals and solvents were purchased from Acros Organics, Roth, TCI, Sigma-Aldrich, Fisher Scientific, BLDpharm and Fluka, unless otherwise noted. Deuterated solvents were received from Deutero GmbH. Ethylene Oxide was acquired from Air Liquide. THF was flashed over basic aluminum oxide before usage. Glycidyl methyl ether was dried over CaH_2 and cryo-transferred before polymerizations.

1.2. NMR spectroscopy

^{1}H and ^{13}C NMR spectra were recorded on a Bruker Avance III HD 400 spectrometer with 400 and 100 MHz, respectively, and referenced internally to residual proton signals of the deuterated solvent. All spectra were acquired at 23 °C. Spectra were processed and analyzed utilizing the *MestReNova 14.3.3-33362* software.

in situ ^{1}H NMR kinetics: Polymerization kinetics was conducted on a *Bruker Avance III HD* (400 MHz) with a 5 mm nitrogen-cooled BBO-cryoprobe-head (BB+H+F) with *z*-gradient, automated tune and match (ATM) and autosampler SampleXPress 60. All spectra were acquired at temperatures stated in Table S1 and Table S2. Spectra are referenced to the residual protons of the solvents used. Data were processed with *MestReNova 14.3.3-33362* software. For kinetics in anisole (non-deuterated), lock was turned off. Shim was performed with TopShim (gradient shim), using the protons of the anisole methoxy group. Sample spinning was turned off and one spectrum was recorded to acquire the receiver gain. Afterwards, one spectrum was recorded every two minutes. The respective resonances of the monomers were used to track their consumption (δ(EO) = 2.26–2.31 ppm, δ(GME) = 2.35–2.43 ppm). To analyze the normalized monomer consumption, the software NIREVAL was used.[1]

Diffusion-ordered NMR spectroscopy (DOSY NMR): Polymer samples were measured in D_2O at a concentration of 1 mg mL^{-1}. DOSY measurements were conducted on a *Bruker Avance III HD* (400 MHz) with a 5 mm nitrogen-cooled BBO-cryoprobe-head (BB+H+F) with *z*-gradient, automated tune and match (ATM) and autosampler SampleXPress 60. A total of 8 scans were acquired. The time domain consisted of 64 data points. The diffusion delay was set to 0.2 s. Additionally, the duration of each gradient pulse was 2500 µs. All spectra were acquired at 23 °C. Spectra are referenced to the residual protons of the solvents used. Spectra were processed and analyzed utilizing the *MestReNova 14.3.3-33362* software.

Quantitative ^{31}P-NMR (^{31}P-IG NMR) spectroscopy[2]*:* Samples of 30 – 50 mg polymer were dissolved in 0.6 mL $CDCl_3$/Pyridine-H_5 (1.6/1, *V*/*V*) stock solution containing 13.98 µmol mL^{-1} cyclohexanol as internal standard and 0.19 µmol/mL tris(acetylacetonato)chromium(III) (Cr(acac)$_3$). 2-Chloro-4,4,5,5-tetramethyldioxaphospholane was added directly into the NMR tube in an 8.5-fold excess per hydroxy functionality. After a minimum reaction time of 30 min, the ^{31}P-IG measurements were conducted on a *Bruker Avance III HD* 400 spectrometer operating at a resonance frequency of 162.1 MHz with a 5 mm nitrogen-cooled BBO-cryoprobe-head (BB+H+F) with *z*-gradient, automated tune and match (ATM) and autosampler SampleXPress 60. To ensure quantitative accuracy, the nuclear Overhauser effect (NOE) was suppressed with an inverse gated proton decoupling pulse sequence. The standard ^{31}P-IG NMR acquisition parameters were a 90° pulse width of 12.0 µs, a spectral width of 15 kHz, and a relaxation delay of 30 s. Data were acquired with 16k points and zero-filled to 32k prior to processing. A total of 64 scans at 23 °C were acquired. A 3rd order polynomial fit was applied before integration. All spectra were referenced to the hydrolysis product of 2-chloro-4,4,5,5-tetramethyldioxaphospholane at 132.2 ppm. The sharp signal at 174.9 ppm ensures the

excess of 2-chloro-4,4,5,5-tetramethyldioxaphospholane. Spectra were processed and analyzed utilizing the *MestReNova 14.3.3-33362* software.

1.3. Gel permeation chromatography (GPC)

Measurements were conducted using an Agilent 1100 series HPLC system, which included a degasser, isocratic pump (G1310A), autosampler (G1313A), column oven (G1316A), and detectors for refractive index (RI) (G1310A) and variable wavelength (VWD) (G1314A). Separations were carried out employing a four-column set-up (*MZ-Analysentechnik GmbH*) connected sequentially:

i) HEMA-40 guard column (40 Å pore size, 10 µm particle size, 50x8.0 mm)
ii) HEMA-40 analytical column (40 Å pore size, 10 µm particle size, 300x8.0 mm)
iii) HEMA-100 analytical column (100 Å pore size, 10 µm particle size, 300x8.0 mm)
iv) HEMA-300 analytical column (300 Å pore size, 10 µm particle size, 300x8.0 mm)

The eluent consisted of DMF (Fisher Chemical) with 1 mg mL^{-1} anhydrous LiBr (Acros Organics), delivered at a flow rate of 1 mL min^{-1}. Both the column oven and RI detector cell were maintained at 50 °C. Calibration was performed using well-defined poly(ethylene glycol)s from PSS (PSS Standards Kit) with molar mass values (M_p) ranging from 106 to 42700 g mol^{-1}. Samples were dissolved in DMF (with 1 mg mL^{-1} anhydrous LiBr) at a concentration of 1 mg mL^{-1} with the addition of 1 drop of toluene. The injection of 100 µL of the stock solutions was carried out via the autosampler, with a measurement duration of 45 min. Elution times were referenced using toluene as an internal standard. RI traces were analyzed using the *PSS WinGPC Unichrom V8.31* software.

1.4. MALDI-ToF Mass Spectrometry (MS)

MALDI-ToF MS measurements were carried out using a Bruker autoflex maX MALDI-ToF/ToF using a smartbeam-II solid state laser with a wavelength of 337 nm. Spectra were recorded using the software *Bruker flexControl 3.4* and analyzed using *Bruker flexAnalysis 3.4* and *Bruker polytools 1.31*. The potassium salt of triflouroacetic acid (KTFA) and *trans*-2-[3-(4-*tert*-butylphenyl)-2-methyl-2-propenylidene]malononitrile (DCTB) were utilized as ionization salt and matrix, respectively. For sample preparation, the polymers were dissolved in chloroform at 10 mg mL^{-1}. 20 µL of this solution were combined with 20 µL of a 10 mg mL^{-1} solution of the matrix in chloroform. 5 µL of a 0.1 M solution of the salt in methanol were added and 1 µL of the resulting mixture was spotted onto a MTP 384 ground steel target plate. The solvents were allowed to evaporate completely before the measurement. All measurements were performed in linear mode.

1.5. High-performance liquid chromatography (HPLC)

Analytical Protocol: The HPLC system consisted of an Agilent Technologies 1260 Infinity system with a 1260 Quat pump, a 1260 ALS autosampler, a 1260 VWD UV–vis variable-wavelength spectrophotometric detector, a Softa 1300 evaporative light scattering detector (ELSD), a PSS TCC6000 column thermostat and a solvent degasser. The UV detector was operated at a wavelength of 254 nm. The ELSD was operated with a spray chamber temperature of 30 °C, a drift tube temperature of 60 °C and an air pressure of 3.0 bar. The column oven temperature was set to 30 °C. A flowrate of 1 mL min^{-1} was applied. For analysis, a PerfectSil 300 (C_4, 5 µm particle size, 250 × 4.6 mm (L × ID) column from MZ-Analysentechnik GmbH was used. The mobile phases consisted of MeOH (HiPerSolv Chromanorm (HPLC gradient grade) and H_2O (LiChrosolv® Merck Chemicals, LC-MS grade). Samples were dissolved in H_2O at a concentration of 1 mg mL^{-1}, filtered through a Rotilabo® syringe filter (PTFE, pore size 0.45 µm). 25-50 µL of the stock solution was injected and analyzed by RP-HPLC with a MeOH/H_2O gradient:

i) 0-5 min: isocratic at 60% MeOH

ii) 5-15 min: linear gradient from 60 to 100% MeOH
iii) 15-20 min: isocratic at 100% MeOH

Subsequently, the starting gradient was restored within 1 min and a 20 min reconditioning time at 60% MeOH was allowed before further analysis. ELSD elugrams were analyzed using the *PSS WinGPC Unichrom V8.31* software.

Semi-preparative Protocol: The HPLC system consisted of an Agilent Technologies 1260 Infinity system with a 1260 Quat pump, a 1260 ALS autosampler, a 1260 VWD UV−vis variable-wavelength spectrophotometric detector, a solvent degasser and a fraction collector (ADVANTEC CHF122SC). The UV detector was operated at a wavelength of 254 nm. A flowrate of 10 mL min^{-1} was applied. A PerfectSil 300 (C_4, 5 µm particle size, 250 × 20 mm (L × ID) column from MZ-Analysentechnik GmbH was used. The mobile phases consisted of MeOH (HiPerSolv Chromanorm (HPLC gradient grade) and H_2O (LiChrosolv® Merck Chemicals, LC-MS grade). Samples were dissolved in H_2O at a concentration of 200 mg mL^{-1}, filtered through a Rotilabo® syringe filter (PTFE, pore size 0.45 µm). 500 µL of the stock solution was injected and purified by RP-HPLC with a MeOH/H_2O gradient:

i) 0-10 min: isocratic at 60% MeOH
ii) 10-30 min: linear gradient from 60 to 100% MeOH
iii) 30-60 min: isocratic at 100% MeOH

Subsequently, the starting gradient was restored within 1 min and a 40 min reconditioning time at 60% MeOH was allowed before further purification runs. Each fraction consisted of a volume of 30 mL. Product fractions were visualized via consecutive tapping of six drops of each fraction on a silica gel TLC plate (Sigma Aldrich), subsequent staining with potassium permanganate solution and heating of the plate.

$PGME_{103}$ was purified using a JAI LC-91XX NEXT Recycling Preparative GPC using a JAIGEL-4H column and chloroform as a solvent at 30 °C and a flowrate of 3.5 mL min^{-1}. The sample was dissolved in chloroform at 300 mg mL^{-1} before injection. Elution time was determined at approximately 22 min.

1.6. Turbidimetric Measurements

Turbidimetric measurements were performed with a *JASCO* UV-Vis Spectrometer (V730) at a light wavelength of 600 nm and a heating rate of 1 K min^{-1} using the software *JASCO Spectra Manager Ver.2*. Polymers were dissolved in PBS buffer solution (pH = 7.4) at various concentrations. Pure PBS buffer solution was utilized as a reference value of 100% transmittance and was measured prior to each experiment. All measurements were performed in a quartz glass cuvette from Hellma Analytics with a light path of 10 mm. Cloud point temperatures (T_{cp}) were determined at a transmittance of 50%. Raw data was normalized to maximum and minimum values of the respective heating curves.

1.7. Enzyme-linked Immunosorbent Assay (ELISA)

Competitive backbone-selective anti-PEG antibody ELISA: The assessment of anti-PEG antibody affinity for the respective polymer involved performing assays on mPEG (20 kDa)-BSA coated 96-well plates. The LifeDiagnostics anti-PEG-ELISA Kit, including 3,3′,5,5′-Tetramethylbenzidin (TMB) staining solution, stop-solution, anti-PEG antibody HRP-conjugate (clone 1D9-6) and dilution buffer was utilized. Absorption measurements were conducted with a BMG Labtech FLUOstar Omega multi-mode reader at a wavelength of 450 nm and all samples were measured in triplicates. The data acquired was analyzed utilizing both Omega Software and OriginPro 8. To create samples of varying concentrations, 10 mg of polymer was dissolved in dilution buffer and aliquots were prepared through a dilution series. The anti-PEG antibody (9 µL) was dissolved in 12 mL of dilution buffer. The wash buffer concentrate (50 mL) was diluted with MilliQ water to yield 1 L of wash buffer. In the assay process, 50 µL of each polymer solution was added to the coated 96-well plate, followed by

50 µL of the anti-PEG antibody solution. The samples were incubated for 1 h at 25 °C with shaking at 300 rpm. After discarding the solution, each well underwent six washes with 300 µL of wash buffer. Excess wash buffer was removed by gently tapping the plate on the bench. Subsequently, 100 µL of TMB staining solution was added to each well, followed by a 20 min incubation at 25 °C with shaking at 300 rpm. The reaction was quenched by adding 100 µL of stop-solution to each well and immediate absorption measurements were taken. The determined absorbance values were normalized to visualize the percentage of maximal binding. The sample concentrations were transformed to a function of $\log_{10}$. The sigmoidal fits were calculated using the following equation with A_2 representing the upper limit, A_1 the lower limit, c the inflection point, and d the hill slope.

$$y = A_1 \frac{A_2 - A_1}{1 + 10^{(c-x)d}} \qquad \textbf{(S1)}$$

Competitive end group-selective anti-PEG antibody ELISA: The assessment of anti-mPEG antibody affinity for the respective polymer involved performing assays on mPEG (20 kDa)-BSA coated 96-well plates. A LifeDiagnostics anti-mPEG-ELISA kit, including TMB staining solution, stop-solution, anti-mPEG antibody HRP-conjugate (clone 5D6-3) and dilution buffer was utilized. Additionally, a mPEG (20 kDa)-BSA coated 96-well plate from the anti-PEG-ELISA Kit of LifeDiagnostics was used. Absorption measurements were conducted with a BMG Labtech FLUOstar Omega multi-mode reader at a wavelength of 450 nm. All samples were measured in triplicates. The data acquired was analyzed utilizing both Omega Software and OriginPro 8.

To create samples of varied concentrations, 10 mg of polymer was dissolved in dilution buffer and aliquots were prepared through a dilution series. The anti-mPEG antibody (9 µL) was dissolved in 12 mL of dilution buffer. The wash buffer concentrate (12.5 mL) was diluted with MilliQ water to yield 250 mL of wash buffer. In the assay process, 50 µL of each polymer solution was added to the coated 96-well plate, followed by 50 µL of the anti-mPEG antibody solution. The samples were incubated for 1 h at 25 °C while shaking at 300 rpm. After discarding the solution, each well underwent six washes with 300 µL of wash buffer. Excess wash buffer was removed by gently tapping the plate on the bench. Subsequently, 100 µL of TMB staining solution was added to each well, followed by a 20 min incubation at 25 °C with shaking at 300 rpm. The reaction was quenched by adding 100 µL of stop-solution to each well and immediate absorption measurements were taken. The determined absorbance values were normalized to visualize the percentage of maximal binding. The sample concentrations were transformed to a function of $\log_{10}$. The sigmoidal fits were calculated using equation S1.

1.8. <u>Cell viability assays</u>

Cell viability and immune cell immunophenotypes were analyzed using fluorescence-activated cell sorting (FACS). mPEG and rPEGs were purified by semi-preparative HPLC to ensure endotoxin-free samples and lyophilized prior to analysis. The viability of primary immune cells and expression of activation markers CD80 and CD86 were determined using human PBMCs. PBMC isolation was performed under sterile conditions using a laminar flow bench. Fluorescent-dye labeled antibodies for flow cytometric analysis were purchased from Thermo Fisher Scientific, BD Bioscience or BioLegend. Human whole blood (CPD-stabilized, citrate-phosphate buffer) of healthy donors was received from the transfusion central of the University Medical Centre of the Johannes Gutenberg University Mainz. The PBMCs were isolated using the common density gradient technique. In detail 20 mL Histopaque-1077 (1.077 g mL^{-1}, Sigma Aldrich) were carefully under-layered with a mixture of 10 mL blood pre-diluted in 10 mL DPBS in 50 mL tubes. Afterward, the tubes were centrifuged (20 min, room temperature, 700 g) with break switched off. The plasma phase and the PBMC

interphase were separately retrieved. The Plasma was heat-inactivated for at least 30 min at 56 °C, centrifuged (10 min, 4 °C, 1500 g) and stored at 4 °C until further usage. The PBMCs of two tubes from one donor (total blood volume 20 mL) were combined in a new 50 mL tube, which was then filled up to 50 mL with DBPS. The mixture was centrifuged (10 min, 4 °C, 600 g) and the supernatant was discarded. The cell pellet was resuspended in fresh DPBS and again filled up to 50 mL with DPBS. This washing procedure was performed five times. Afterwards, the PBMCs were resuspended in RPMI-1640 culture medium (+100 U/mL penicillin, +100 U/mL streptomycin, +50 µM 2-mercaptoethanol, +2 mM L-glutamine) at a concentration of 5 x 10^6 cells mL^{-1}. Aliquots of 0.450 mL were transferred into FACS tubes. mPEG and rPEG formulations were dissolved in DPBS and added to final concentrations of 0.1, 1.0 and 5.0 mg mL^{-1} to the cell suspensions. Additionally, resiquimod (R848, 0.1 µg mL^{-1}) was prepared in parallel as a positive control to assess immunostimulatory effects. After 16 h of incubation (37 °C, 7.5% CO_2), the samples were centrifuged (10 min, 4 °C, 300 g) and 100 µL aliquots of supernatants were collected and stored at -20 °C for cytokine analysis. Each cell pellet was resuspended in 1 mL human FACS buffer (DPBS, 2 mM EDTA, 0.5 vol% fetal calf serum), then all samples were centrifuged (10 min, 4 °C, 300 g) and the supernatants were discarded. Afterward, the Fc receptors were blocked for 10 min at 4 °C using 5 µL human FcR blocking reagent (Miltenyi Biotec) to prevent non-specific binding. Lineage and immune activation markers were detected using fluorescence-label antibodies specific for CD80 (PerCP-eFluor710), CD15 (APC), CD20 (V450), CD11b (BV510), CD1c (BV605), CD3 (BV711), CD86 (PE), CD14 (PE-eFluor610) and CD56 (PE-Cy7) for 20 min at 4 °C. The samples were washed twice with 1 mL human FACS buffer. The cells were resuspended in 0.5 mL human FACS buffer and stored at 4 °C until measurement (maximum 3 hours). 0.5 mL of the viability dye solution (30 nM, Sytox™ Green™) was added 15 min before the measurement of each donor. Each sample was stored on ice until immanent flow cytometric measurements using an Attune NxT acoustic focusing cytometer (Lasers: BRVK, Instrument model: 4486521, Thermo Fisher Scientific) with Attune Nxt Software v3.2.1526.0. The raw data was evaluated with Attune NxT software v3.1.1 according to the gating strategy depicted in Figure S65. The exclusion of doublets was applied after the lineage gating to improve the detection of low numbers of small cells in the heterogeneous cell mixture.[3] Cell viability as well as CD80 and CD86 mean fluorescence intensity (MFI) were normalized to the untreated control for each respective donor. The mean values and the standard deviation were visualized using GraphPad Prism 5.

1.9. Human Inflammatory Cytokine Cytometric Bead Array (CBA)

Interferon-γ (INF-γ), tumor necrosis factor-α (TNF-α), interleukin-1β (IL-1β), interleukin-6 (IL-6) and interleukin-10 (IL-10) were quantified using a BD Bioscience Cytometric Bead Array Flex Kit with minor adjustments according to the manufacturer's protocol. A standard calibration containing all five cytokines was prepared in CBA buffer (DPBS +1.0 vol-% fetal calf serum) comprising 2500, 1250, 625, 312.5, 156, 80, 40, 20, 10 and 0 pg mL^{-1}. A capture bead master mixture was prepared by mixing 0.2 µL/sample of each capture bead with 10 µL/sample CBA buffer. 10 µL of each sample were added to 10 µL master bead mixture and incubated for one hour, in the dark at room temperature. Afterward, 10 µL of the detection reagent master mixture (0.2 µL/sample of each detection reagent in 10 µL/sample CBA buffer) were added and incubated for two hours, in the dark at room temperature. 1 mL of CBA buffer was added to the samples. The tubes were centrifuged (5 min, 4 °C, 300 g) and the supernatants were carefully discarded. 200 µL CBA buffer were added to each sample just before the measurement. The MFI values of each bead were recorded using an Attune NxT acoustic focusing cytometer (Lasers: BRVK, Instrument model: 4486521, Thermo Fisher Scientific) with Attune Nxt Software v3.2.1526.0. The FCS 3.0 files were evaluated using FCAP Array (v1.0.1). The standard calibration curves were calculated with a five-parameter logistic fit function and the respective cytokine concentrations were determined. Given the significant variability in immune activity among individual donors, the cytokine concentrations were normalized to each donor's R848

positive control (16 h PBMC Incubation with 1 μg mL^{-1} R848). The mean values and the standard deviation were visualized using GraphPad Prism 5.

$$y = D \frac{A - D}{1 + \left(x - \frac{E}{C}\right)^{B}} \quad \textbf{(S2)}$$

1.10. Hemocompatibility assessment

Research donor blood:

Institutional Review Board (IRB)-approved National Cancer Institute (NCI)-Frederick Protocol OH99-CN-046 was followed to obtain fresh blood from healthy donor volunteers. The blood was collected into vacutainers containing anticoagulant. The blood was processed within 2 hours after collection to generate and pool plasma from at least three volunteers.

Cytokine Secretion Assay:

Experiments were performed according to the NCL protocols ITA-10 (Preparation of Human Whole Blood and Peripheral Blood Mononuclear Cell Cultures) and ITA-27 (Multiplex ELISA for Detection of Human Cytokines).[3–5] Briefly, whole blood anticoagulated with lithium heparin was used to purify PBMC. Afterwards, the PBMCs were incubated with controls, mPEG as well as rPEG samples for 24 h. At the end of incubation, the samples were centrifuged for 5 min at 18000 g and supernatants were analyzed for the presence of cytokines and interferons using multiplex ELISA kits (Quansys, Logan, UT).

Complement activation:

The pooled K3-EDTA plasma was exposed to controls and test samples in the presence of veronal buffer for 30 min at 37 °C according to the NCL protocol ITA-5.2.[3,5] PBS was used as a negative control (NC), cobra venom factor (PC) and Cremophor-EL (Cre) were used as positive controls. Test samples were analyzed at four concentrations (0.004 – 1 mg mL^{-1}). Two independent samples were prepared for each test condition and analyzed in duplicate on an ELISA plate. The complement activation was monitored by measuring complement split product iC3b in supernatants by MicroVue-TM EIA kit (Quidel, San Diego, CA, USA) according to the manufacturer's instructions. Positive and negative control samples were included on each ELISA plate to normalize for potential plate variability.

Platelet Aggregation Assay:

The analysis was conducted according to the NCL protocol ITA 2.1.[6] Briefly, platelet-rich plasma (PRP) and platelet-poor plasma (PPP) were prepared from freshly drawn human blood anticoagulated with sodium citrate (Na-Cit). Plasma from three donors was pooled. PPP was used as the background control. PRP was incubated with test samples at four concentrations (0.004 – 1 mg mL^{-1}), and the number of single platelets was counted using a Beckman Coulter Z2 analyzer. Platelet-poor plasma combined with nanoparticles was used to monitor potential particle aggregation/agglomeration in order to rule out false-negative results. The percent platelet aggregation was calculated by comparing the number of single (unaggregated) platelets in the negative control group with that in the test sample.

Plasma Coagulation Assay:

The analysis was conducted according to the NCL protocol ITA 12.[7] Briefly, three plasma coagulation tests —prothrombin time (PT), activated partial thromboplastin time (APTT), and thrombin time (TT), corresponding to the extrinsic, intrinsic, and common pathways, respectively— were performed. Freshly drawn human blood anticoagulated with sodium citrate from three donors was used to prepare pooled plasma. The pooled plasma was then incubated with test samples at four

concentrations (0.004 – 1 mg mL^{-1}) for 30 min at 37 °C. Following incubation, plasma coagulation initiation reagents (neoplastin, $CaCl_2$, or thrombin, respectively) were added to the mixture, and the coagulation times were measured using the STArt4 coagulometer (Diagnostica Stago). Three individual samples were prepared at each test concentration and analyzed in duplicate. WHO standard normal (Control N) and abnormal plasma (Control P) were used to qualify the instrument performance.

2. Experimental Procedures

2.1. Synthesis of 1-chloro-3-methoxy-propan-2-ol

A 1 L three-necked flask equipped with neodym stirrer bar, reflux condenser and dropping funnel was charged with MeOH (306 g, 388 mL, 9.57 mol) and sulfuric acid (98%, 7 mL, 125 mmol). The flask was immersed in an oil bath and epichlorohydrin (295 g, 250 mL, 3.19 mol) was added dropwise through the dropping funnel to the solution under vigorous stirring. After complete addition of epichlorohydrin, the reaction was heated to reflux and stirred overnight under reflux. The reaction mixture was cooled to room temperature and $BaCO_3$ (37.0 g, 187 mmol) was added under vigorous stirring. After 1 h of stirring, excess MeOH was evaporated under reduced pressure. 1-Chloro-3-methoxy-propan-2-ol (320 g, 2.60 mol, 81%) was obtained as colorless liquid after fractional distillation (T_b = 83-87 °C (85 mbar)) of the residue. ^1H and ^{13}C NMR spectra can be found in Figure S2 and Figure S3, respectively.

^{1}H NMR (400 MHz, DMSO-*d*$_6$) δ [ppm]: 5.26 (d, J = 5.3 Hz, 1H), 3.87 – 3.71 (m, 1H), 3.61 (dd, J = 11.0, 4.5 Hz, 1H), 3.51 (dd, J = 11.0, 5.6 Hz, 1H), 3.33 (dd, 2H), 3.26 (s, 3H).

^{13}C NMR (100 MHz, DMSO-*d*$_6$) δ [ppm]: 73.53 (CH_2), 68.93 (CH), 58.51 (CH_3), 47.12 (CH_2).

2.2. Synthesis of glycidyl methyl ether (GME)

1-Chloro-3-methoxy-propan-2-ol (320 g, 2.57 mol) was added to a three-necked flask equipped with mechanical stirrer, thermometer and reflux condenser. Diethyl ether (320 mL) was added and the solution was cooled to 0 °C with an ice bath under stirring. Sodium hydroxide (123 g, 3.08 mol) was added portion wise to the solution under vigorous stirring while the temperature was kept below 15 °C. The reaction mixture was allowed to reach room temperature over 4 h. The reaction mixture was filtered through a G2 frit, and the filter was washed four times with diethyl ether (160 mL each). The combined organic phases were dried over $MgSO_4$ (96 g) and filtered through a pleated filter. The filter was washed with diethyl ether (160 mL). Diethyl ether was evaporated under reduced pressure. Glycidyl methyl ether (156 g, 1.77 mol, 69%) was obtained as a colorless liquid after fractional distillation (T_b = 72-74 °C (300 mbar)) of the residue. ^1H and ^{13}C NMR spectra can be found in Figure S4 and Figure S5, respectively.

^{1}H NMR (400 MHz, DMSO-*d*$_6$) δ [ppm]: 3.63 (dd, J = 11.4, 2.6 Hz, 1H), 3.28 (s, 3H), 3.16 (dd, J = 11.4, 6.5 Hz, 1H), 3.12 – 3.04 (m, 1H), 2.72 (dd, J = 5.1, 4.2 Hz, 1H), 2.53 (dd, J = 5.1, 2.7 Hz, 1H).

^{13}C NMR (100 MHz, DMSO-*d*$_6$) δ [ppm]: 72.90 (CH_2), 58.27 (CH_3), 50.14 (CH), 43.27 (CH_2).

2.3. Synthesis of 1-methoxy-3-(2-methoxyethoxy)propan-2-ol (MMEPOH)

A flame-dried two-necked Schlenk flask equipped with stirrer bar, septum and reflux condenser was charged with 2-methoxy ethanol (33.6 g, 36.0 mL, 442 mmol) under argon. The flask was immersed in an ice-bath. Sodium (2.84 g, 124 mmol) was added portion wise to the alcohol under stirring and argon. The reaction mixture was allowed to reach room temperature. After complete reaction of the sodium, the solution was heated to 55 °C and GME (9.31 g, 9.50 mL, 106 mmol) was slowly added via syringe pump (1 mL h^{-1}) under argon. The reaction mixture was stirred overnight, cooled to room temperature and neutralized by the addition of aqueous 2 M HCl solution. After evaporation of water and excess 2-methoxy ethanol under reduced pressure, 1-methoxy-3-(2-methoxyethoxy)propan-2-ol (12.03 g, 73.2 mmol, 69%) was obtained after fractional distillation (T_b =60 °C ($5 \cdot 10^{-2}$ mbar)) of the residue. ^1H and ^{13}C NMR spectra can be found in Figure S6 and Figure S7**,** respectively.

^{1}H NMR (400 MHz, DMSO-*d*$_6$) δ [ppm]: 4.79 (d, J = 5.2 Hz, 1H), 3.76 – 3.60 (m, 1H), 3.54 – 3.46 (m, 2H), 3.46 – 3.39 (m, 2H), 3.39 – 3.16 (m, 10H).

^{13}C NMR (100 MHz, DMSO-*d*$_6$) δ [ppm]: 74.26 (CH_2), 72.63 (CH_2), 71.28 (CH_2), 69.92 (CH_2), 68.38 (CH), 58.41 (CH_3), 58.09 (CH_3).

2.4. Synthesis of polyether copolymers (rPEG) and PGME

Caveat: Ethylene oxide is a highly flammable and toxic gas; it must be handled by trained researchers and staff!

The following synthesis protocol was applied for rPEG samples $rPEG_{112}^{0.21}$, $rPEG_{112}^{0.43}$, $rPEG_{120}^{0.45}$ and $rPEG_{110}^{0.55}$. The EO and GME ratio varied depending on the targeted GME content. For PGME, only GME was added via syringe. In the case of $rPEG_{109}^{0.74}$ and PGME, two sequential comonomer addition steps were performed, each with half of the total EO and GME amounts. The second addition was performed after full conversion of both comonomers of the first addition. A sequential monomer addition protocol was applied for polymer compositions exceeding 55mol% GME. For these compositions chain transfer reactions occur when carrying out the polymerization with a single monomer addition. Dividing the overall amount of monomers into two portions ensures a highly controlled polymerization which is a key feature of rPEGs and a prerequisite for medical application.

Potassium *tert*-butoxide (KO*t*Bu) (21.0 mg, 191 µmol) was dissolved in stabilizer-free THF and small quantities of Millipore water and transferred into a flame-dried and argon flushed flask equipped with a teflon stopcock and a septum. MMEPOH (32.0 mg, 195 µmol) was dissolved in benzene and transferred into the flask. High vacuum ($1 \cdot 10^{-3}$ mbar) was applied to the flask and the solvents were removed at 30 °C. The resulting initiator salt was further dried under high vacuum at 60 °C overnight. The initiator salt was dissolved in dry DMSO (10 mL). After freezing the resulting solution at -80 °C, CaH_2-dried glycidyl methyl ether (GME) (979 mg, 1.00 mL, 11.1 mmol) was added to the flask via syringe. Ethylene oxide (EO) (489 mg, 500 µL, 11.1 mmol) was added to the flask via cryo-transfer from a graduated ampoule. The cooling bath was removed, and the reaction mixture was allowed to reach room temperature. The resulting solution was stirred for 1 d at 30 °C under static high vacuum. The flask was ventilated and DMSO was evaporated under reduced pressure. The residue was redissolved in diethyl ether (17 mL) and toluene (2 mL) and acetic acid (34.0 µL) was added under stirring. After 15 min the reaction mixture was filtered through a dense layer of celite. $rPEG_{110}^{0.55}$ (1.44 g, 96%) was obtained as a yellow-colored viscous liquid after evaporation of the solvents and excess acetic acid under reduced pressure. ^{1}H NMR spectra of all synthesized rPEGs can be found in Figure S44 and S55.

^{1}H NMR (D_2O, 400 MHz) δ [ppm]: 3.85-3.45 (m, polyether backbone), 3.36 (s, OCH_3).

2.5. *in situ* ^{1}H NMR kinetics

The following procedure was applied for all kinetics measurements. This exemplary procedure describes the copolymerization of EO with GME in anisole at 55 °C with a degree of deprotonation of 90 %. Measurements in DMSO and toluene were performed using the respective deuterated solvents. Reactivity ratios and reaction rates were determined from *in situ* ^{1}H NMR measurements.

Preparation of the initiator salt solution: In a flame-dried Schlenk flask equipped with a rubber septum and a stir bar, 2-(benzyloxy)ethanol (44.7 mg, 294 µmol, 1 eq.) and KO^{t}Bu (29.7 mg, 0.264 mmol, 0.9 eq.) were dissolved in benzene (8 mL, dried over MS 4 Å). For kinetics utilizing [18]crown-6, a dry benzene solution, using 2 equivalents per potassium cation was added. The mixture was heated under static vacuum for 1 h at 60 °C. The solvents were removed *in vacuo* overnight at 60 °C, yielding the dry, partially deprotonated initiator salt. Subsequently, anisole (2.22 mL, dried over CaH_2) was added. The mixture was heated for 1 h at 60 °C and allowed to cool to room temperature.

Preparation of the polymerization mixture: EO was cryo-transferred into an oven-dried *Norell S-500-VT-7* sealable NMR tube with a Teflon stopcock using an acetone/liquid N_2 bath and static vacuum. Under argon counterflow, glycidyl methyl ether (50.0 µL, 587 µmol, 10 eq., dried twice over CaH_2) and one-fifth of the initiator salt solution was added. The mixture was degassed by applying three freeze-pump-thaw cycles using the acetone/liquid N_2 bath. Then, the NMR tube was transferred into

the preheated NMR device. After the kinetics study, the solvents were removed by applying a nitrogen stream to the solution.

Caveat: In rare cases we experienced breaking of the NMR tube if subjected to liquid nitrogen. We strongly recommend using an acetone/liquid N_2 *bath for cooling. Ethylene oxide is a highly flammable and toxic gas. It must be handled by trained researchers and staff!*

3. Supplementary Text

3.1. Sample description

Throughout this manuscript and the ESI, the samples´ composition is described with

$$\mathrm{rPEG}_{DP}^{f}$$

where f is the molar fraction of GME in the sample and DP the total degree of polymerization. All samples carry a methoxy and hydroxy group at the α- and ω-group, respectively.

3.2. Calculation of incorporated GME content and total degree of polymerization

The calculation of the GME content and total degree of polymerization (DP_{total}) in the rPEG samples is not feasible via end group analysis from the respective ^{1}H NMR spectra because of the overlap of methoxy groups deriving from the initiator and the methoxy methylene side groups. Therefore, the GME content was determined by a combination of MALDI ToF MS and ^{1}H NMR spectroscopy. First, the number average molar mass ($M_{\mathrm{n,MALDI}}$) and the ratio ($I_{\mathrm{backbone}}/I_{\mathrm{MeO}}$) of the integrals of backbone signals (I_{backbone}) from 3.90 to 3.45 ppm to methoxy groups signals (I_{MeO}) from 3.45 to 3.25 ppm was determined via MALDI ToF MS and ^{1}H NMR spectroscopy, respectively. Based on the relationship

$$\left(\frac{I_{\mathrm{backbone}}}{I_{\mathrm{MeO}}}\right) = \frac{5 \cdot DP_{\mathrm{GME}} + 4 \cdot DP_{\mathrm{EO}}}{3 \cdot DP_{\mathrm{GME}} + 3} \qquad \textbf{(S3)}$$

and

$$DP_{\mathrm{total}} = DP_{\mathrm{GME}} + DP_{\mathrm{EO}} = \frac{M_{\mathrm{n,MALDI}} - M_{\mathrm{initiator\ group}} - M_{\mathrm{end\ group}}}{44.05 \cdot \left(\frac{100 - mol\%_{\mathrm{GME}}}{100}\right) + 88.11 \cdot \left(\frac{mol\%_{\mathrm{GME}}}{100}\right)} \qquad \textbf{(S4)}$$

DP_{GME} and therefore the molar content of GME (mol%$_{\mathrm{GME}}$) and DP_{total} of the sample can be determined by choosing values that fit the experimental data. It is important to note that the GME and EO repeating unit of the initiator is included in DP_{GME} and DP_{EO}, respectively. The obtained values can be found in Table S3 and S4.

3.3. Determination of diffusion coefficients and hydrodynamic radii

To determine the diffusion coefficient (D) of the investigated polymer samples, the decay of the polymer backbone signal between 3.85-3.35 ppm in the DOSY NMR (Figure S48 – S52) was integrated and the resulting data points were fitted with a three-parameter fit,

$$I = B + F \cdot e^{-gD} \qquad \textbf{(S5)}$$

wherein I is the intensity of the signal, g is the gradient strength, D is the diffusion coefficient and B and F are fit-dependent values. From the obtained D, the hydrodynamic radii (r_{H}) were calculated utilizing the rearranged Stokes-Einstein equation

$$r_{\mathrm{H}} = \frac{k_B T}{6\pi\eta D} \qquad \textbf{(S6)}$$

wherein k_B is the Boltzmann constant, T the applied temperature and η the viscosity of the solvent at T. Table S7 shows an overview of the obtained D and r_H. The obtained values were compared to the expected values of the PEG fit (Figure S53).

3.4. Determination of end group functionality

To ensure the end group fidelity (ratio of primary- and secondary-hydroxyl end groups) corresponds to the GME faction of the polymer samples, the ^{31}P-IG NMR was applied. The condensation products of 2-chloro-4,4,5,5-tetramethyldioxaphospholane with primary-hydroxyl end groups lead to signals between 147.3 – 147.1 ppm and the secondary-hydroxyl end groups lead to signals between 146.3 – 146.1 ppm.[2] The percentage of secondary-hydroxyl end groups was calculated dividing the integral value of secondary hydroxyl signals by the sum of integral values of primary and secondary hydroxyl signals.

3.5. Determination of retention times and purification of rPEGs and mPEG via HPLC

An analytical reversed-phase (RP) HPLC method (Figure S41) was applied to analyze the dependence of the mPEG and rPEG samples' properties on the interaction with the hydrophobic column material. Herein, the retention time of a sample depends on both the molecular weight and the polarity of the polymer sample. Therefore, it is possible to estimate the polarity of the rPEG samples by comparing their retention times relative to the one of mPEG.[8] In the case of the rPEG samples, a trend is observed. The increase of GME content results in increasing retention times (Fig. S41, Table S5) which can be attributed to both the increase in molecular weight and a decrease in polarity of the rPEG samples. This is in accordance with the observed cloud points of the turbidimetry measurements (Table S6). In similarity to the analytical results, product fractions obtained from the semi-preparative purification depended on the comonomer composition, molecular weight and polarity of the samples (Fig. S43). Repetitions of the purification runs using the same conditions resulted in identical purity profiles and GME contents of the samples (Fig. S46).

3.6. ELISAs of PGME with different α-end groups and selection of initiator

The influence of methoxy methylene groups of PGME on the interaction of end group-selective APAs was assessed via ELISA. For these measurements PGME homopolymers without terminal methoxy groups (BisOH-PGME), with an α-methoxy group followed by two EO repeating units MeO-(EO)$_2$-PGME and with an α-methoxy group followed by three EO repeating units (MeO-(EO)$_3$-PGME) were compared. mPEG (2 kg mol^{-1}) was utilized as a reference. As presented in Fig. S40, the end group-selective APA exhibits no affinity for BisOH-PGME and MeO-(EO)$_2$-PGME in the tested concentration range. Binding is visible for MeO-(EO)$_3$-PGME, and its affinity is 10.8 times lower than for mPEG. Based on these results, MMEPOH was chosen as initiator (Figure 1A) to allow for direct comparison of all rPEG samples and to prevent the formation of an epitope at the α-chain end because of the statistical nature of the monomer incorporation. MMEPOH consists of a methoxy group followed by one EO and one GME repeating unit, resembling the polymer backbone and the isomeric character of rPEG.

3.7. Calculation of EO Sequences in rPEGs

Calculations were performed based on the following fundamental relationships.[9] The copolymerization of two monomers M_1 and M_2 can be divided in four propagation reactions

$$\mathrm{M}_1^* + \mathrm{M}_1 \rightarrow \mathrm{M}_1^*$$

$$\mathrm{M}_1^* + \mathrm{M}_2 \rightarrow \mathrm{M}_2^*$$

$$M_2^* + M_1 \rightarrow M_1^*$$

$$M_2^* + M_2 \rightarrow M_2^*$$

where M_1^* and M_2^* represent the polymer chain end with an active chain end of an ultimate M_1 and M_2 unit, respectively. The consumption rate is described by

$$\frac{d[M_1]}{dt} = k_{11}[M_1^*][M_1] + k_{21}[M_2^*][M_1] \quad \textbf{(S7)}$$

$$\frac{d[M_2]}{dt} = k_{12}[M_1^*][M_2] + k_{22}[M_2^*][M_2] \quad \textbf{(S8)}$$

where k_{11}, k_{21}, k_{12}, k_{22} are the rate constants. Combining the equations with the reactivity ratios r_1 and r_2 which are defined as

$$r_1 = \frac{k_{11}}{k_{12}} \quad \textbf{(S9)}$$

$$r_2 = \frac{k_{22}}{k_{21}} \quad \textbf{(S10)}$$

respectively, one obtains the copolymerization composition equation

$$\frac{d[M_1]}{d[M_2]} = \frac{[M_1](r_1[M_1] + [M_2])}{[M_2]([M_1] + r_2[M_2])} \quad \textbf{(S11)}$$

Based on mole fractions, the copolymerization composition equation can be expressed as

$$F_1 = \frac{r_1 f_1^2 + f_1 f_2}{r_1 f_1^2 + 2 f_1 f_2 + r_2 f_2^2} \quad \textbf{(S12)}$$

or

$$\frac{F_1}{F_2} = \frac{f_1(r_1 f_1 + f_2)}{f_2(r_2 f_2 + f_1)} \quad \textbf{(S13)}$$

where f_1 and f_2 are the mole fractions of monomers M_1 and M_2 in the feed, respectively, and F_1 and F_2 are the mole fractions of M_1 and M_2 in the copolymer, respectively. In the case of a random copolymerization ($r_1 = r_2 = 1$) no composition drift is obtained because $f_1 = F_1$ and $f_2 = F_2$ over the whole range of the copolymerization. This allows a simplified calculation of the mole fraction of a sequence of M_1 units $N_{1,x}$ over the whole polymer chain with

$$N_{1,x} = (p_{11})^{(x-1)} p_{12} \quad \textbf{(S14)}$$

Where p_{11} and p_{12} are the probability of forming a M_1M_1 and M_1M_2 dyad, respectively, and x is the sequence length from 1 to infinity. p_{11} and p_{12} are defined as

$$p_{11} = \frac{r_1}{r_1 + ([\mathrm{M}_2]/[\mathrm{M}_1])} \quad \textbf{(S15)}$$

$$p_{12} = \frac{[\mathrm{M}_2]}{r_1[\mathrm{M}_1] + [\mathrm{M}_2]} \quad \textbf{(S16)}$$

3.8. Analysis of microstructure based on simulations

To provide an insight into chemical composition distribution, more precisely the consecutive EO repeating units in the rPEGs, simulations were performed. Hence, four polymerizations were conducted *in silico*. For this purpose, the rationale of a living anionic polymerization was taken as a foundation which implies the absence of chain termination or chain transfer reactions and a Poisson distribution of the chain lengths. The copolymerization reaction can be described by the four rate constants: The homo-propagation rate constants k_{11} and k_{22} as well as the cross-propagation rate constants k_{12} and k_{21}. The copolymerization kinetics was assumed to be perfectly random, implying

$$k_{11} = k_{22} = k_{12} = k_{21} \quad \textbf{(S17)}$$

As a result, it was assumed in the simulations that EO and GME monomers possess the same probability to be incorporated into the chain. For each polymerization, 10^4 chains were simulated. The simulations were run with a targeted quantitative conversion and an overall degree of polymerization of 114. Molar ratios of 20 up to 50% of GME with a stepwise increase by 10 mol% were presumed. In Table S8 the four simulated polymerizations and the respective proportional occurrence of 16 consecutive EO units are listed. To visualize the results of the simulations, 15 exemplary chains for each respective GME content are shown in Figure 6, Figures S62 – S64.

3.9. Calculation of possible GME unit distributions in individual chains

The number of possible combinations (P) of 23 GME units (k) in a polymer with a total chain length (n) of 114 repeating units can be calculated by

$$P = \binom{n}{k} = \frac{n!}{k!\,(n-k)!} = \frac{114!}{23!\,(114-23)!} = 7.3 \cdot 10^{23} \quad \textbf{(S18)}$$

3.10. *in situ* ^{1}H NMR kinetics

Rate constants of EO and GME: We investigated the copolymerization of EO and GME in the solvents DMSO-d_6, toluene-d_8, and anisole by *in-situ* ^{1}H NMR kinetics studies. EO and GME were copolymerized in the respective solvents in an NMR tube with a sealable Teflon stopcock. Copolymerizations were conducted with the potassium salt of 2-(benzyloxy)ethanol at two different degrees of deprotonation (*dod*) 50% and 90%.

$$dod = \frac{\text{base eq.}}{\text{hydroxyl group}} \cdot 100\% \quad \textbf{(S19)}$$

The *dod* denotes how much base equivalent was added per hydroxyl group of the initiator alcohol to prepare the initiator salt. From the slope of the pseudo-first-order plots of the copolymerization (Figure S8 – S13) obtained from the monomer integrals in the ^{1}H NMR spectra, the resulting apparent rate constant of EO ($k_{\mathrm{app,EO}}$) was determined based on equation S29. This allows for comparison of the reaction rate in different solvents at a constant initiator concentration. Copolymerization in DMSO showed a linear behavior in the pseudo-first-order plots (Figure S8). In anisole, a slight induction period was present at the beginning of the copolymerization with both *dod*s (Figure S9,

enlargement in Figure S10). In toluene (Figure S11), the induction period is only observed at a *dod* of 50% (enlargement in Figure. S12). At the beginning of the copolymerization, the reaction rate increases, which can be explained by the dissolution of aggregates, leading to more reactive active species in the copolymerization system. The slope increases slowly after the initial period, comparable to the copolymerization in anisole. A pronounced induction period was observed in DMSO (*dod*: 50%, Figure S13) when a bifunctional initiator was utilized. This induction period slows down the overall reaction due to the relatively slow reaction rate at the beginning of the copolymerization. After the induction period, the reaction rate was comparable to the monofunctional initiator conditions (Figures S8 and S13). We want to emphasize that the reaction rates of EO in the copolymerization cannot be treated as the homopolymerization reaction rates. Determination of the propagation constant k_p ($[k_p]$ = L mol^{-1} s^{-1}) is not possible, as the aggregation number is unknown. The reaction rate is slightly influenced by a change of *dod* from 50% to 90% in DMSO. Polymerization in anisole occurs twice as fast as in toluene at a *dod* of 50%. However, at a *dod* of 90%, $k_{app,EO}$ in toluene is ≈1.8 times higher than in anisole. Addition of [18]crown-6 further increases the reaction rate by a factor of ≈1.9 compared to the run without [18]crown-6.

Reactivity ratios of EO and GME: Determination of the reactivity ratios (r) can be performed by various methods, such as differential and integral methods. Herein, ideal models ($r_1 \cdot r_2 = 1$) which are chain end independent (such as BSL, Jaacks, Ideal Integrated) should be applied if the models adequately describe the data.[10,11] The Jaacks method[12] is given by the equation

$$\log\left(\frac{[\mathrm{M}_1]_t}{[\mathrm{M}_1]_0}\right) = r_1 \cdot \log\left(\frac{[\mathrm{M}_2]_t}{[\mathrm{M}_2]_0}\right) \qquad \textbf{(S20)}$$

We decided to present the results using the Jaacks method as it is comprehensible and can be readily recalculated. By plotting $\log([\mathrm{M}_1]_t/[\mathrm{M}_1]_0)$ versus $\log([\mathrm{M}_2]_t/[\mathrm{M}_2]_0)$, r_1 can be derived from the slope. With the relation $r_1 \cdot r_2 = 1$, both reactivity ratios can be calculated.
Reactivity ratios of the EO/GME comonomer pair were investigated at two different degrees of deprotonation, using the monofunctional 2-(benzyloxy)ethanol (BnO) or 3-ethoxypropane-1,2-diol (EPD). The summarized results can be found in Table S2. The results were obtained using the NIREVAL software.[1] Figure S15 shows the reactivity ratios in dependence on the *dod*. In DMSO, they do not change significantly. Furthermore, the results indicate a fully random copolymer, as both reactivity ratios are almost one. In anisole, and especially in toluene, the reactivity ratios diverge significantly. This divergence becomes slightly more pronounced when the degree of deprotonation is increased. The difference in reactivity ratios directly affects the polymer composition. While the resulting difference may seem small, Figure. S16 demonstrates that polymerization in toluene results in a slight gradient microstructure. In contrast, copolymerization in DMSO yields an almost ideal random microstructure. To determine if the reactivity ratios from the DMSO experiment can be applied to toluene, [18]crown-6 was added to reduce the chelation of GME to the potassium counterion. The reaction condition with a *dod* of 90% in toluene was chosen because of the highest difference in reactivity ratios. Unexpectedly, the reactivity ratios converged just slightly (Figure S15, red squares). This finding suggests that the role of the solvent in the copolymerization in DMSO is not yet fully understood. The determined reactivity ratios can be found in Table S2 and the resulting GPC curves in Figure S32 to Figure S39. The use of EPD as a bifunctional initiator yielded the same reactivity ratios as BnO (Table S2, entry 8). The copolymer composition was simulated for all experiments and is shown in Figures S25 to S31. In summary, the reaction rates in apolar solvents like toluene and anisole, although slower overall, demonstrate that such solvents remain viable alternatives to DMSO, particularly when specific circumstances make DMSO unsuitable. The faster copolymerization in toluene compared to anisole further underscores the importance of solvent choice. Our results also reveal that increasing the *dod* from 50% to 90% with a monofunctional

initiator significantly accelerates copolymerization rates, except in DMSO, where a minor influence is observed.

Derivation of the pseudo-first-order plot:

$$-\frac{\mathrm{d[M]}}{\mathrm{d}t} = k_\mathrm{p} * [\mathrm{I}] * [\mathrm{M}] \tag{S21}$$

$$k_\mathrm{app} = k_\mathrm{p} * [\mathrm{I}] \tag{S22}$$

$$-\frac{\mathrm{d[M]}}{\mathrm{d}t} = k_\mathrm{app}[\mathrm{M}] \tag{S23}$$

$$-\mathrm{d[M]}\frac{1}{[\mathrm{M}]} = k_\mathrm{app}\,\mathrm{d}t \tag{S24}$$

$$-\int_{[\mathrm{M}]_0}^{[\mathrm{M}]_t} \frac{1}{[\mathrm{M}]}\mathrm{d[M]} = k_\mathrm{app}\int_0^t \mathrm{d}t \tag{S25}$$

$$\int \frac{1}{x} = \ln{(x)} \tag{S26}$$

$$-(\ln([\mathrm{M}]_t) - \ln([\mathrm{M}]_0)) = k_\mathrm{app} * t \tag{S27}$$

$$-\ln\left(\frac{[\mathrm{M}]_t}{[\mathrm{M}]_0}\right) = k_\mathrm{app} * t \tag{S28}$$

$$\ln\left(\frac{[\mathrm{M}]_0}{[\mathrm{M}]_t}\right) = k_\mathrm{app} * t \tag{S29}$$

The initiator concentration [I] consists of deprotonated and protonated initiator species and assuming all initiator reacts, this equals the concentration of the growing polymer chain ends. It is important to note, that the apparent rate constant k_app cannot be converted to k_p by the relation $k_\mathrm{p} = \frac{k_\mathrm{app}}{[\mathrm{I}]}$, if the degree of association (or aggregation) is unknown.[13,14] If $\ln\left(\frac{[\mathrm{M}]_0}{[\mathrm{M}]_t}\right)$ is plotted versus t, the slope yields k_app.

4. Scheme S1 to S2

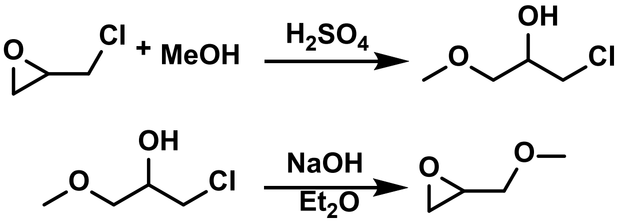

Scheme S1. Synthesis of glycidyl methyl ether.

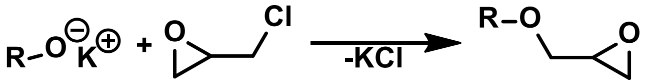

Scheme S2. Occurring chain termination reaction in the presence of epichlorohydrin in the polymerization mixture.

5. Figs. S1 to S74

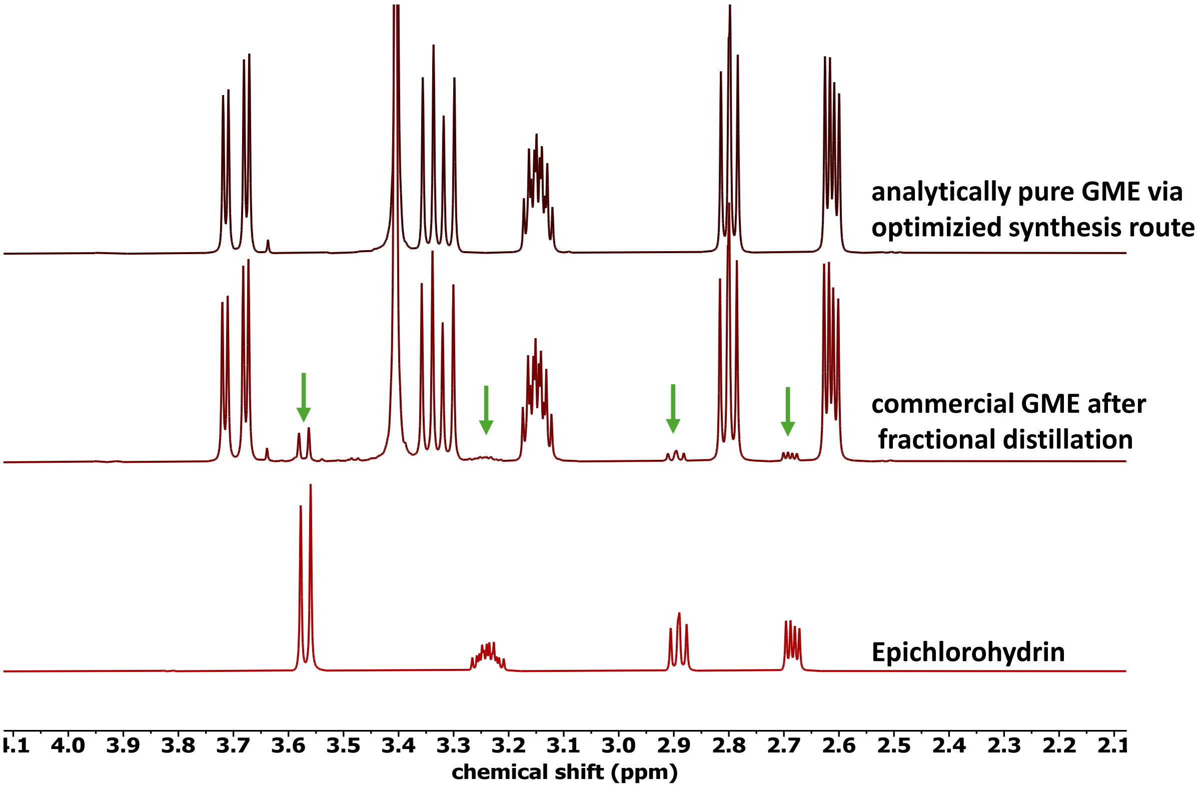

Figure S1. Stacked ^{1}H NMR spectra ($CDCl_3$, 400 MHz) of synthesized GME (top), commercial GME after fractional distillation (middle) and epichlorohydrin (bottom); traces of non-removable epichlorohydrin impurities are indicated via green arrows.

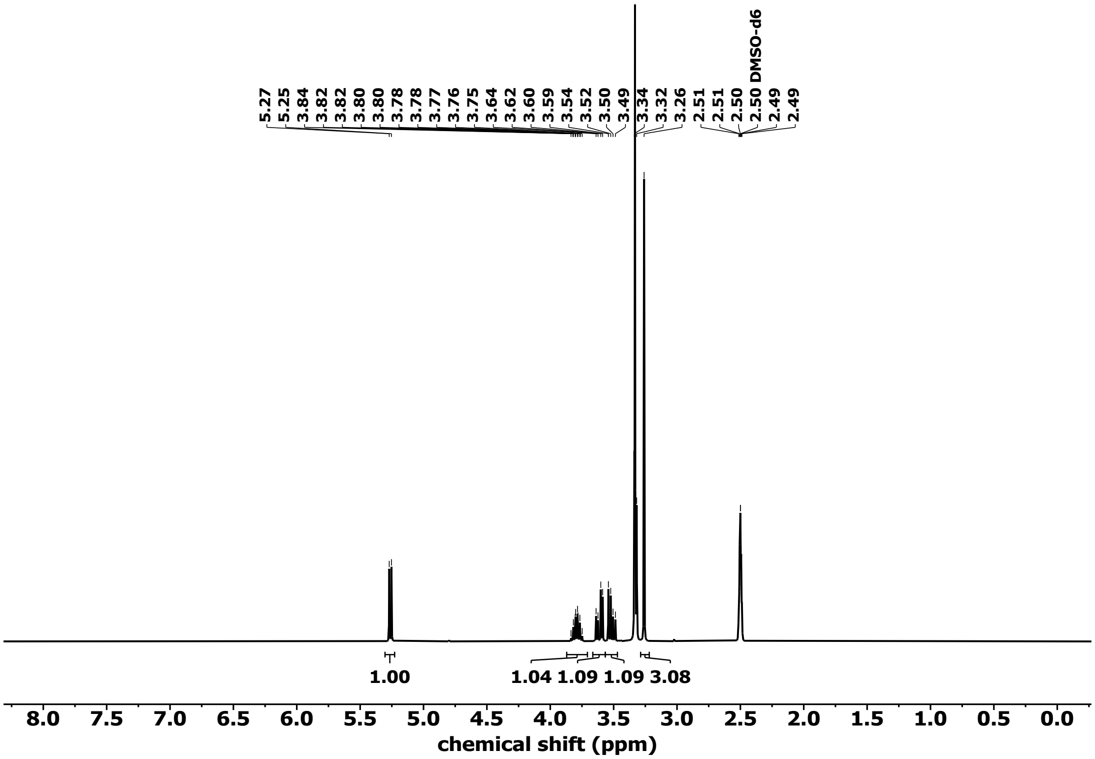

Figure S2. ^{1}H NMR spectrum (DMSO-d_6, 400 MHz) of 1-chloro-3-methoxy-propan-2-ol; Integration of signal at 3.33 ppm (dd) is not possible because of resonance from water traces in DMSO-d_6.

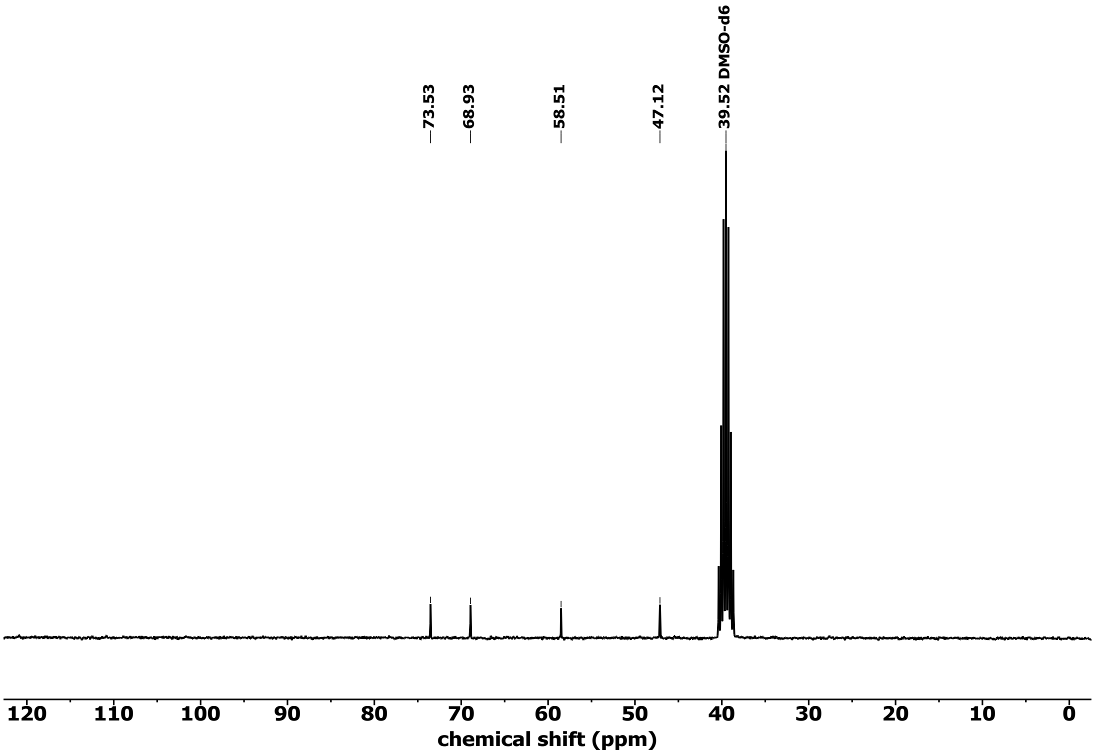

Figure S3. ^{13}C NMR spectrum (DMSO-d_6, 100 MHz) of 1-chloro-3-methoxy-propan-2-ol.

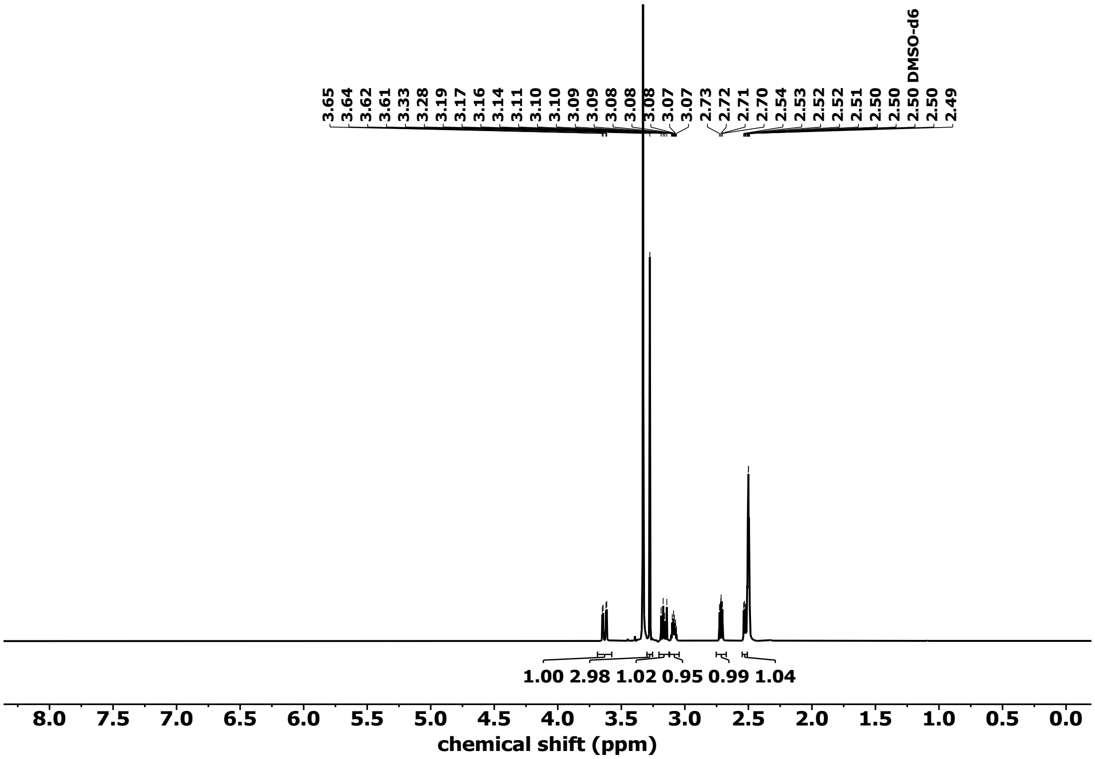

Figure S4. ^{1}H NMR spectrum (DMSO-d_6, 400 MHz) of glycidyl methyl ether.

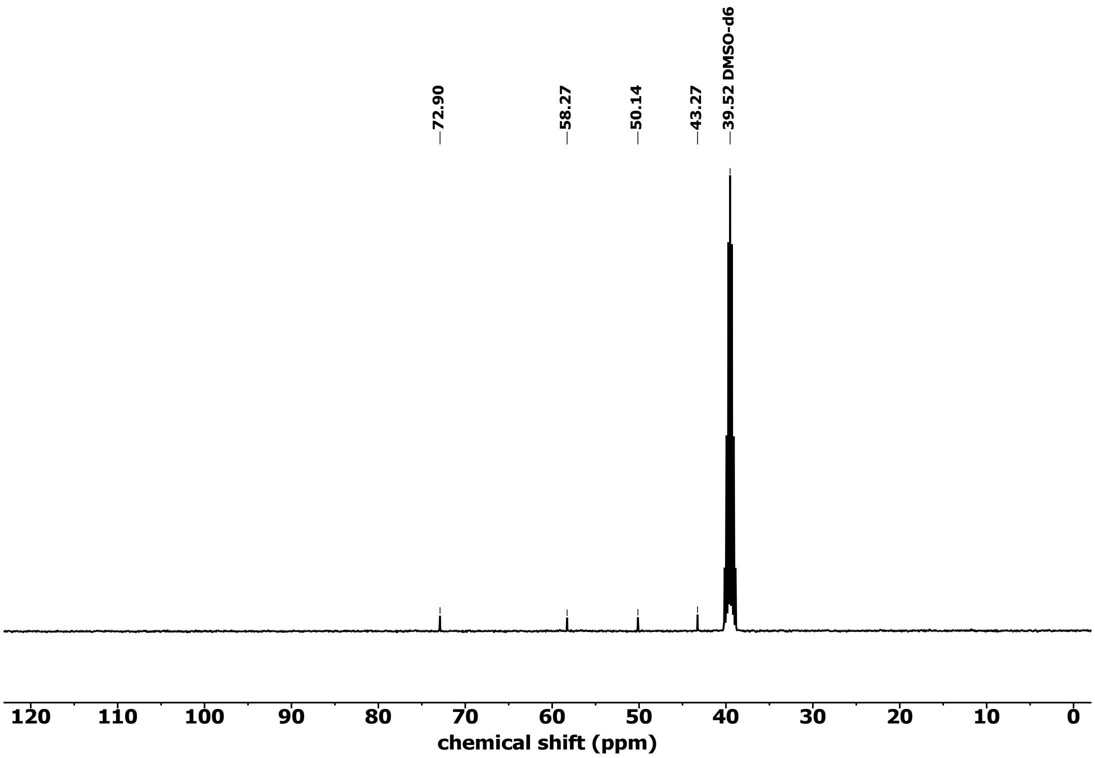

Figure S5. ^{13}C NMR spectrum (DMSO-d_6, 100 MHz) of glycidyl methyl ether.

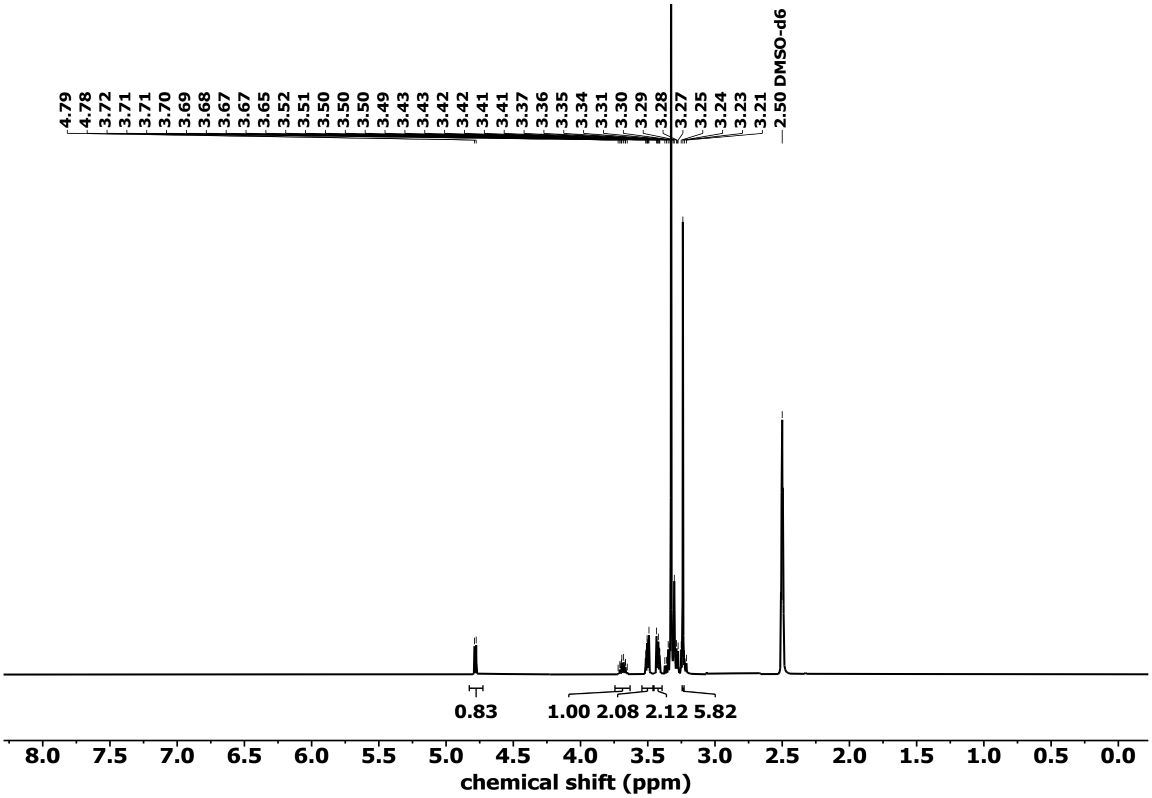

Figure S6. ^{1}H NMR spectrum (DMSO-d_6, 400 MHz) of 1-methoxy-3-(2-methoxyethoxy)propan-2-ol; Integration of signal at 3.38-3.26 ppm (m) is not possible because of resonance from water traces (3.33 ppm) in DMSO-d_6.

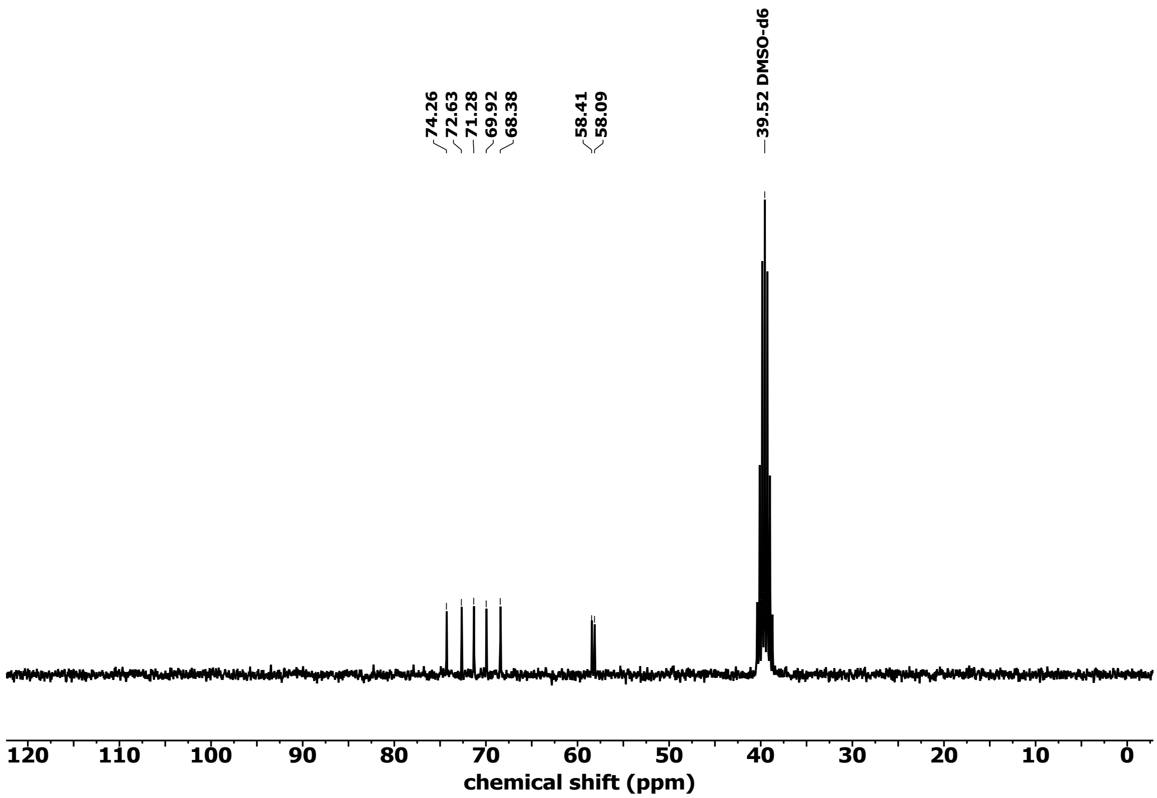

Figure S7. ^{13}C NMR spectrum (DMSO-d_6, 100 MHz) of 1-methoxy-3-(2-methoxyethoxy)propan-2-ol.

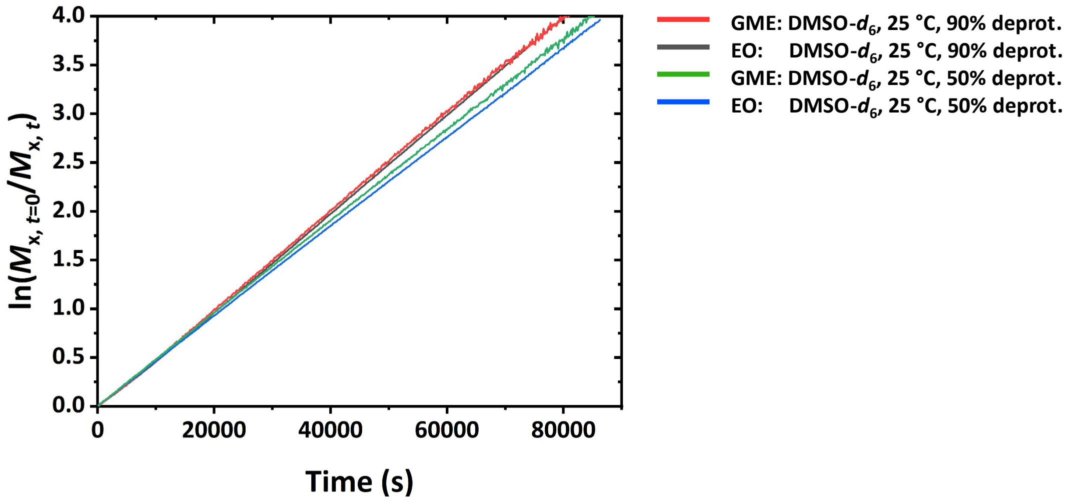

Figure S8. Pseudo-first-order plot of the copolymerization of EO with GME from **Table S1**. Green and blue: entry 1. Red and black: entry 2.

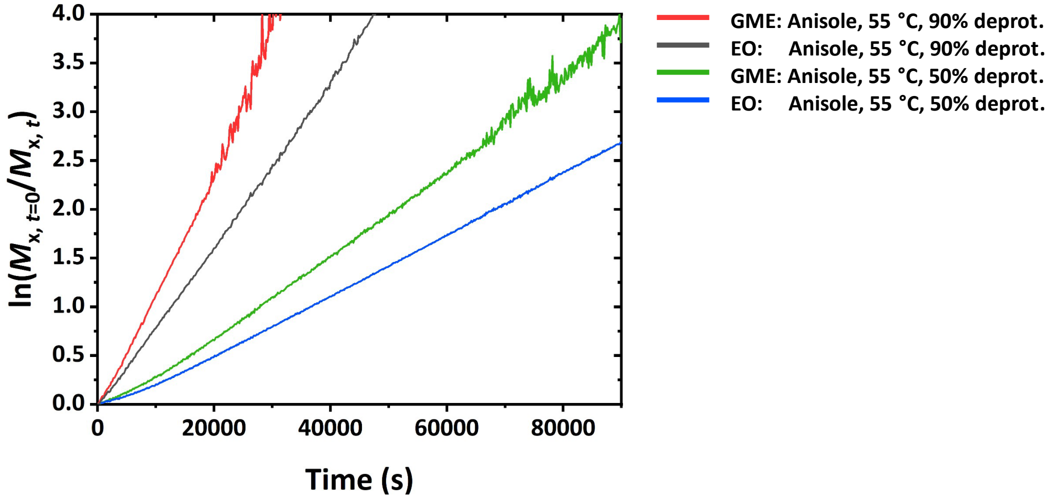

Figure S9. Pseudo-first-order plot of the copolymerization of EO with GME from **Table S1**. Green and blue: entry 3. Red and black: entry 4.

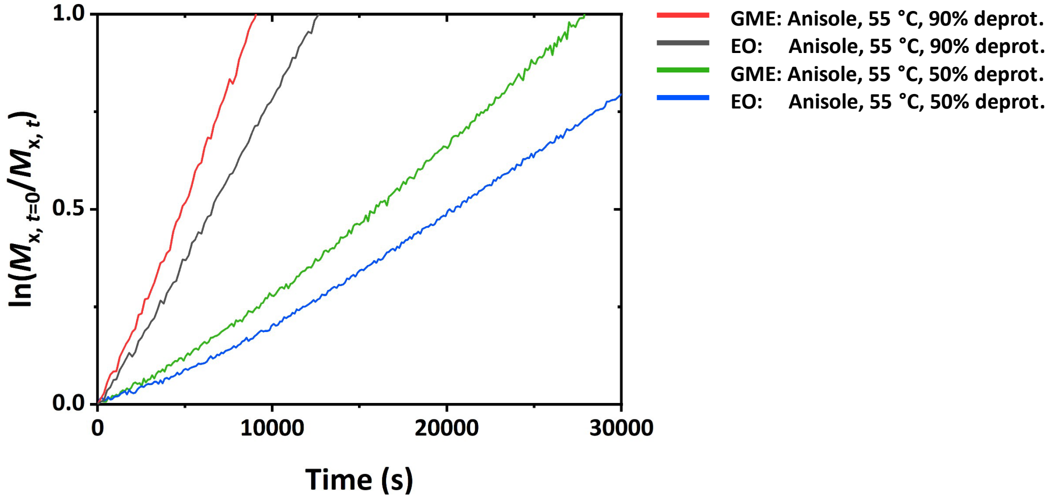

Figure S10. Enlargement of the pseudo-first-order plot of the copolymerization of EO with GME from **Table S1**. Green and blue: entry 3. Red and black: entry 4. The graphs follow nonlinear behavior at the beginning of the copolymerization, indicating an induction period.

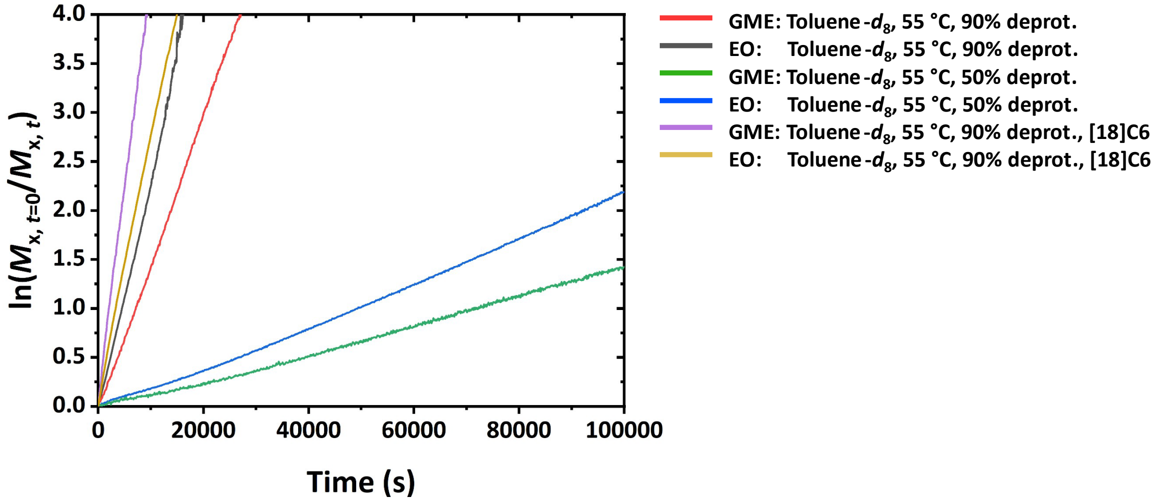

Figure S11. Pseudo-first-order plot of the copolymerization of EO with GME from **Table S1**. Green and blue: entry 5. Red and black: entry 6. Purple and yellow: entry 7.

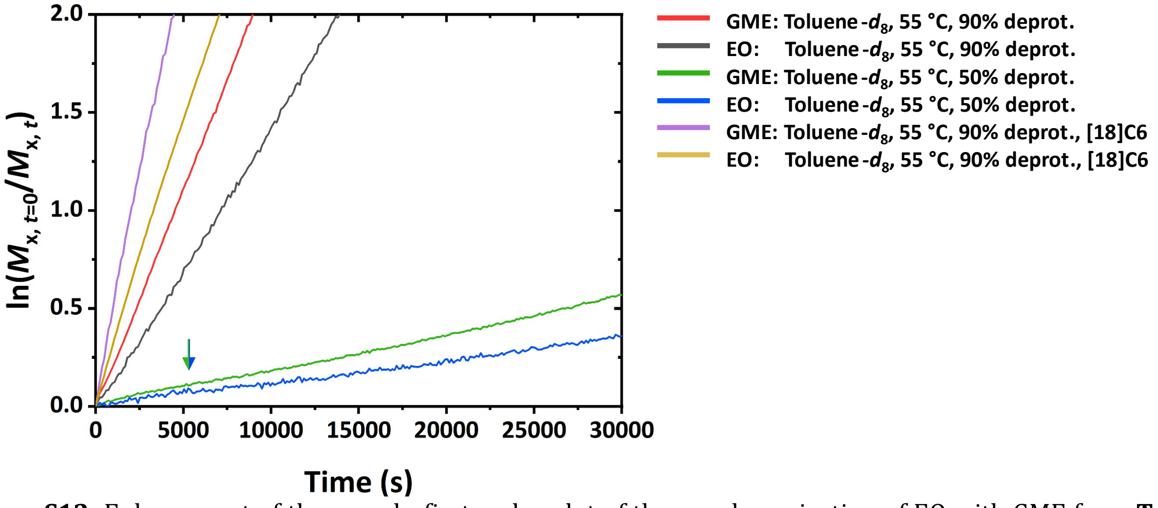

Figure S12: Enlargement of the pseudo-first-order plot of the copolymerization of EO with GME from **Table S1**. Green and blue: entry 5. Red and black: entry 6. Purple and yellow: entry 7. Green-blue arrow marks the end of the time range with an increased reaction rate due to the dissolution of aggregates and/or overheating.

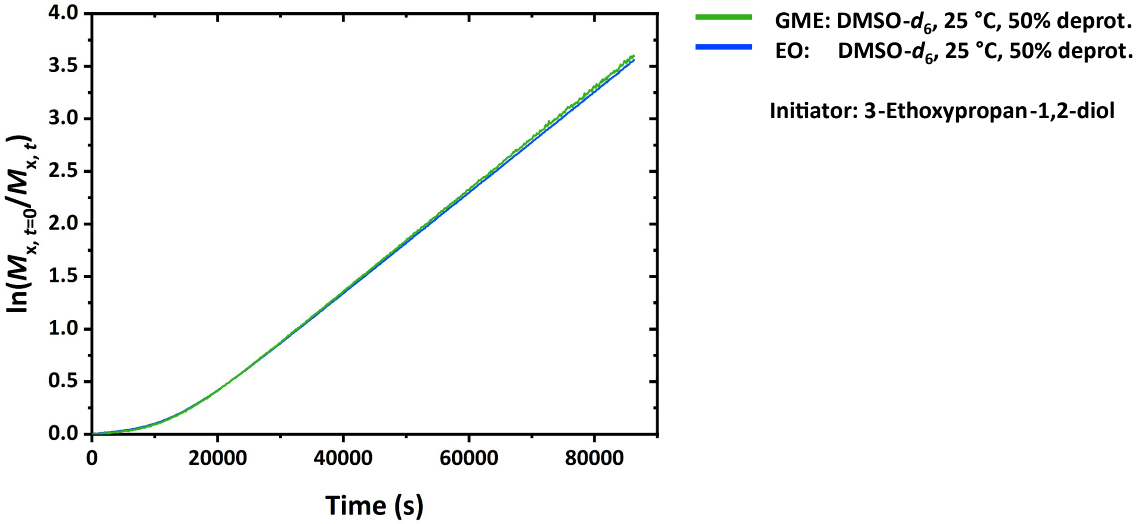

Figure S13: Pseudo-first-order plot of the copolymerization of EO with GME from **Table S1**, entry 8. Active chain end concentration: $3.60\cdot10^{-2}$ mol/L.

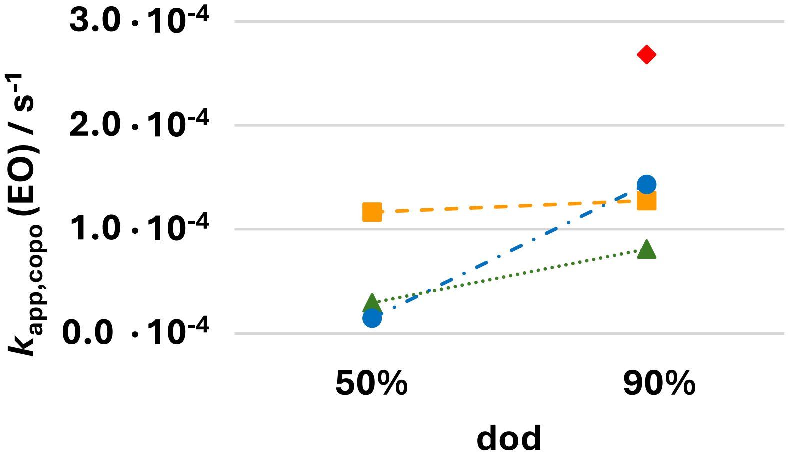

Figure S14. Apparent reaction rates of EO in the copolymerization with GME. The values for experiments in DMSO were adjusted to fit the initiator concentrations of the anisole and toluene experiments. *dod*: degree of deprotonation. Orange squares ■: DMSO, 25 °C. Green triangles ▲: anisole, 55 °C. Blue circles ●: toluene, 55 °C. Red upside square ◆: toluene, 55 °C, [18]crown-6 addition. The values are listed in **Table S1**.

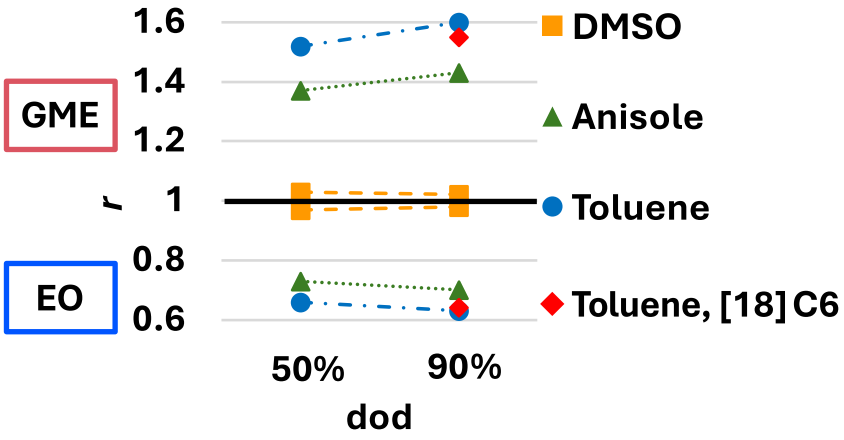

Figure S15. Reactivity ratios of the AROP of EO/PO. DMSO experiments were conducted at 25 °C, anisole and toluene experiments at 55 °C. *dod*: degree of deprotonation. The values can be found in **Table S2**.

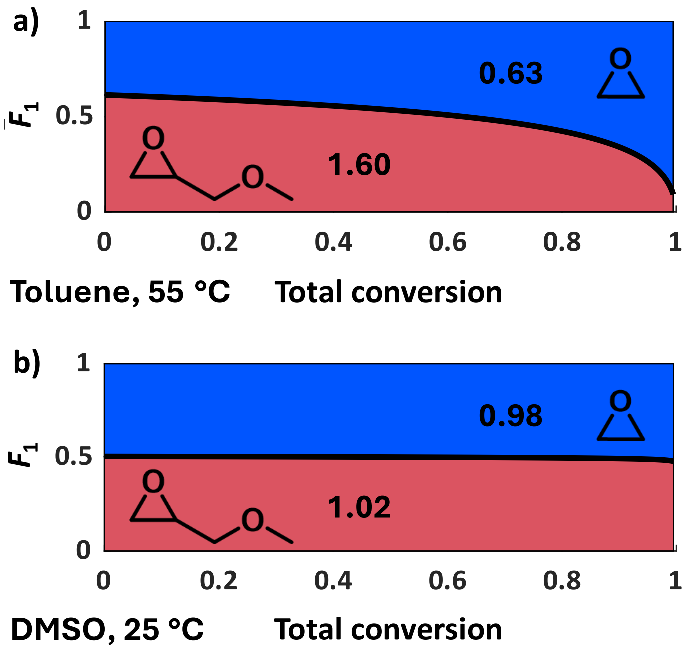

Figure S16. Simulated polymer composition at the denoted polymerization conditions with an equimolar monomer feed and a degree of deprotonation of 90%.

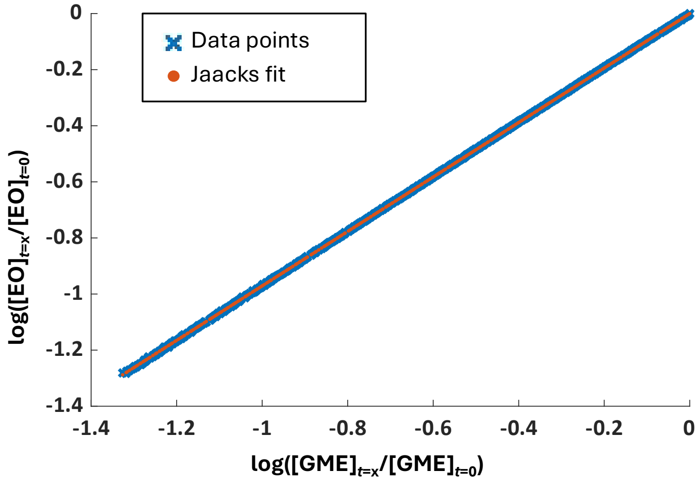

Figure S17. Jaacks fit of the copolymerization of EO and GME from **Table S2**, entry 1.

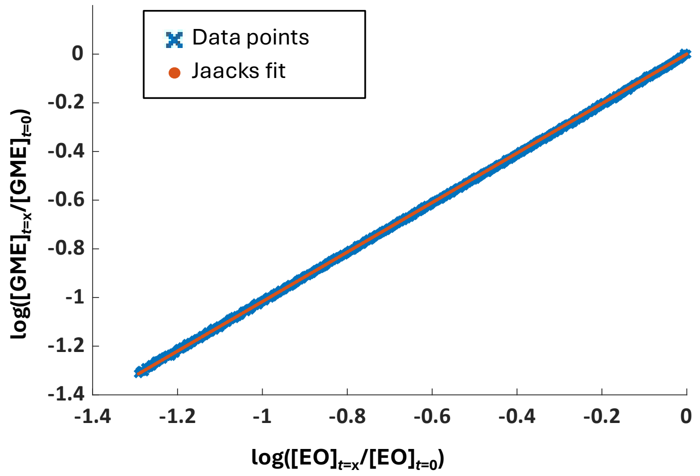

Figure S18. Jaacks fit of the copolymerization of EO and GME from **Table S2**, entry 2.

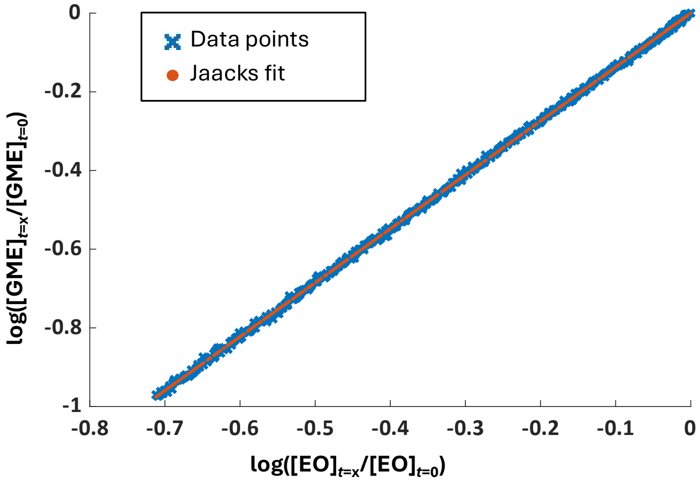

Figure S19. Jaacks fit of the copolymerization of EO and GME from **Table S2**, entry 3. Fit was applied over the whole copolymerization until a conversion of 85%, due to a declining signal-to-noise ratio.

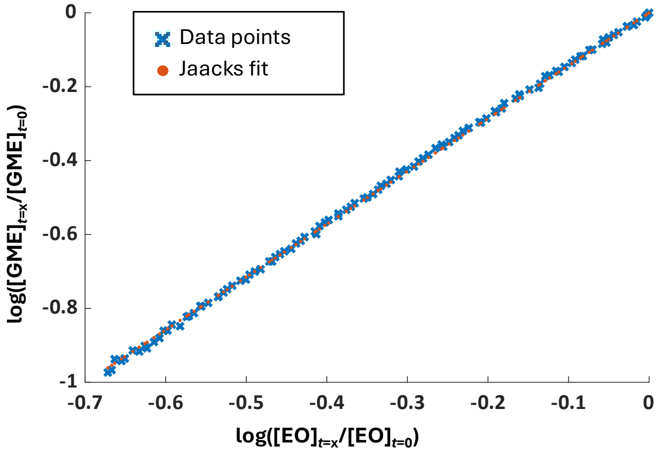

Figure S20. Jaacks fit of the copolymerization of EO and GME from **Table S2**, entry 4. Fit was applied over the whole copolymerization until a conversion of 84%, due to a declining signal-to-noise ratio.

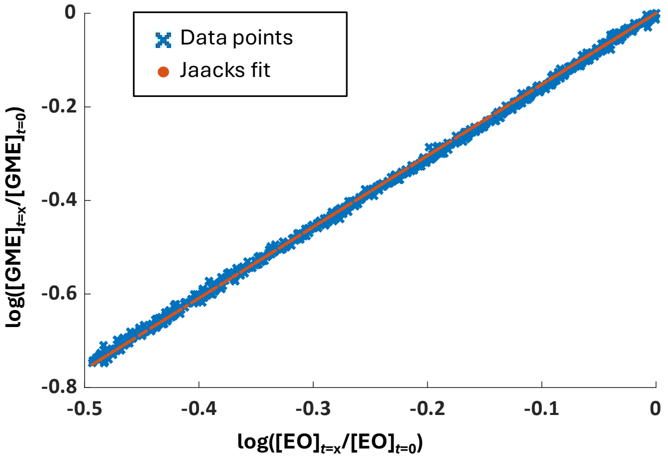

Figure S21. Jaacks fit of the copolymerization of EO and GME from **Table S2**, entry 5. Fit was applied over the whole copolymerization until a conversion of 75%, due to a declining signal-to-noise ratio.

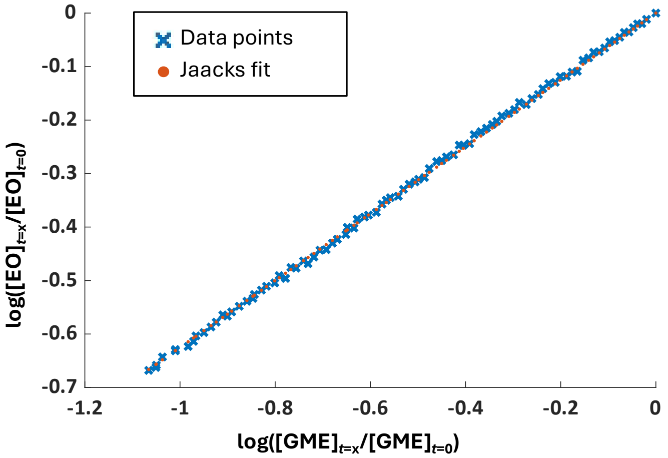

Figure S22. Jaacks fit of the copolymerization of EO and GME from **Table S2**, entry 6. Fit was applied over the whole copolymerization until a conversion of 85%, due to a declining signal-to-noise ratio.

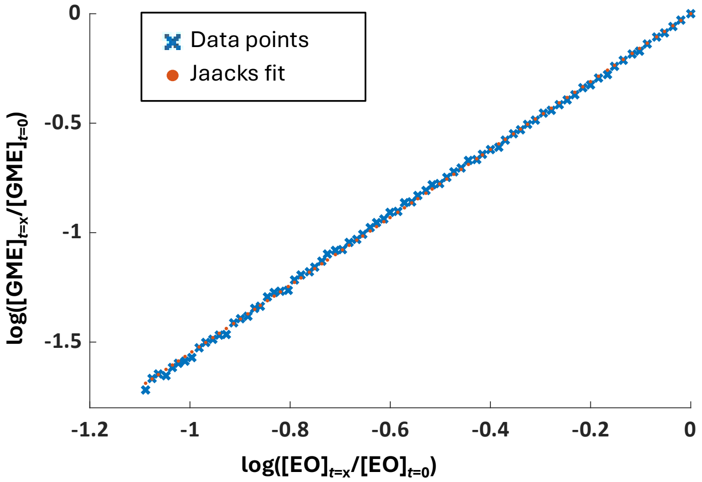

Figure S23. Jaacks fit of the copolymerization of EO and GME from **Table S2**, entry 7.

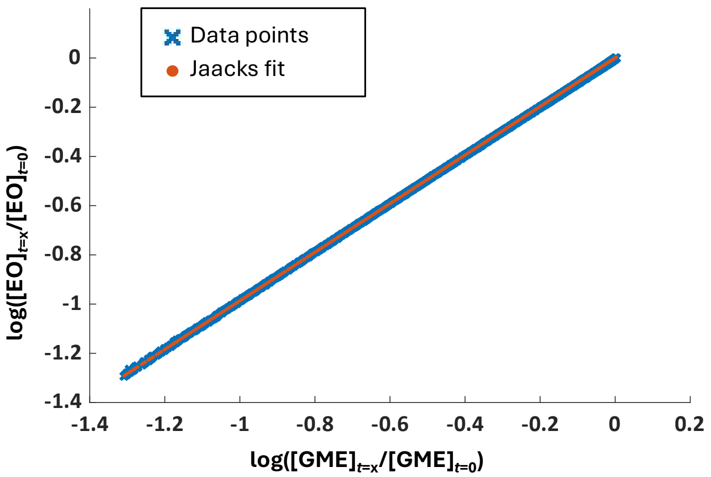

Figure S24. Jaacks fit of the copolymerization of EO and GME from **Table S2**, entry 8. Fit was applied over the whole copolymerization.

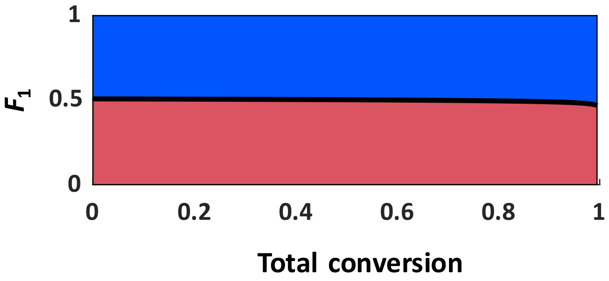

Figure S25. Composition plot of the *in situ* ^{1}H NMR copolymerization kinetics study of EO (blue) with GME (red) with an equimolar monomer ratio (Solvent: DMSO-d_6, 25 °C, *dod*: 50%) with *r*(GME) = 1.03, *r*(EO) = 0.97.

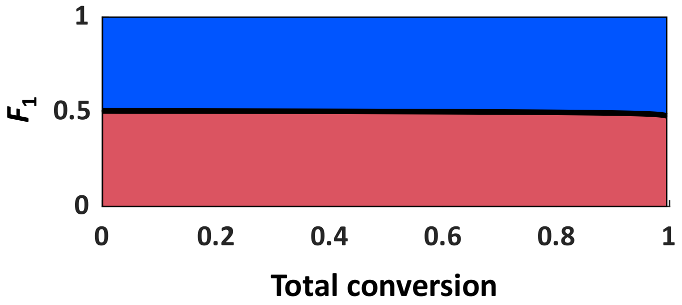

Figure S26. Composition plot of the *in situ* ^{1}H NMR copolymerization kinetics study of EO (blue) with GME (red) with an equimolar monomer ratio (Solvent: DMSO-d_6, 25 °C, *dod*: 90%) with *r*(GME) = 1.02, *r*(EO) = 0.98.

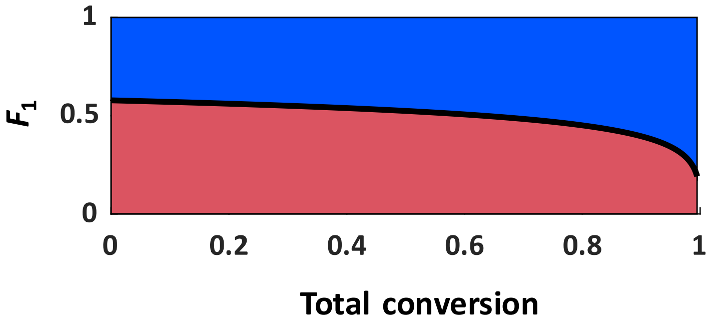

Figure S27. Composition plot of the *in situ* ^{1}H NMR copolymerization kinetics study of EO (blue) with GME (red) with an equimolar monomer ratio (Solvent: anisole, 55 °C, *dod*: 50%) with *r*(GME) = 1.37, *r*(EO) = 0.73.

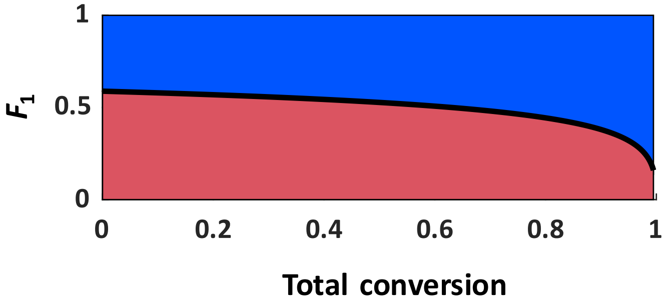

Figure S28. Composition plot of the *in situ* ^{1}H NMR copolymerization kinetics study of EO (blue) with GME (red) with an equimolar monomer ratio (Solvent: anisole, 55 °C, *dod*: 90%) with *r*(GME) = 1.43, *r*(EO) = 0.70.

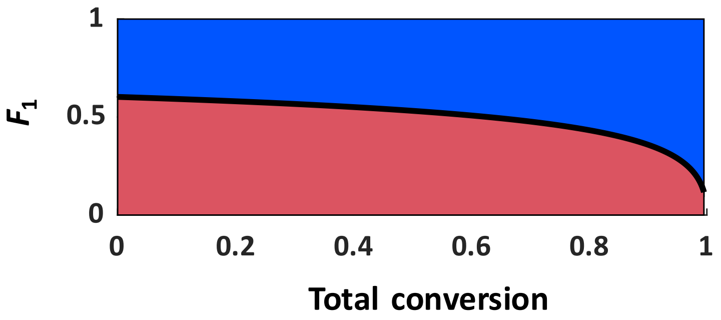

Figure S29. Composition plot of the *in situ* ^{1}H NMR copolymerization kinetics study of EO (blue) with GME (red) with an equimolar monomer ratio (Solvent: toluene-d_8, 55 °C, *dod*: 50%) with *r*(GME) = 1.52, *r*(EO) = 0.66.

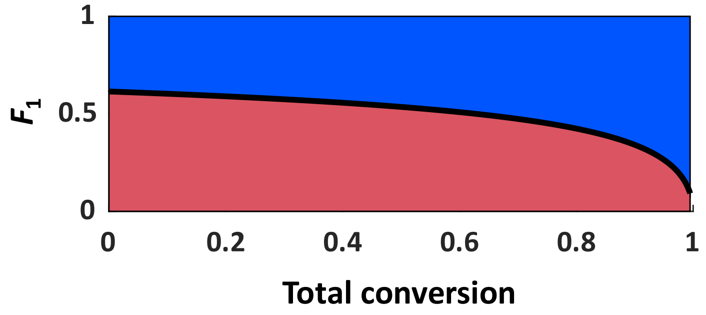

Figure S30. Composition plot of the *in situ* ^{1}H NMR copolymerization kinetics study of EO (blue) with GME (red) with an equimolar monomer ratio (Solvent: toluene-d_8, 55 °C, *dod*: 90%) with *r*(GME) = 1.60, *r*(EO) = 0.63.

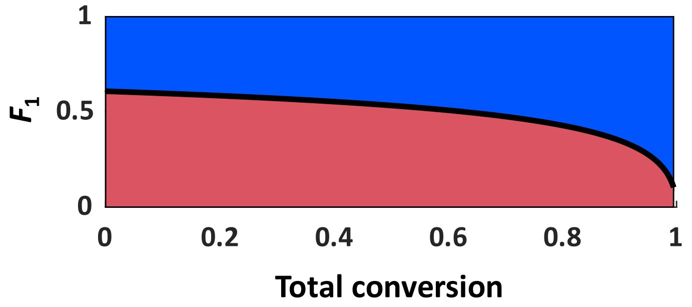

Figure S31. Composition plot of the *in situ* ^{1}H NMR copolymerization kinetics study of EO (blue) with GME (red) with an equimolar monomer ratio (Solvent: toluene-d_8, 55 °C, *dod*: 90%) with *r*(GME) = 1.55, *r*(EO) = 0.64.

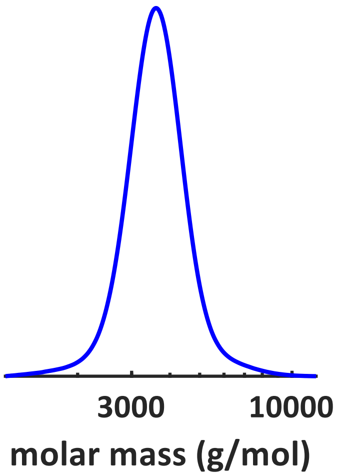

Figure S32. GPC elugram of the polymer obtained in the NMR tube after ^{1}H NMR kinetics measurements. Initiator: 2-(benzyloxy)ethanol, solvent: DMSO-d_6, degree of deprotonation: 50%, temperature: 25 °C.

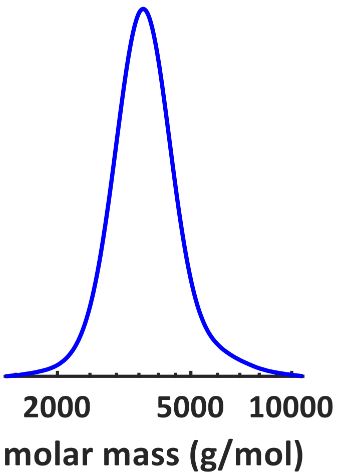

Figure S33. GPC elugram of the polymer obtained in NMR tube after ^{1}H NMR kinetics. Initiator: 2-(benzyloxy)ethanol, solvent: DMSO-d_6, degree of deprotonation: 90%, temperature: 25 °C.

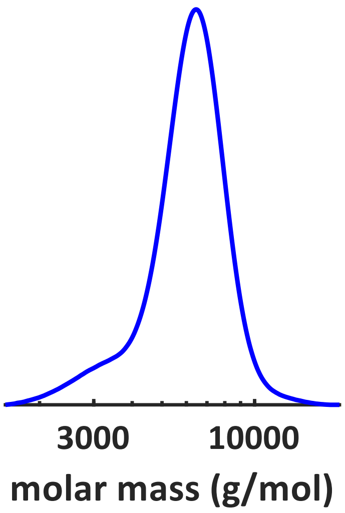

Figure S34. GPC elugram of the polymer obtained in NMR tube after ^{1}H NMR kinetics. Initiator: 3-ethoxypropane-1,2-diol, solvent: DMSO-d_6, degree of deprotonation: 50%, temperature: 25 °C.

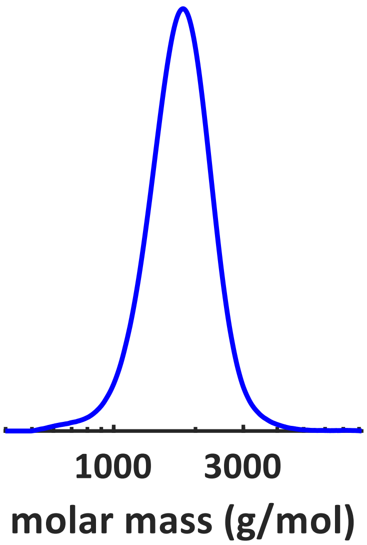

Figure S35. GPC elugram of the polymer obtained in NMR tube after ^{1}H NMR kinetics. Initiator: 2-(benzyloxy)ethanol, solvent: anisole, degree of deprotonation: 50%, temperature: 55 °C.

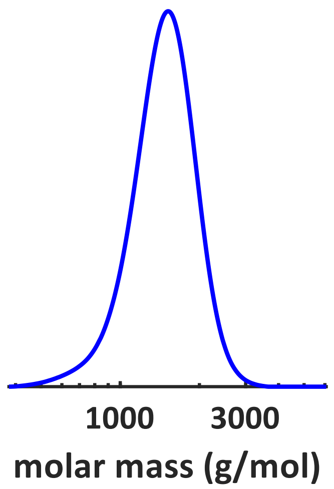

Figure S36. GPC elugram of the polymer obtained in NMR tube after ^{1}H NMR kinetics. Initiator: 2-(benzyloxy)ethanol, solvent: anisole, degree of deprotonation: 90%, temperature: 55 °C.

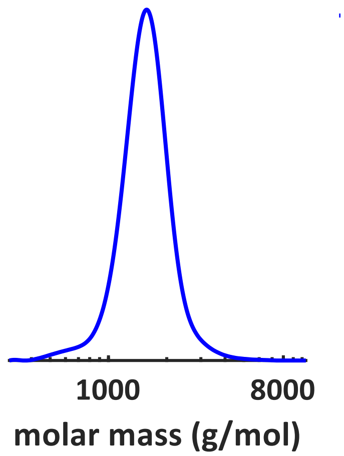

Figure S37. GPC elugram of the polymer obtained after ^{1}H NMR kinetics. Initiator: 2-(benzyloxy)ethanol, solvent: toluene-d_8, degree of deprotonation: 50%, temperature: 55 °C.

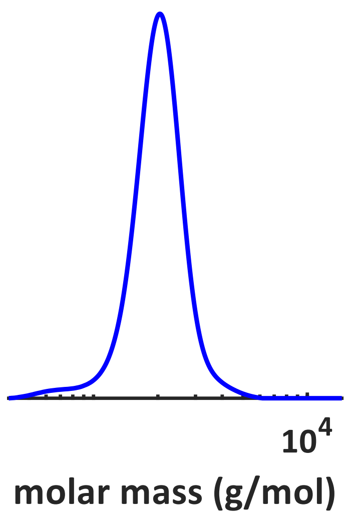

Figure S38. GPC elugram of the polymer obtained after ^{1}H NMR kinetics. Initiator: 2-(benzyloxy)ethanol, solvent: toluene-d_8, degree of deprotonation: 90%, temperature: 55 °C.

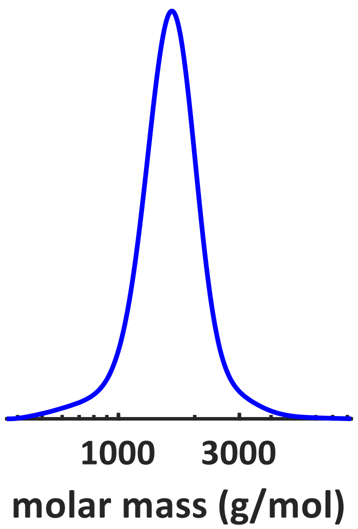

Figure S39. GPC elugram of the polymer obtained after ^{1}H NMR kinetics. Initiator: 2-(benzyloxy)ethanol, solvent: toluene-d_8, degree of deprotonation: 90%, temperature 55 °C, addition of 2 eq. of [18]crown-6 per potassium equivalent.

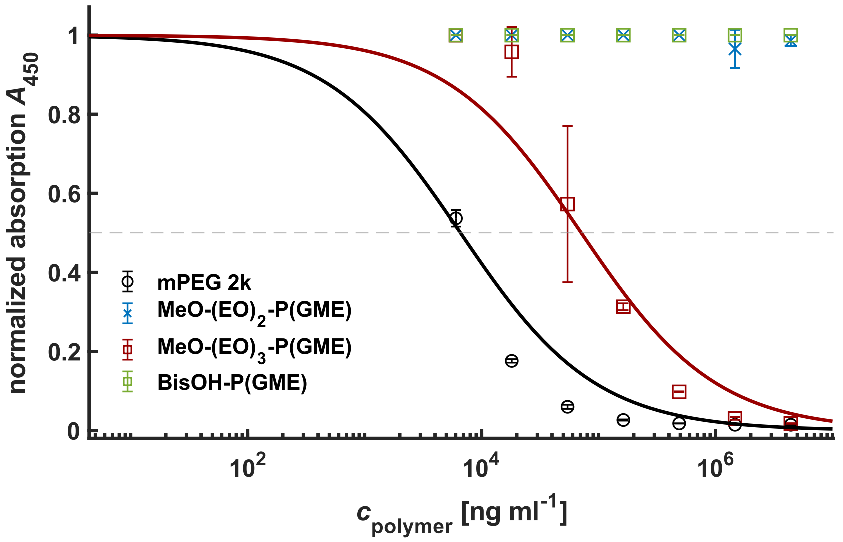

Figure S40. Influence of consecutive 2 (blue) or 3 (red) EO units at the initiator on the interactions of PGME with end group-selective APA.

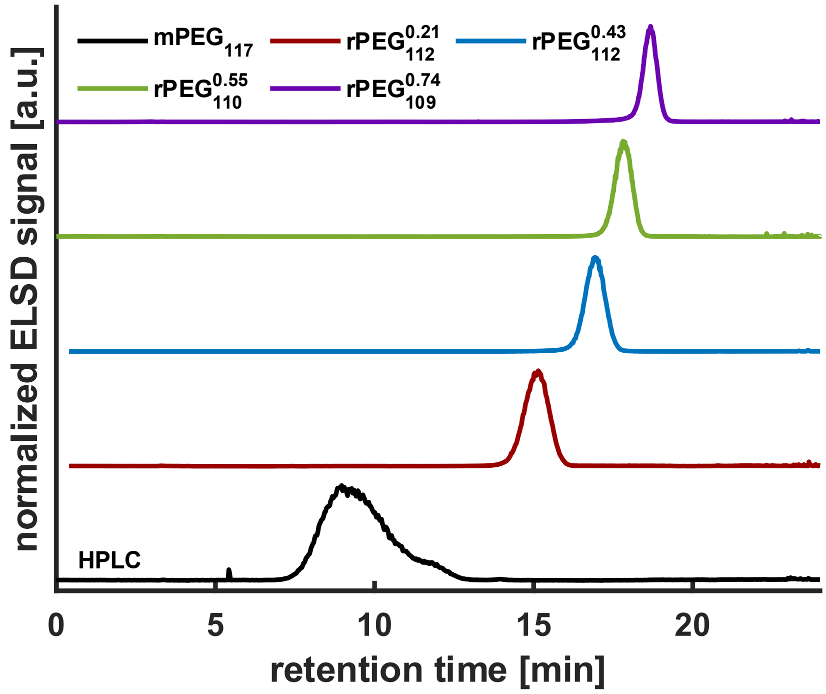

Figure S41. Stacked HPLC elugrams of investigated $mPEG_{117}$ and rPEG samples.

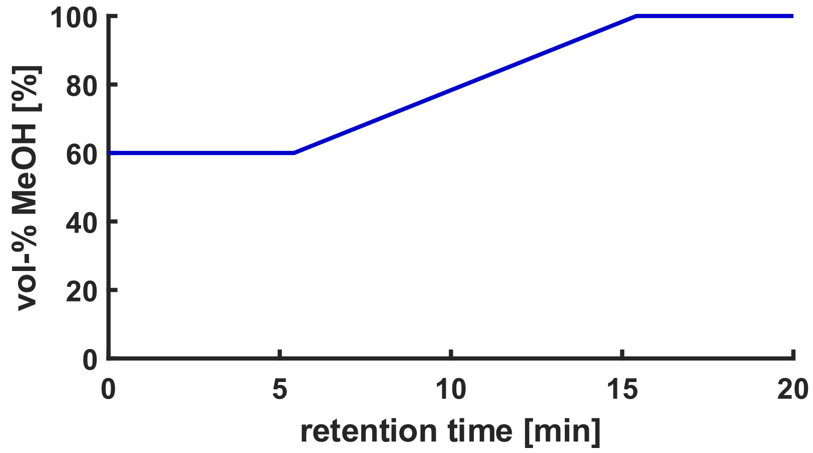

Figure S42. Applied solvent gradient conditions for mPEG and rPEG samples via analytical HPLC.

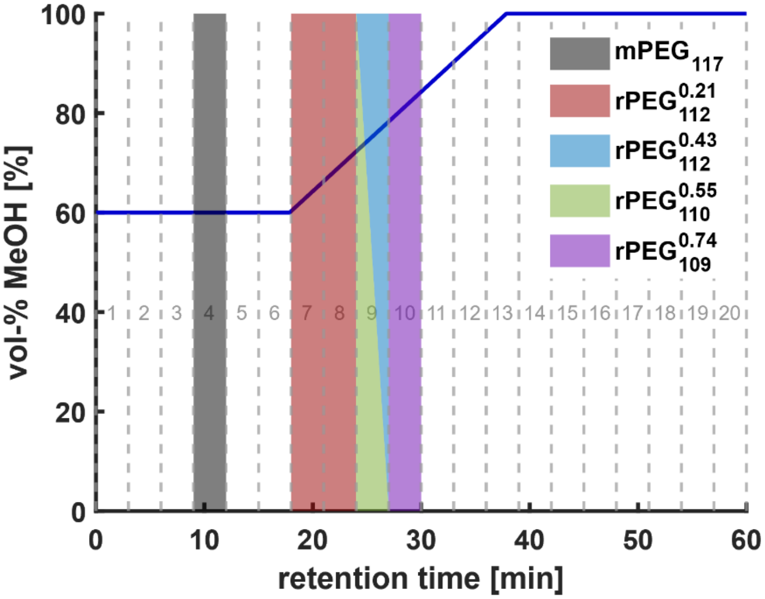

Figure S43. Applied solvent gradient conditions for mPEG and rPEG samples purification via semi-preparative HPLC (blue graph); product fraction(s) of each sample are highlighted.

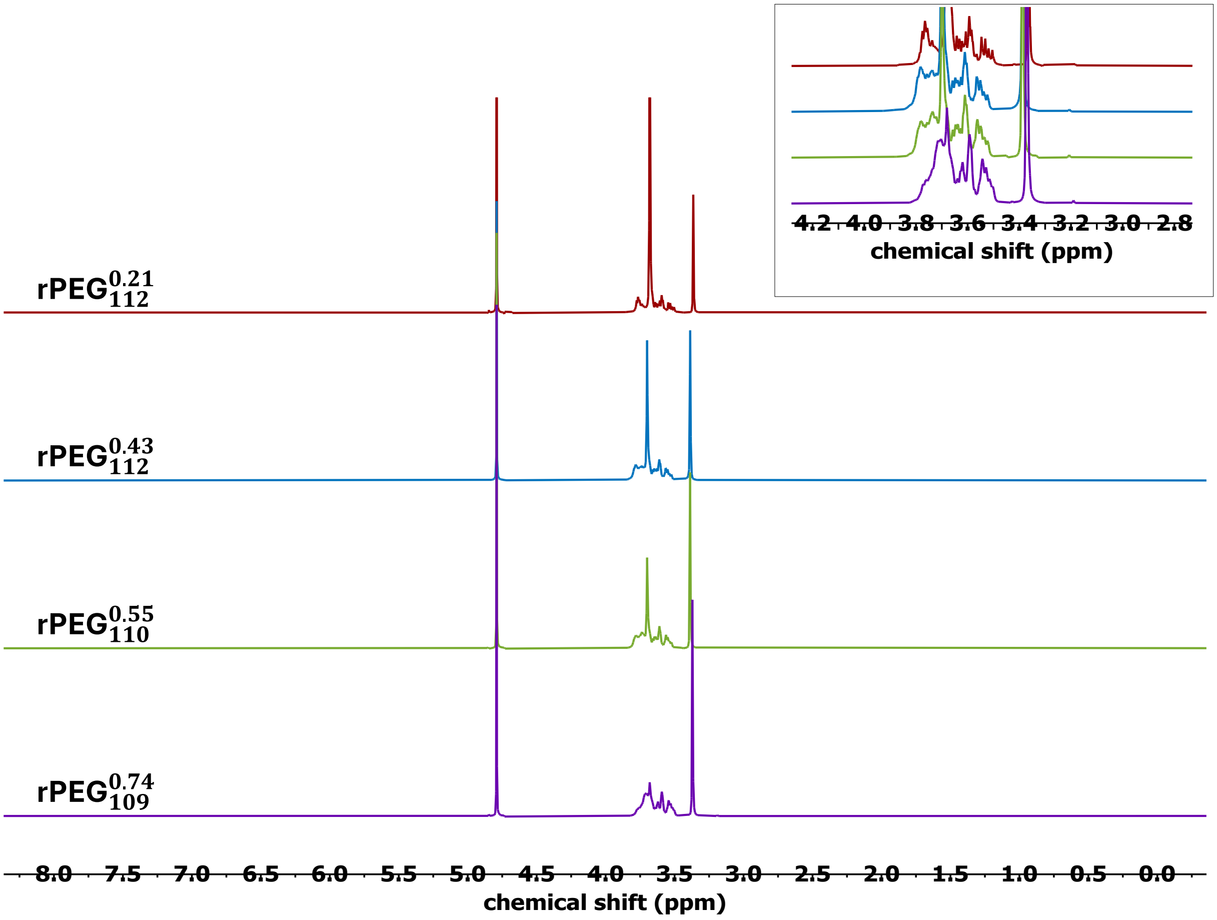

Figure S44. Stacked ^{1}H NMR spectra (D_2O, 400 MHz) of investigated rPEG samples.

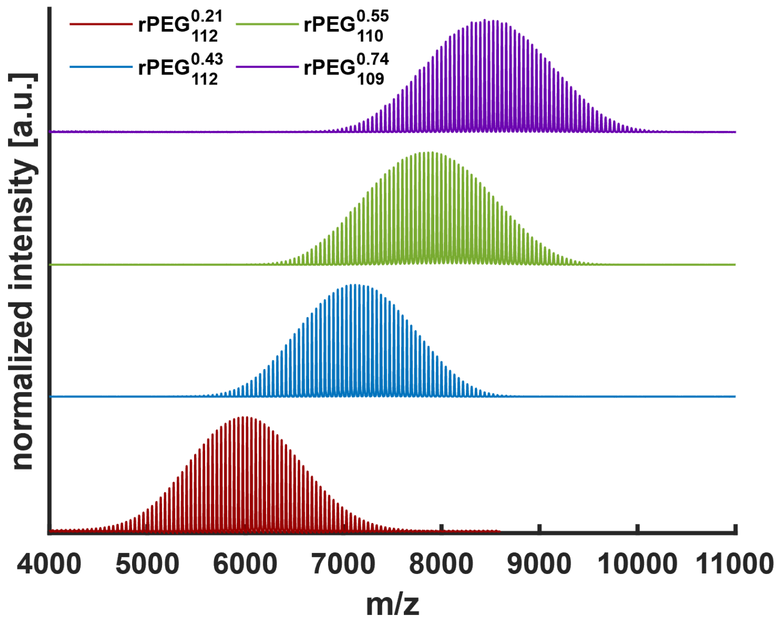

Figure S45. Stacked MALDI ToF mass spectra of synthesized rPEGs before HPLC purification.

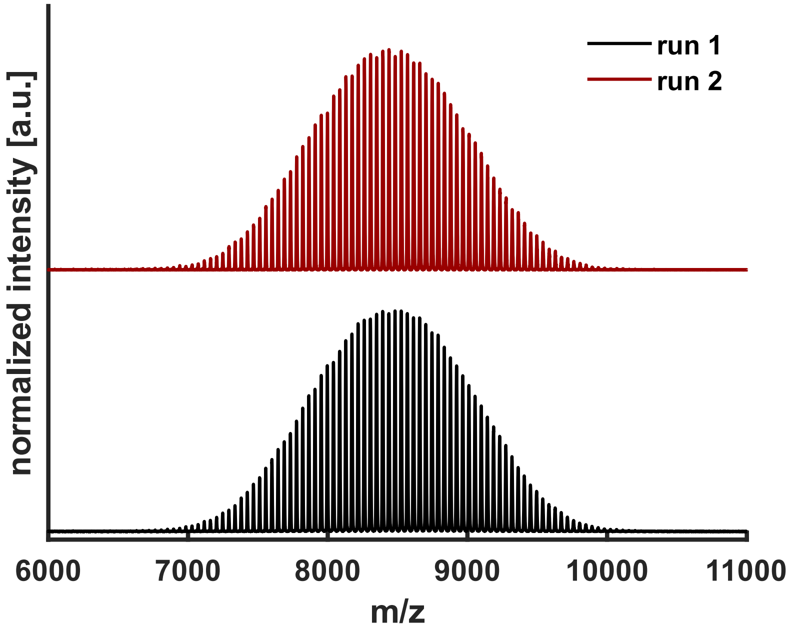

Figure S46. Stacked MALDI ToF mass spectra of purified rPEG$^{0.74}_{109}$ after different runs.

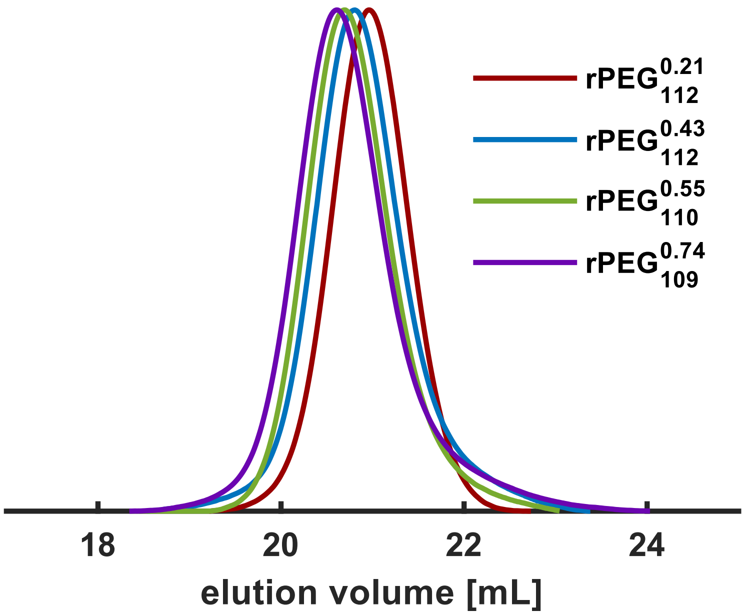

Figure S47. Stacked GPC elugrams of rPEG samples before HPLC purification.

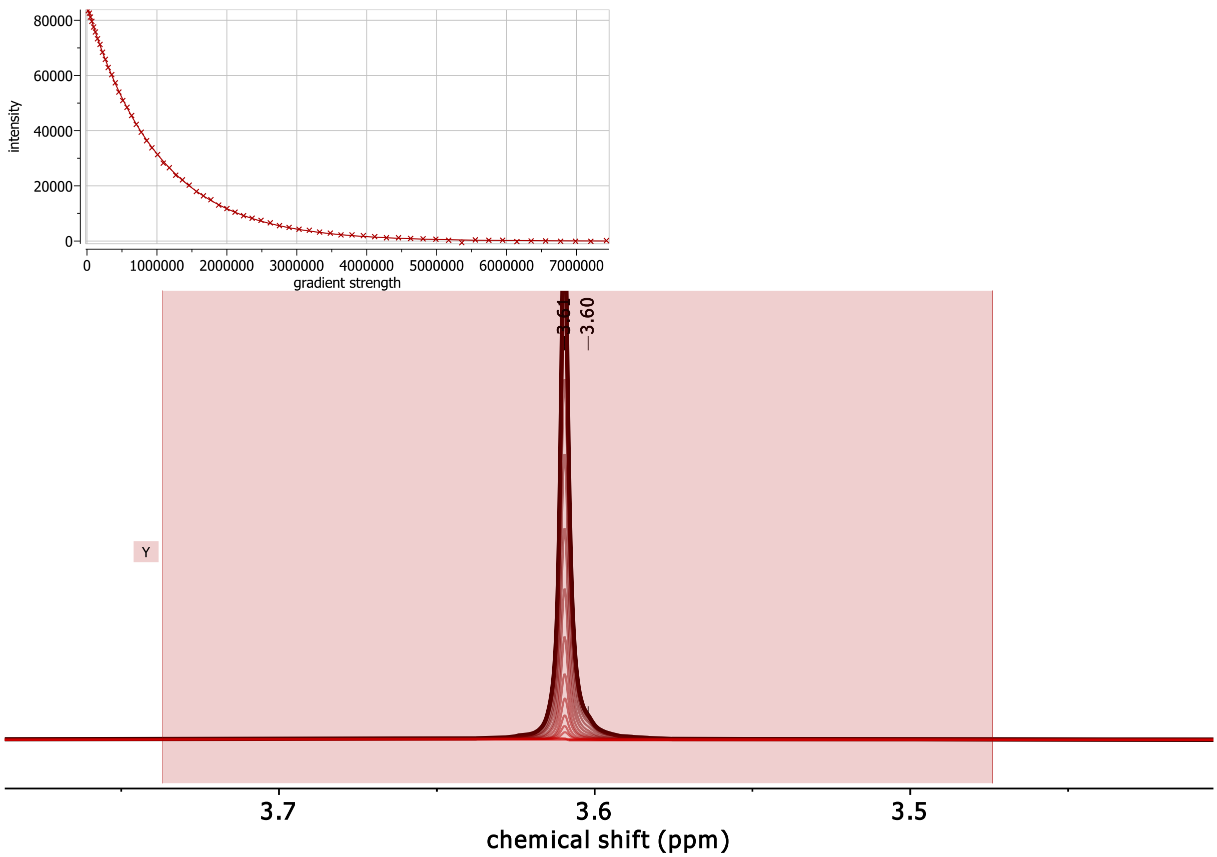

Figure S48. DOSY NMR spectrum (400 MHz, D_2O) of $mPEG_{117}$.

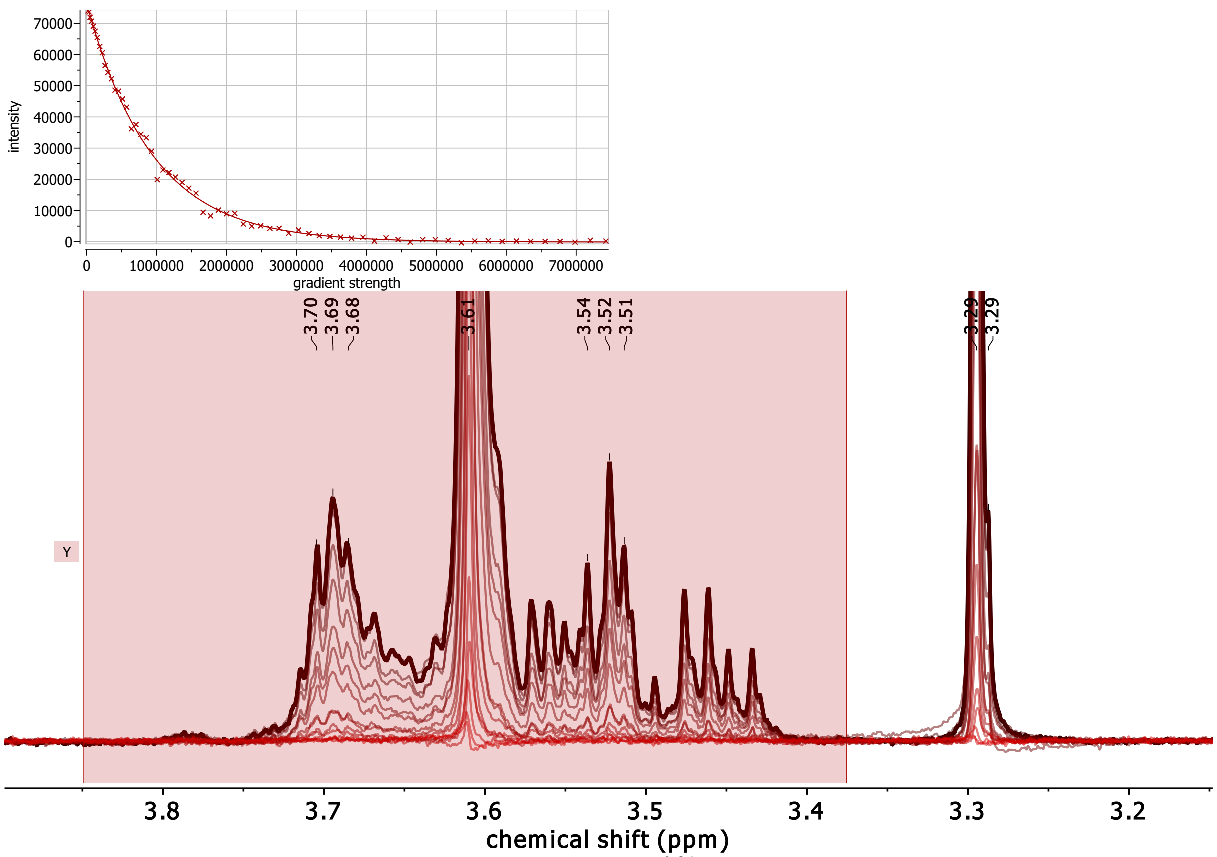

Figure S49. DOSY NMR spectrum (400 MHz, D_2O) of rPEG$^{0.21}_{112}$.

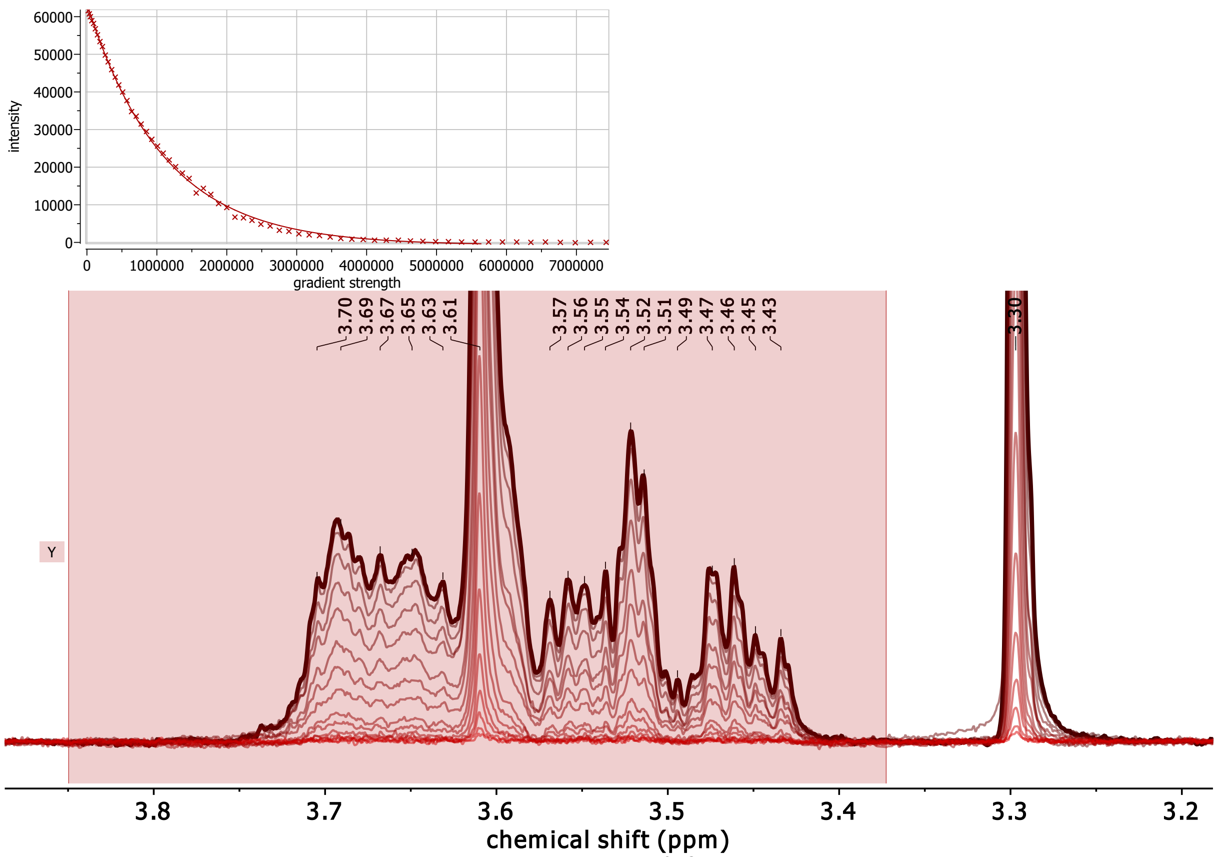

Figure S50. DOSY NMR spectrum (400 MHz, D_2O) of $rPEG_{112}^{0.43}$.

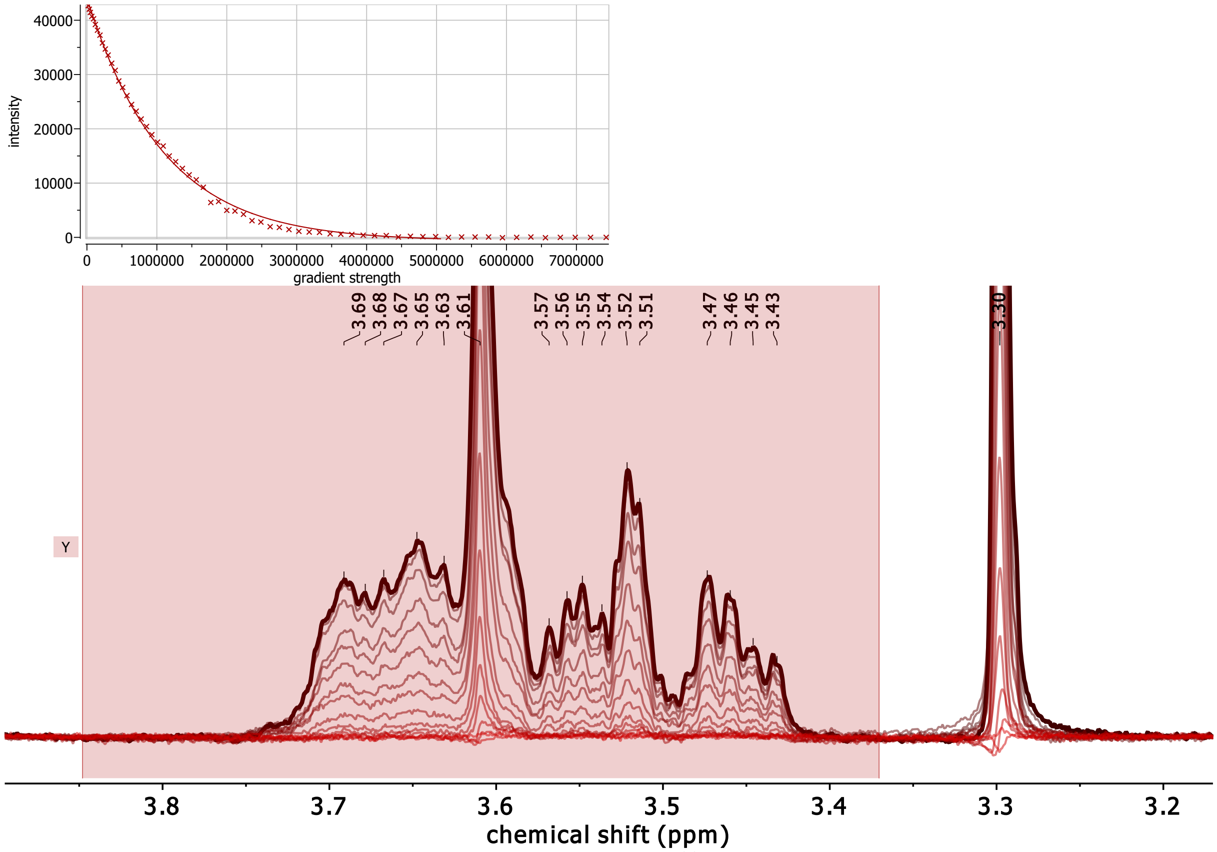

Figure S51. DOSY NMR spectrum (400 MHz, D_2O) of rPEG$^{0.55}_{110}$.

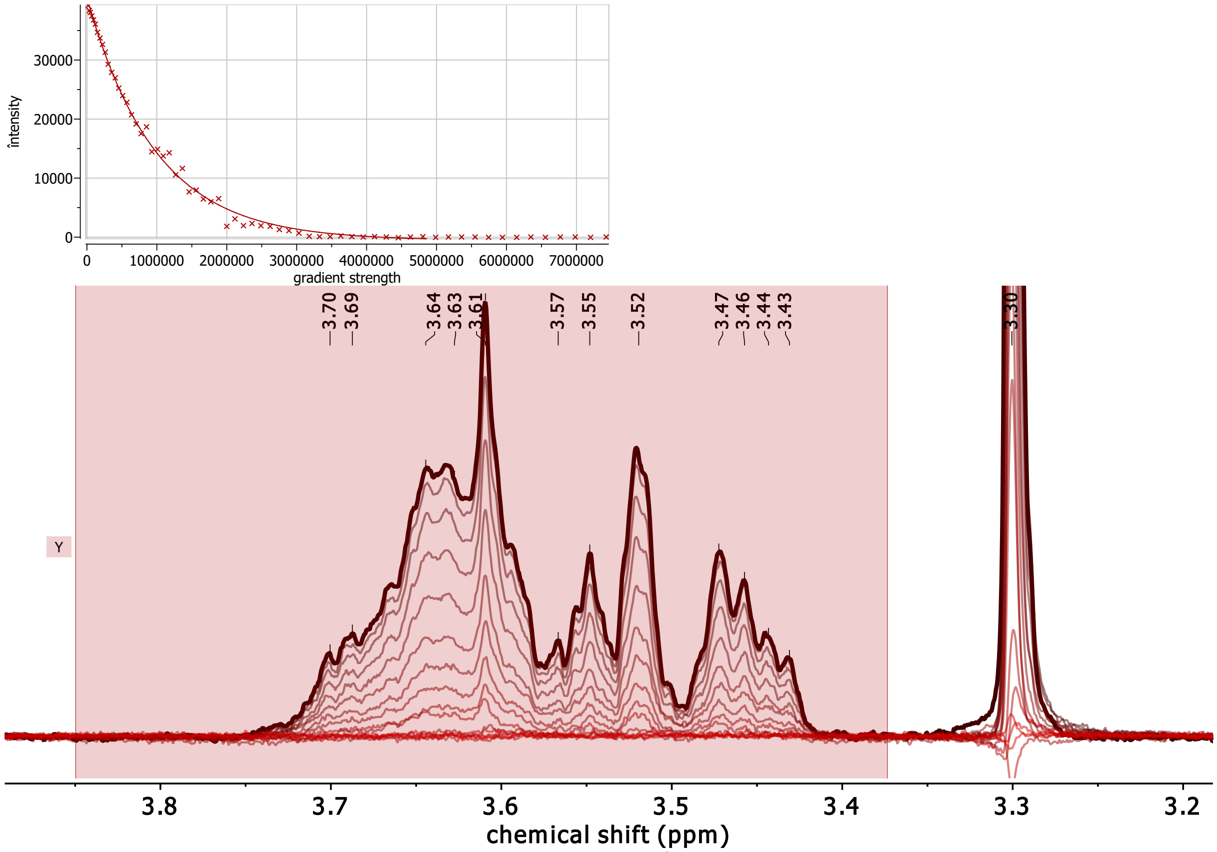

Figure S52. DOSY NMR spectrum (400 MHz, D_2O) of rPEG$^{0.74}_{109}$.

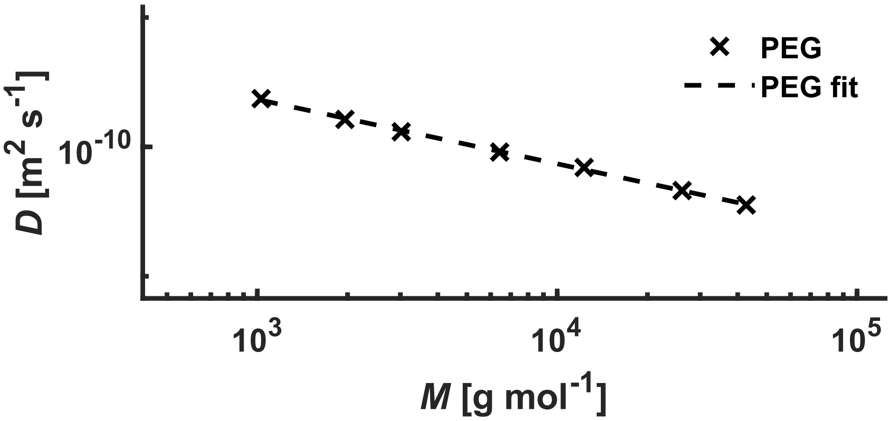

Figure S53. Diffusion coefficient (*D*) of PEG samples with different molar masses from *PSS* and resulting fit.

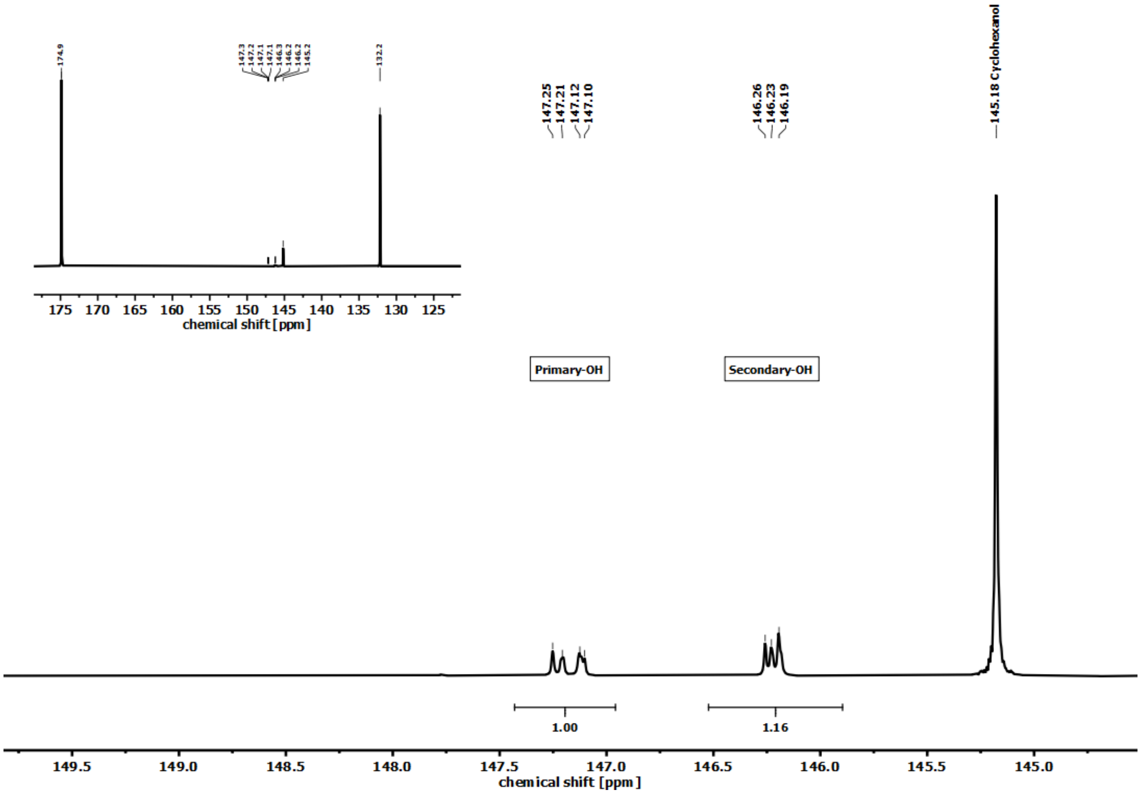

Figure S54: ^{31}P-IG NMR spectrum (162 MHz, $CDCl_3$/Pyridine-H_5) of rPEG$^{0.55}_{110}$ after reaction with 2-chloro-4,4,5,5-tetramethyldioxaphospholane.

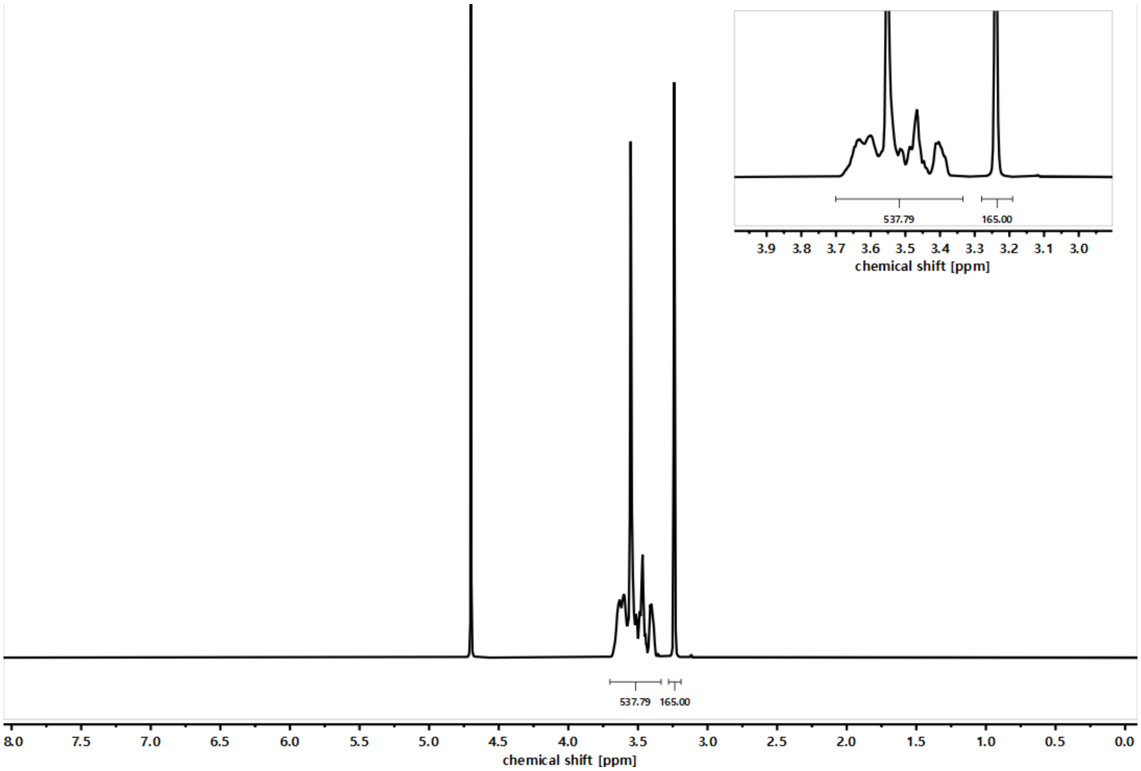

Figure S55: ^{1}H NMR spectrum (D_2O, 400 MHz) of rPEG$^{0.45}_{120}$.

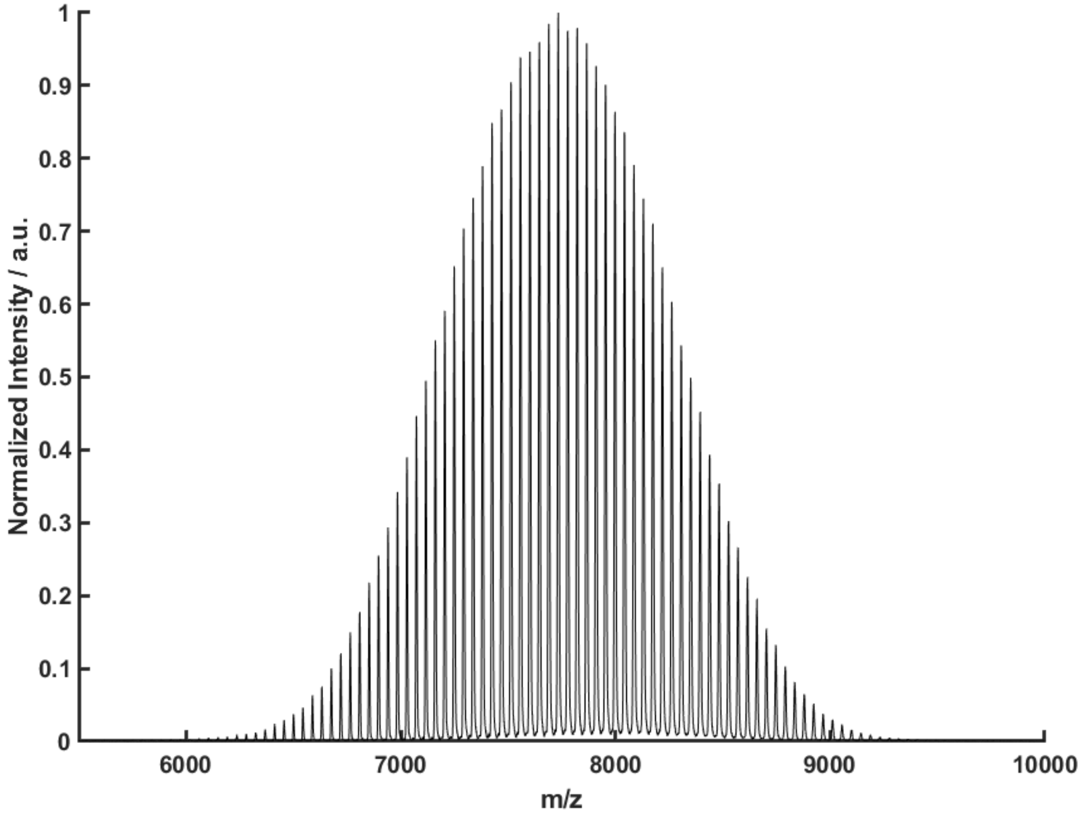

Figure S56 : MALDI ToF mass spectrum of purified $rPEG_{120}^{0.45}$.

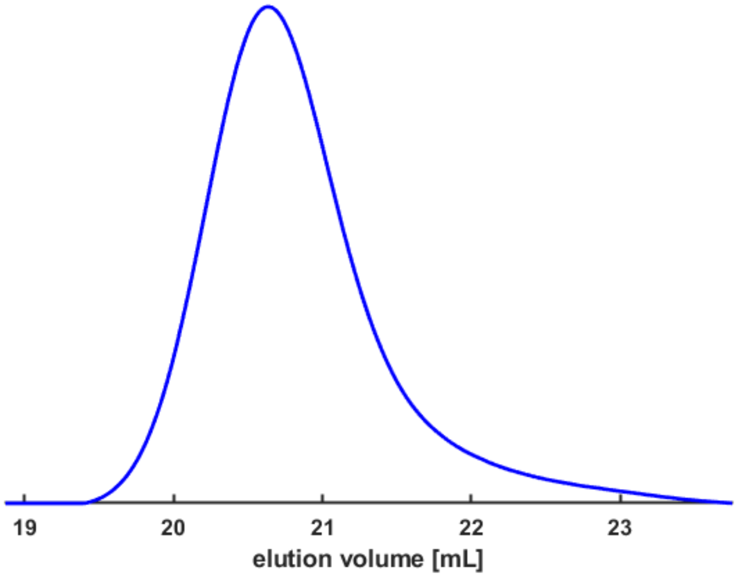

Figure S57: GPC elugram of $rPEG_{120}^{0.45}$ before HPLC purification. $Đ$ = 1.08.

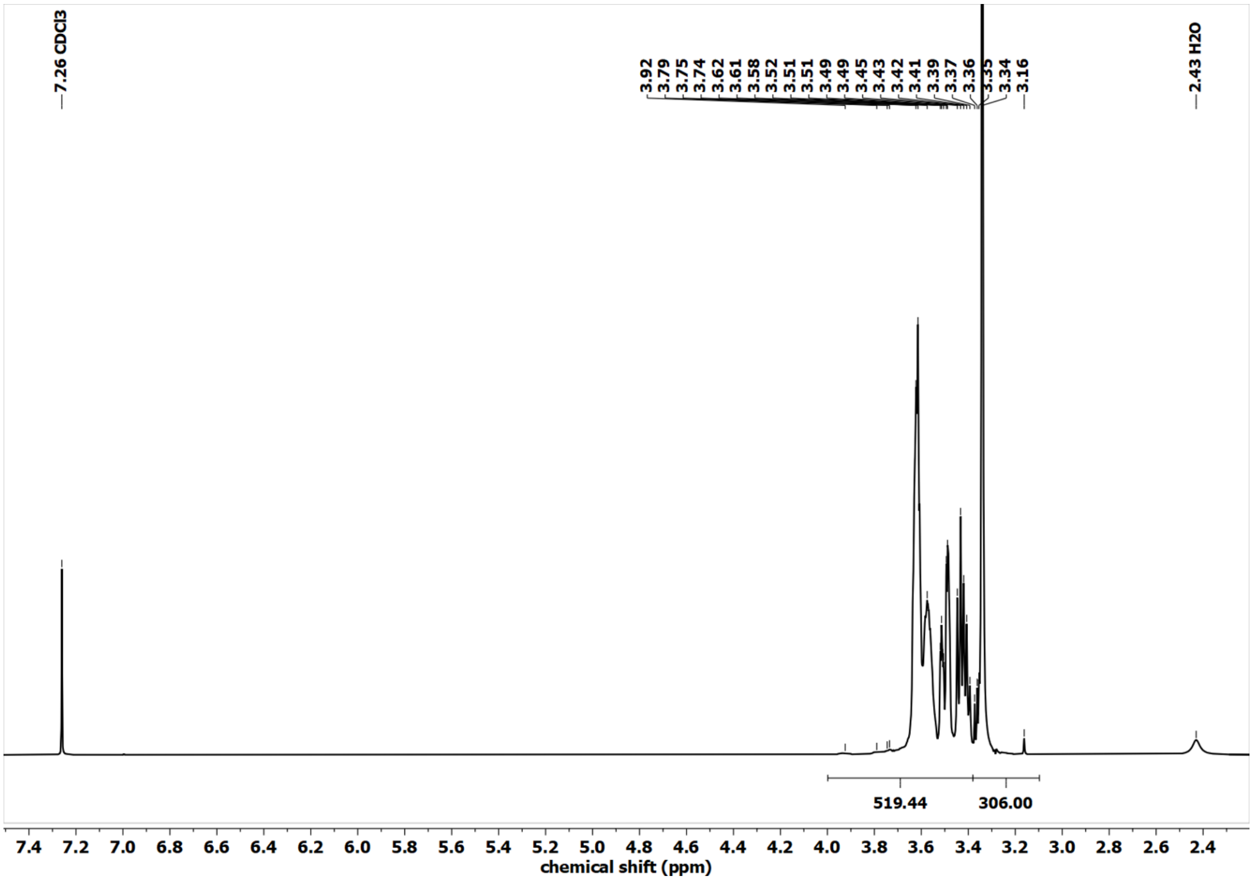

Figure S58: ^{1}H NMR spectrum ($CDCl_3$, 400 MHz) of $PGME_{103}$.

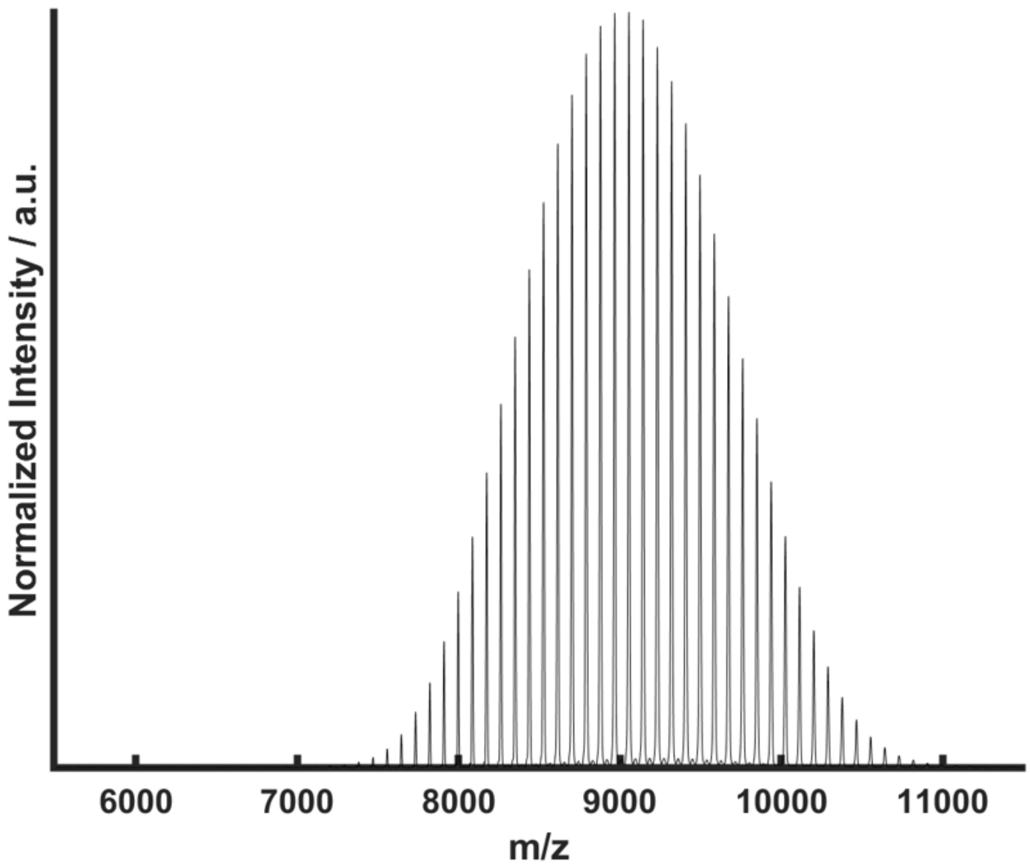

Figure S59: MALDI ToF mass spectrum of $PGME_{103}$ after preparative GPC purification.

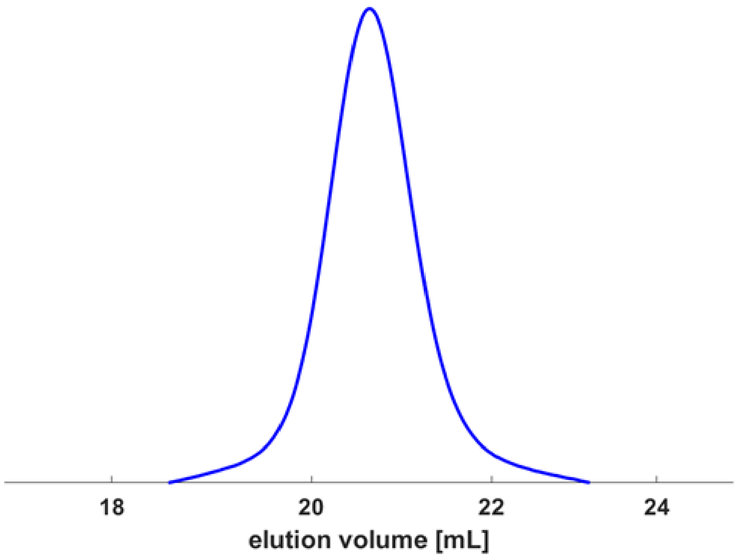

Figure S60: GPC elugram of $PGME_{103}$ after preparative GPC purification. $Đ$ = 1.05.

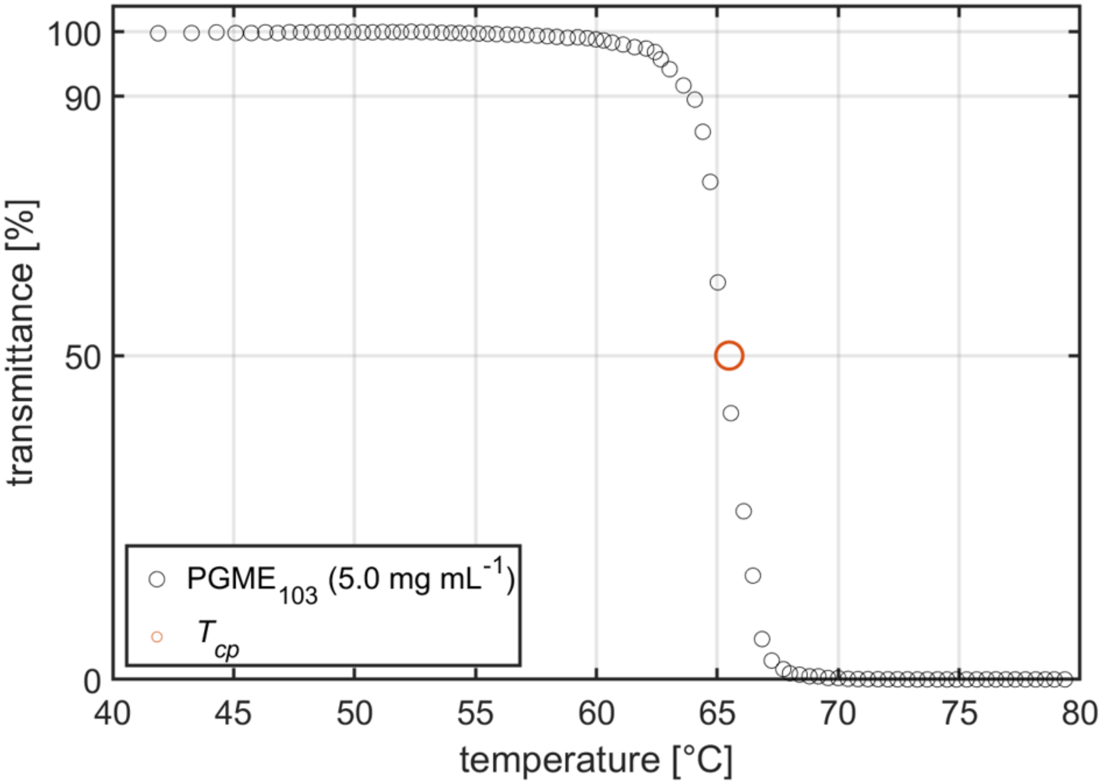

Figure S61: Turbidimetry plot of $PGME_{103}$ (heating curve) (5.0 mg mL^{-1}) in PBS buffer.

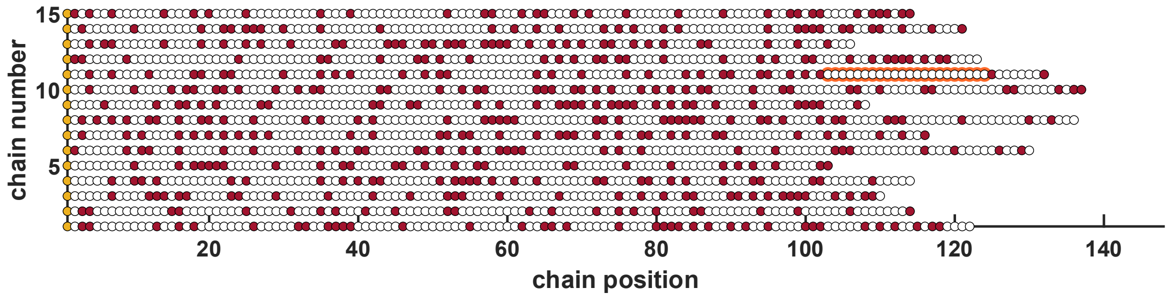

Figure S62. Section of 15 chains from the simulation (10^4 chains) of EO (white) and GME (red) repeating unit distribution at different chain positions of $rPEG_{114}^{0.30}$; repeating patterns with 16 consecutive EO repeating units are highlighted in orange; yellow circles represent the initiator.

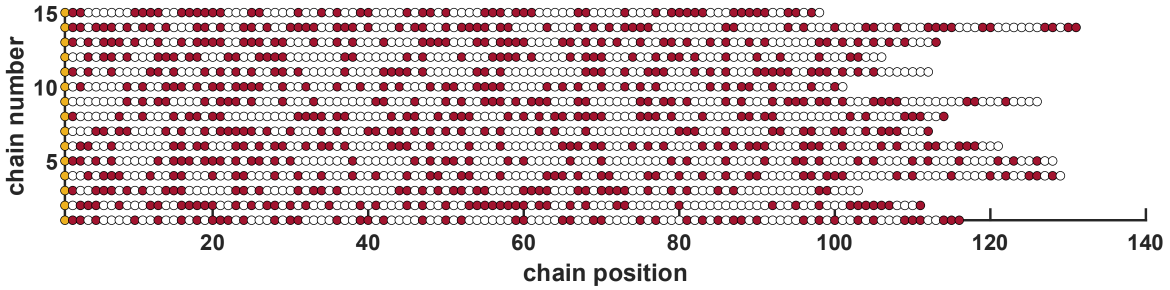

Figure S63. Section of 15 chains from the simulation (10^4 chains) of EO (white) and GME (red) repeating unit distribution at different chain positions of $rPEG_{114}^{0.40}$; yellow circles represent the initiator.

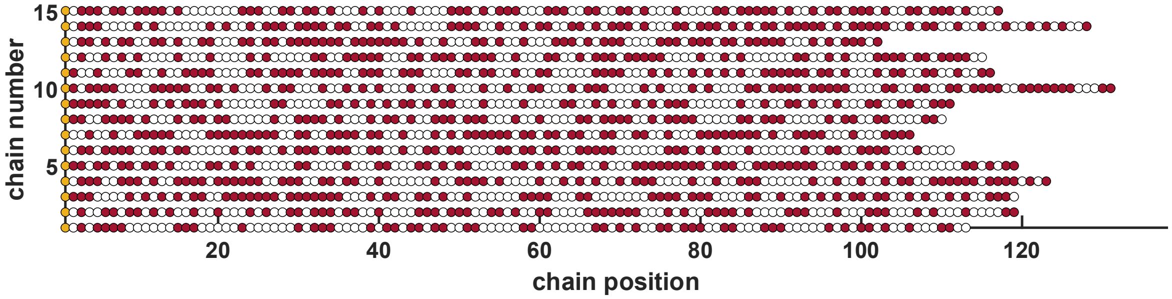

Figure S64. Section of 15 chains from the simulation (10^4 chains) of EO (white) and GME (red) repeating unit distribution at different chain positions of $rPEG_{114}^{0.50}$; yellow circles represent the initiator.

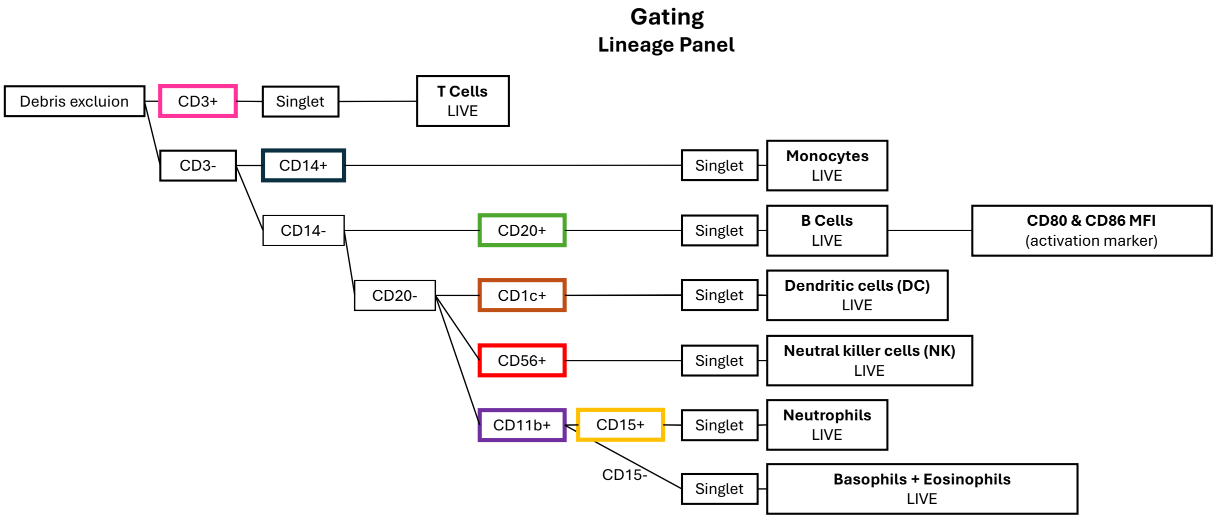

Figure S65. Schematic gating strategy applied to the flow cytometry evaluation. First cell debris was excluded, then the cell types were distinguished based on differential expression of lineage markers. Cell duplets were excluded and the viability of each singlet cell population was determined with Sytox™ Green™.

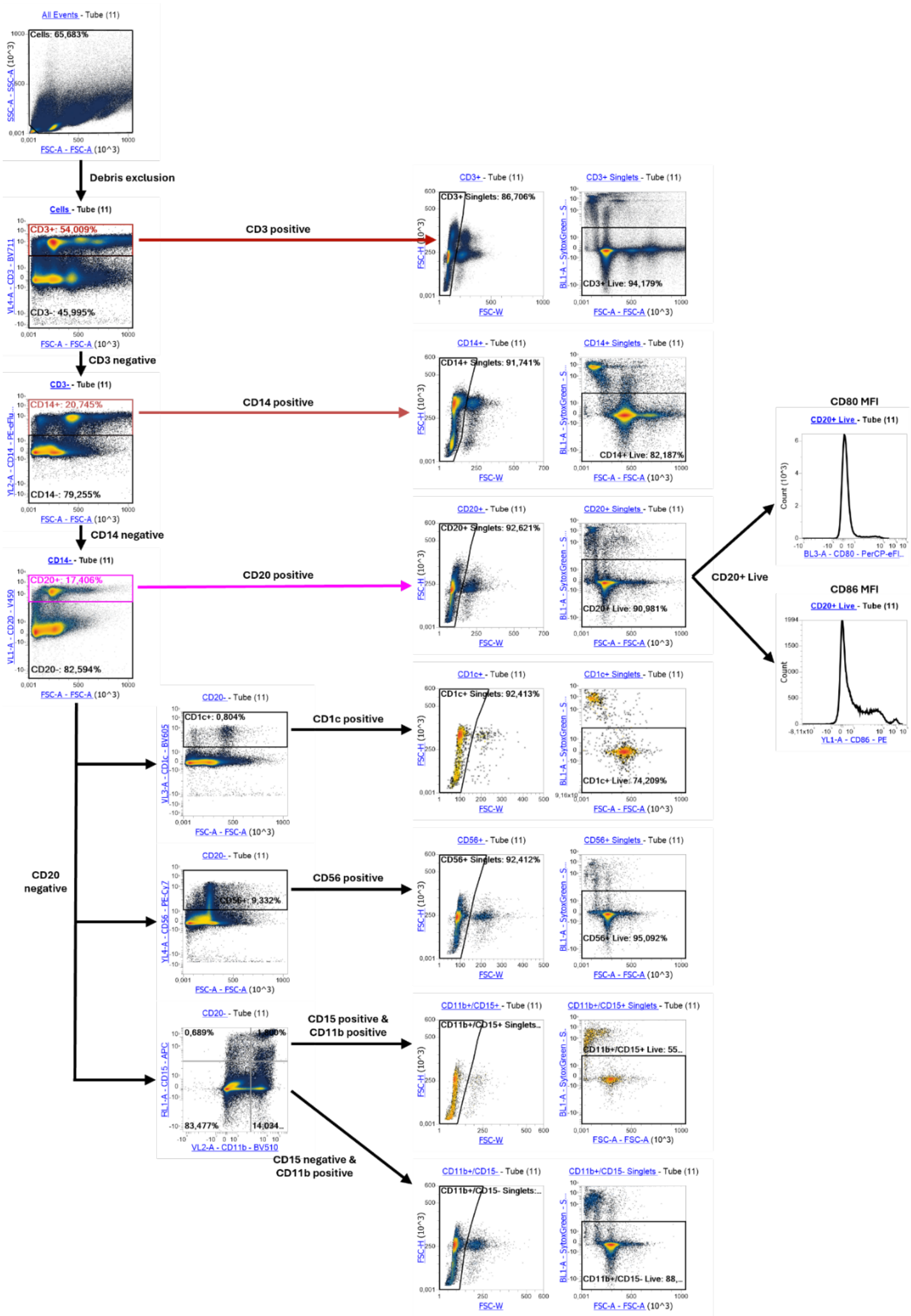

Figure S66. Exemplary gating strategy applied to the untreated control of donor 6 for flow cytometry evaluation. First cell debris were excluded, then the cell types were distinguished by detecting cell type-specific surface markers. Cell duplets were excluded and the viability of each singlet cell population was measured with Sytox™ Green™. For viable B cells (CD20⁺ Live) the MFI of the activation markers CD80 and CD86 were determined.

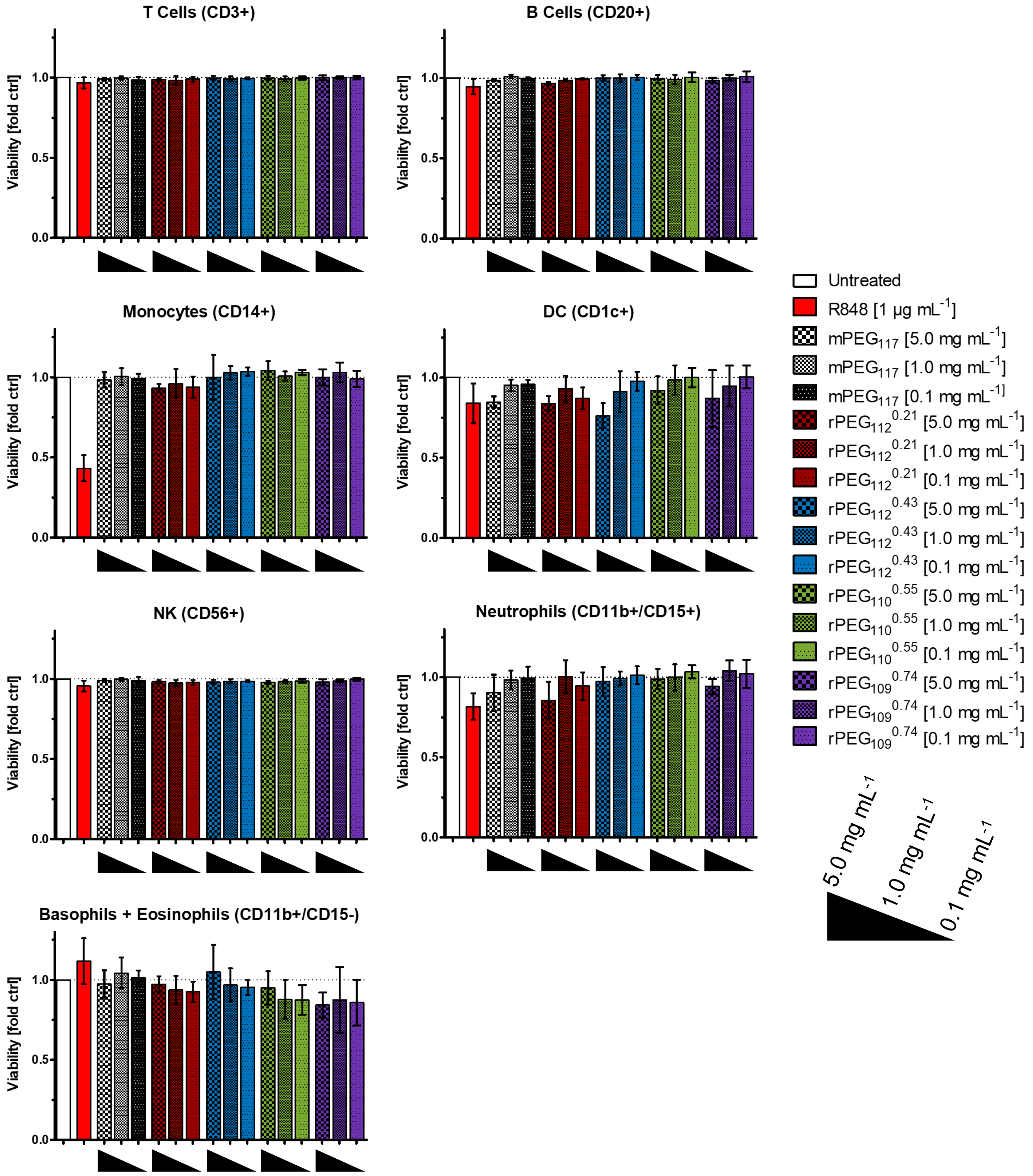

Figure S67. Normalized viability of human T cells, B cells, monocytes, dendritic cells (DC), neutral killer cells (NK), neutrophils and the remaining Basophils + Eosinophils. Determined with flow cytometry. The PBMC were isolated with density gradient centrifugation from whole blood of healthy donors ($N = 4$). Afterward, the PBMCs were incubated with the respective mPEG & rPEG samples for 16 hours.

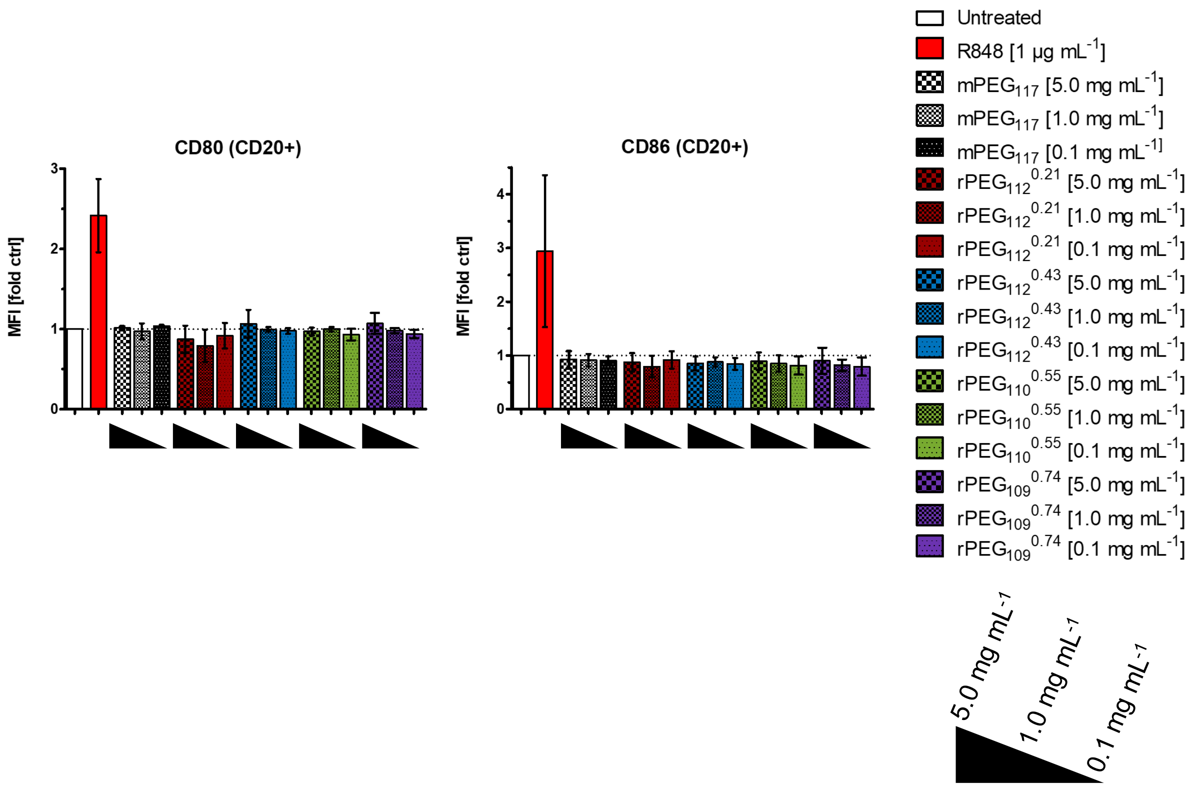

Figure S68. Analysis of immunomodulatory effects. Normalized mean fluorescence intensity (MFI) of CD80 and CD86 for viable CD20 positive cells (B cells). The PBMC were isolated with density gradient centrifugation from whole blood of healthy donors (*N* = 4). Afterward, the PBMCs were incubated with the respective formulations for 16 hours. As positive control, R848 was added to a final concentration of 1 µg mL^{-1} before incubation.

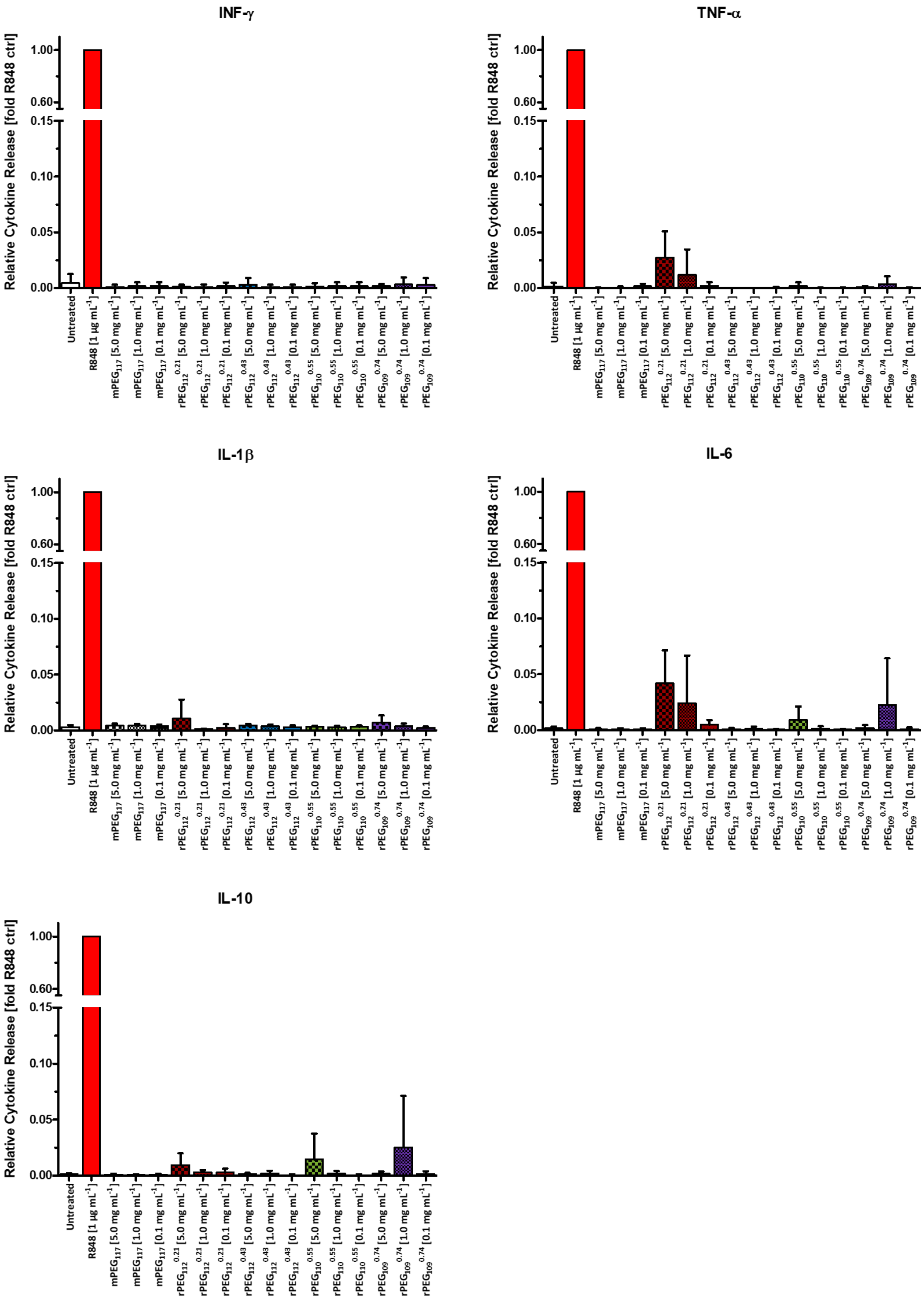

Figure S69. Relative cytokine release after 16 hours of incubation. Cytokine levels were normalized to each donor's positive control (R848). All polymer samples were measured with N = 4 whereas the untreated and the R848 control were measured with N = 8.

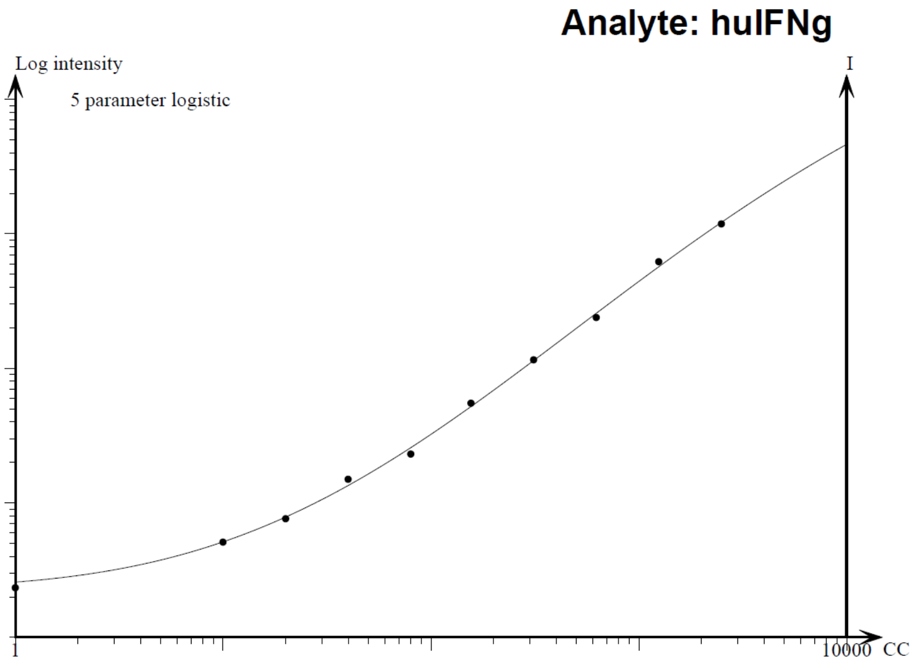

Figure S70. IFN-γ standard calibration curve.

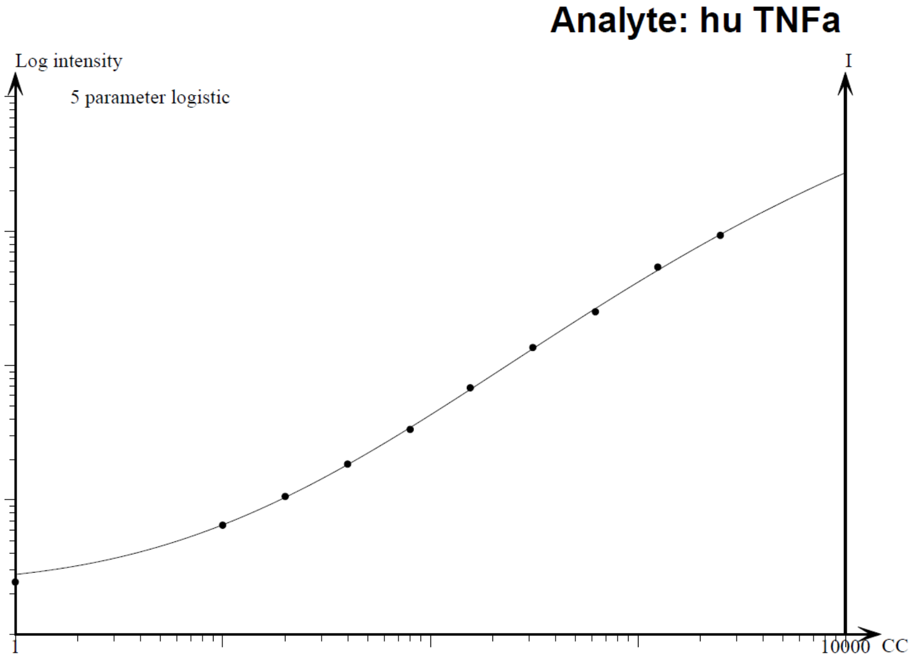

Figure S71. TNF-α standard calibration curve.

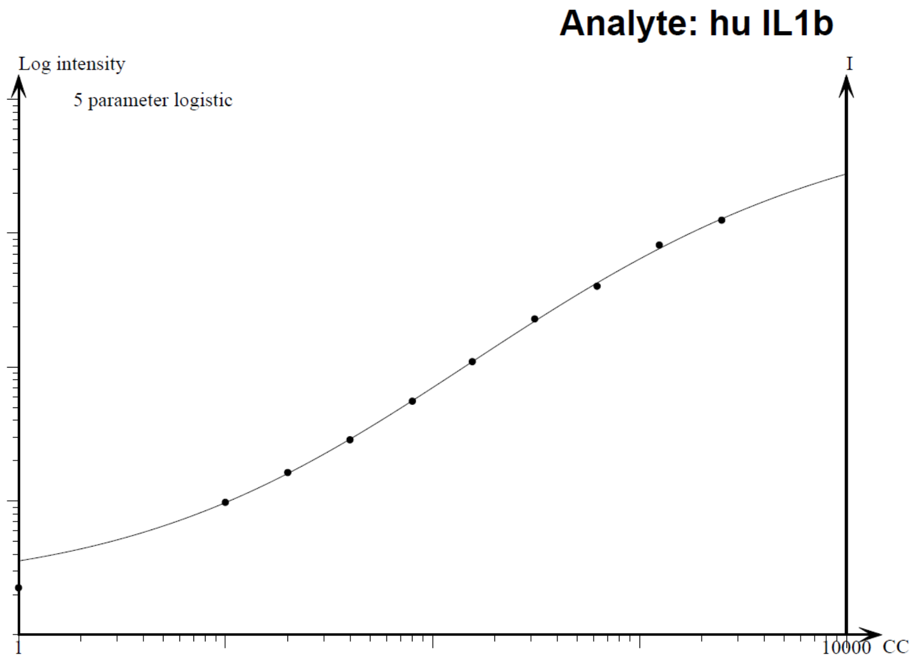

Figure S72. IL-1β standard calibration curve.

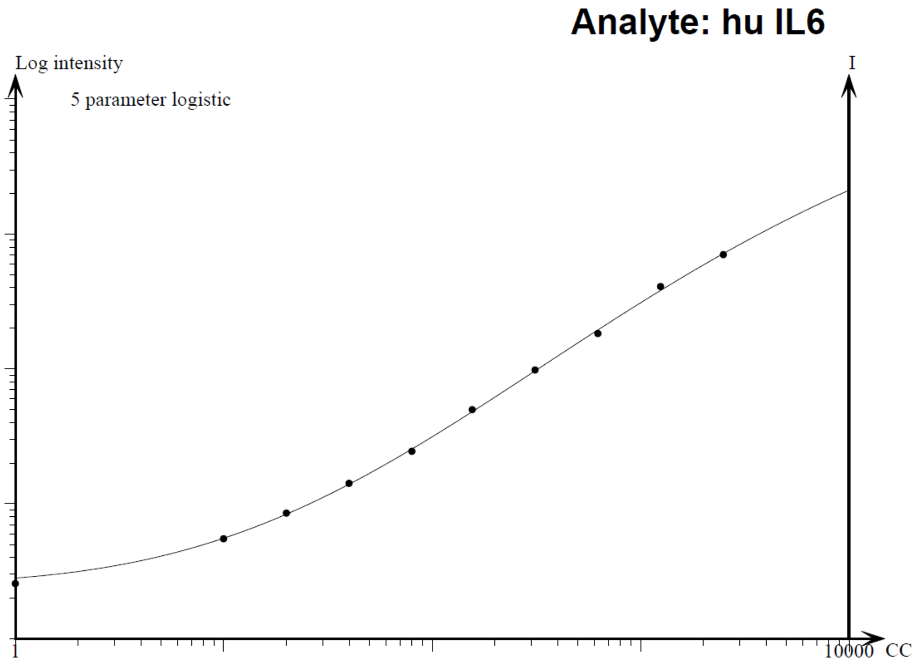

Figure S73. IL-6 standard calibration curve.

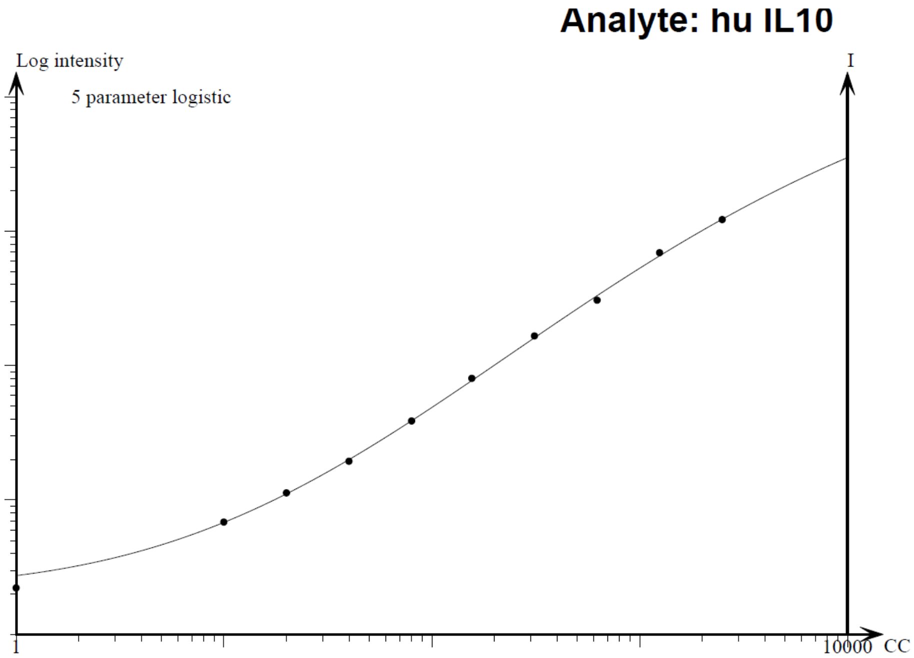

Figure S74. IL-10 standard calibration curve.

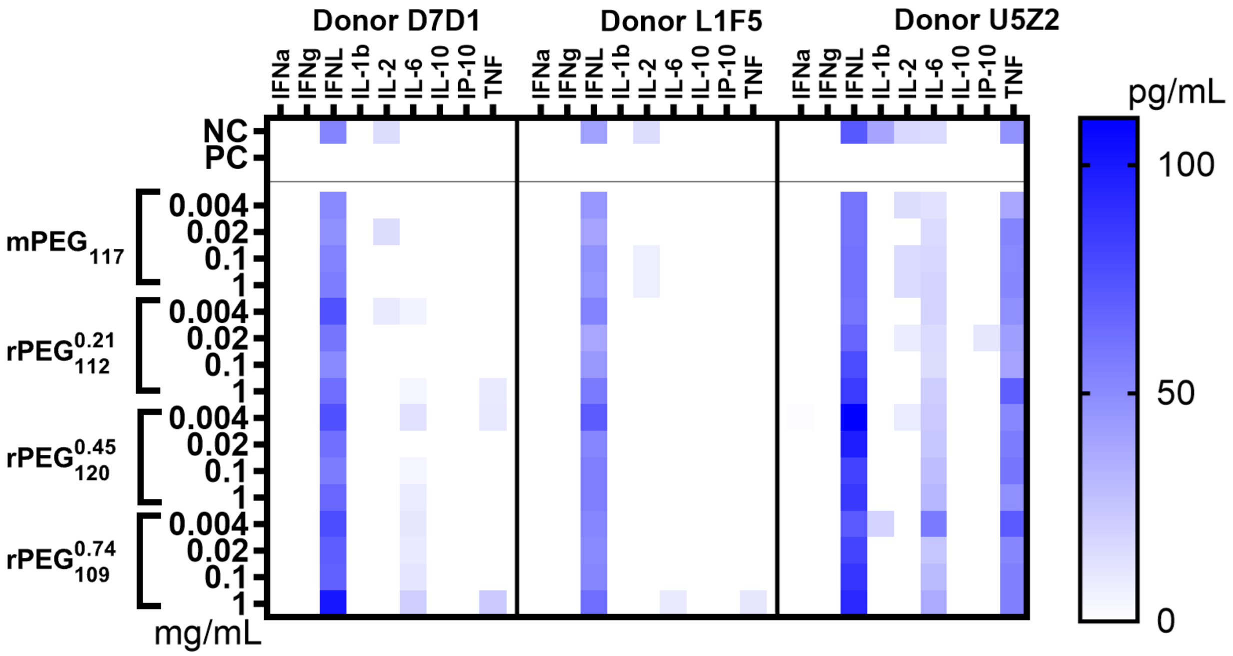

Figure S75: Cytokine biomarkers for activated myeloid and lymphoid cells in PBMC cultures exposed to $mPEG_{117}$, $rPEG^{0.21}_{112}$, $rPEG^{0.45}_{120}$; and $rPEG^{0.74}_{109}$. PBS, and a combination of PHA-M, LPS and ODN2216 were used as negative control (NC) and positive control (PC), respectively. Show herein is the same data as in Figure 4 but with the positive control data masked to bring up insignificant low levels of cytokines.

6. Tables S1 to S14

Table S1. Apparent rate constants of EO in the copolymerization with GME and potassium 2-(benzyloxy)ethoxide as initiator. Fit was applied for the linear regime in the pseudo-first-order plots.

Entry	Solvent	T [°C]	dod*	[I]† / 10^{-2} [mol L^{-1}]	$k_{app,copo}$ (EO) [s^{-1}]	R^2
1	DMSO	25	50%	3.80	1.17E-04§±0.01	>0.99
2	DMSO	25	90%	3.80	1.28E-04§±0.01	>0.99
3	Anisole	55	50%	9.66	2.99E-05±0.01	>0.99
4	Anisole	55	90%	9.66	8.10E-05±0.01	>0.99
5	Toluene	55	50%	9.66	1.46E-05±0.01	>0.99
6	Toluene	55	90%	9.66	1.43E-04±0.01	>0.99
7‡	Toluene	55	90%	9.66	2.68E-04±0.01	>0.99
8	DMSO	25	50%	3.60¶	4.78E-05±0.01	>0.99

*) Degree of deprotonation; †) theoretical chain end concentration; ‡) addition of 2 eq. of [18]crown-6 per potassium cation, §) multiplied by a factor of (9.66/3.80) to be comparable to the [I] of the anisole and toluene experiments; ¶) 3-ethoxy-1,2-propanediol was used as initiator.

Table S2. Copolymerization kinetics of GME and EO with potassium 2-(benzyloxy)ethoxide as initiator.

Entry	Solvent	T [°C]	dod*	[I]† / 10^{-2} [mol L^{-1}]	r_{GME}	r_{EO}	R^2	$Đ$§
1	DMSO	25	50%	3.80	1.03±0.01	0.97±0.01	>0.99	1.06
2	DMSO	25	90%	3.80	1.02±0.01	0.98±0.01	>0.99	1.07
3	Anisole	55	50%	9.66	1.37±0.01	0.73±0.01	>0.99	1.09
4	Anisole	55	90%	9.66	1.43±0.01	0.70±0.01	>0.99	1.08
5	Toluene	55	50%	9.66	1.52±0.01	0.66±0.01	>0.99	1.10
6	Toluene	55	90%	9.66	1.60±0.01	0.63±0.01	>0.99	1.10
7‡	Toluene	55	90%	9.66	1.55±0.01	0.64±0.01	>0.99	1.10
8	DMSO	25	50%	3.60¶	1.01±0.01	0.99±0.01	>0.99	1.10

*) Degree of deprotonation; †) theoretical chain end concentration; ‡) addition of 2 eq. of [18]crown-6; §) determined by GPC (eluent: DMF, PEG calibration, RI detector); ¶) active chain ends of 3-ethoxy-1,2-propanediol.

Table S3. Overview of characterizations of synthesized rPEG samples before HPLC purification.

Sample	$DP_{calc.}$	$DP_{MALDI+NMR}$	GME_{calc} [%]	GME_{NMR} [%]	$M_{n,calc.}$ [kg mol^{-1}]	$M_{n,MALDI}$ [kg mol^{-1}]	$M_{n,GPC}$ [kg mol^{-1}]	$Đ_{GPC}$
$\mathrm{rPEG}_{112}^{0.21}$	114	112	20	21	6.0	6.0	5.6	1.04
$\mathrm{rPEG}_{112}^{0.43}$	114	113	40	43	7.2	7.1	5.7	1.08
$\mathrm{rPEG}_{120}^{0.45}$	114	120	50	45	7.7	7.7	5.8	1.08
$\mathrm{rPEG}_{110}^{0.55}$	114	114	50	57	7.7	7.9	6.0	1.06
$\mathrm{rPEG}_{109}^{0.74}$	114	108	80	78	9.2	8.5	6.0	1.10

Table S4. Overview of characterizations of mPEG and rPEG samples after HPLC purification.

Sample	$DP_{calc.}$	$DP_{MALDI+NMR}$	GME_{calc} [%]	GME_{NMR} [%]	$M_{n,calc.}$ [kg mol^{-1}]	$M_{n,MALDI}$ [kg mol^{-1}]	$M_{n,GPC}$ [kg mol^{-1}]	$Đ_{GPC}$
mPEG_{117}	114	117	0	0	5.0	5.2	4.9	1.04
$\mathrm{rPEG}_{112}^{0.21}$	114	112	20	21	6.0	6.0	5.2	1.06
$\mathrm{rPEG}_{112}^{0.43}$	114	112	40	43	7.2	7.1	5.6	1.05
$\mathrm{rPEG}_{120}^{0.45}$	114	120	50	45	7.7	7.7	5.8	1.08
$\mathrm{rPEG}_{110}^{0.55}$	114	110	50	55	7.7	7.5	5.9	1.04
$\mathrm{rPEG}_{109}^{0.74}$	114	109	80	74	9.2	8.4	6.2	1.07

Table S5. Overview of HPLC data for investigated rPEGs.

Sample	$t_{e,max}$ [min]	$purity_{ELSD}$ [%a]
mPEG_{117}	8.9	>99
$\mathrm{rPEG}_{112}^{0.21}$	15.1	>99
$\mathrm{rPEG}_{112}^{0.43}$	16.9	>99
$\mathrm{rPEG}_{110}^{0.55}$	17.6	>99
$\mathrm{rPEG}_{109}^{0.74}$	18.7	>99

Table S6. Turbidimetric measurements of the cloud point temperatures (T_{cp}) of mPEG, investigated rPEG and PGME samples with varying molar GME contents in PBS buffer.

Sample	concentration [mg mL^{-1}]	T_{cp} at 90 % transmittance [°C]	T_{cp} at 50 % transmittance [°C]	T_{cp} at 0 % transmittance [°C]
mPEG$_{117}$	2.0	-*	-*	-*
rPEG$^{0.21}_{112}$	2.0	-*	-*	-*
rPEG$^{0.43}_{112}$	2.0	-*	-*	-*
rPEG$^{0.55}_{110}$	2.0	85.9	86.3	89.0
rPEG$^{0.74}_{109}$	2.0	74.1	74.6	78.4
rPEG$^{0.74}_{109}$	0.5	77.0	77.7	84.8
rPEG$^{0.74}_{109}$	1.0	75.8	76.3	81.4
rPEG$^{0.74}_{109}$	5.0	71.7	72.1	74.9
rPEG$^{0.74}_{109}$	10.0	70.8	71.2	73.7
PGME$_{103}$	5.0	64.0	65.5	70.8

*) no decrease in transmittance observed.

Table S7. Diffusion coefficient (D) and calculated hydrodynamic radii (r_H) of mPEG and investigated rPEG samples.

Sample	D [m^2 s^{-1}]	r_H [nm]
mPEG$_{117}$	9.92 10^{-11}	1.8
rPEG$^{0.21}_{112}$	1.07 10^{-10}	1.7
rPEG$^{0.43}_{112}$	9.10 10^{-11}	1.9
rPEG$^{0.55}_{110}$	9.21 10^{-11}	1.9
rPEG$^{0.74}_{109}$	1.02 10^{-10}	1.7

Table S8. Analysis concerning the occurrence of at least one hektakaidekads (16 EO units) within the 10^4 simulated rPEG chains with varying GME ratios (20 to 50 mol%).

Polymer	mol% GME [%]	probability of hektakaidekads [%]
rPEG$^{0.20}_{114}$	20	46.0
rPEG$^{0.30}_{114}$	30	10.1
rPEG$^{0.40}_{114}$	40	0.99
rPEG$^{0.50}_{114}$	50	0.09

Table S9. ELISA results for mPEG and rPEG samples.

Sample	$EC_{50,backbone}$ [ng ml^{-1}]	relative $affinity_{backbone}$	$EC_{50,end\ group}$ [ng ml^{-1}]	relative $affinity_{end\ group}$
$mPEG_{117}$	$3.31\cdot10^{1}$	1	$6.95\cdot10^{4}$	1
$rPEG_{112}^{0.21}$	$8.00\cdot10^{3}$	$4.14\cdot10^{-3}$	$4.35\cdot10^{6}$	$1.60\cdot10^{-2}$
$rPEG_{112}^{0.43}$	$1.28\cdot10^{5}$	$2.58\cdot10^{-4}$	$1.54\cdot10^{7}$	$4.50\cdot10^{-3}$
$rPEG_{110}^{0.55}$	$1.04\cdot10^{6}$	$3.18\cdot10^{-5}$	$3.64\cdot10^{7}$	$1.91\cdot10^{-3}$
$rPEG_{109}^{0.74}$	$7.10\cdot10^{6}$	$4.66\cdot10^{-6}$	$1.10\cdot10^{8}$	$6.23\cdot10^{-4}$

Table S10. Interferon-gamma (INF-γ) concentrations determined by CBA analysis.

Donor	INF-γ [pg mL^{-1}]							
	1	2	3	4	5	6	7	8
Untreated	0	1	0	36	0	3	4	0
R848 [1 µg mL^{-1}]	1374	1931	1744	1678	1255	1621	277	1481
$mPEG_{117}$ [5.0 µg mL^{-1}]	0	0	0	7	-†	-†	-†	-†
$mPEG_{117}$ [1.0 µg mL^{-1}]	0	0	0	12	-†	-†	-†	-†
$mPEG_{117}$ [0.1 µg mL^{-1}]	0	0	0	12	-†	-†	-†	-†
$rPEG_{112}^{0.21}$ [5.0 µg mL^{-1}]	-*	-*	-*	-*	0	1	1	1
$rPEG_{112}^{0.21}$ [1.0 µg mL^{-1}]	-*	-*	-*	-*	0	1	2	0
$rPEG_{112}^{0.21}$ [0.1 µg mL^{-1}]	-*	-*	-*	-*	0	2	2	0
$rPEG_{112}^{0.43}$ [5.0 µg mL^{-1}]	0	0	0	20	-†	-†	-†	-†
$rPEG_{112}^{0.43}$ [1.0 µg mL^{-1}]	0	0	0	7	-†	-†	-†	-†
$rPEG_{112}^{0.43}$ [0.1 µg mL^{-1}]	0	0	0	7	-†	-†	-†	-†
$rPEG_{110}^{0.55}$ [5.0 µg mL^{-1}]	0	0	0	10	-†	-†	-†	-†
$rPEG_{110}^{0.55}$ [1.0 µg mL^{-1}]	0	0	0	11	-†	-†	-†	-†
$rPEG_{110}^{0.55}$ [0.1 µg mL^{-1}]	0	1	0	11	-†	-†	-†	-†
$rPEG_{109}^{0.74}$ [5.0 µg mL^{-1}]	0	8	0	5	-†	-†	-†	-†
$rPEG_{109}^{0.74}$ [1.0 µg mL^{-1}]	0	0	0	21	-†	-†	-†	-†
$rPEG_{109}^{0.74}$ [0.1 µg mL^{-1}]	0	0	0	20	-†	-†	-†	-†

*) Sample was contaminated during the cell culture and discarded.

†) A second assay was deemed unnecessary due to success of the initial test.

Table S11. Tumor necrosis factor-alpha (TNF-α) concentrations determined by CBA analysis.

Donor	TNF-α [pg mL^{-1}]							
	1	2	3	4	5	6	7	8
Untreated	4	25	8	100	1	1	5	3
R848 [1 µg mL^{-1}]	12104	12937	10143	11766	6947	7763	7005	7602
mPEG$_{117}$ [5.0 µg mL^{-1}]	1	5	3	7	-†	-†	-†	-†
mPEG$_{117}$ [1.0 µg mL^{-1}]	2	5	2	19	-†	-†	-†	-†
mPEG$_{117}$ [0.1 µg mL^{-1}]	0	7	5	62	-†	-†	-†	-†
rPEG$_{112}^{0.21}$ [5.0 µg mL^{-1}]	-*	-*	-*	-*	48	114	195	454
rPEG$_{112}^{0.21}$ [1.0 µg mL^{-1}]	-*	-*	-*	-*	319	3	7	11
rPEG$_{112}^{0.21}$ [0.1 µg mL^{-1}]	-*	-*	-*	-*	1	2	4	52
rPEG$_{112}^{0.43}$ [5.0 µg mL^{-1}]	2	4	0	9068‡	-†	-†	-†	-†
rPEG$_{112}^{0.43}$ [1.0 µg mL^{-1}]	2	6	1	5	-†	-†	-†	-†
rPEG$_{112}^{0.43}$ [0.1 µg mL^{-1}]	0	9	0	12	-†	-†	-†	-†
rPEG$_{110}^{0.55}$ [5.0 µg mL^{-1}]	2	5	3	80	-†	-†	-†	-†
rPEG$_{110}^{0.55}$ [1.0 µg mL^{-1}]	2	7	1	5	-†	-†	-†	-†
rPEG$_{110}^{0.55}$ [0.1 µg mL^{-1}]	2	6	2	17	-†	-†	-†	-†
rPEG$_{109}^{0.74}$ [5.0 µg mL^{-1}]	0	1350‡	0	25	-†	-†	-†	-†
rPEG$_{109}^{0.74}$ [1.0 µg mL^{-1}]	1	3	0	162	-†	-†	-†	-†
rPEG$_{109}^{0.74}$ [0.1 µg mL^{-1}]	2	2	0	11	-†	-†	-†	-†

*) Sample was contaminated during the cell culture and discarded.

†) A second assay was deemed unnecessary due to success of the initial test.

‡) Excluded outliner.

Table S12. Interleukin-1 beta (IL-1β) concentrations determined by CBA analysis.

Donor	IL-1β [pg mL^{-1}]							
	1	2	3	4	5	6	7	8
Untreated	3	5	3	3	15	23	43	12
R848 [1 µg mL^{-1}]	1212	1411	466	778	14349	13632	11554	10659
$mPEG_{117}$ [5.0 µg mL^{-1}]	4	4	3	3	-†	-†	-†	-†
$mPEG_{117}$ [1.0 µg mL^{-1}]	4	4	3	4	-†	-†	-†	-†
$mPEG_{117}$ [0.1 µg mL^{-1}]	3	4	3	3	-†	-†	-†	-†
$rPEG^{0.21}_{112}$ [5.0 µg mL^{-1}]	-*	-*	-*	-*	25	20	48	383
$rPEG^{0.21}_{112}$ [1.0 µg mL^{-1}]	-*	-*	-*	-*	31	8	11	10
$rPEG^{0.21}_{112}$ [0.1 µg mL^{-1}]	-*	-*	-*	-*	5	9	18	72
$rPEG^{0.43}_{112}$ [5.0 µg mL^{-1}]	4	5	3	44‡	-†	-†	-†	-†
$rPEG^{0.43}_{112}$ [1.0 µg mL^{-1}]	4	4	3	2	-†	-†	-†	-†
$rPEG^{0.43}_{112}$ [0.1 µg mL^{-1}]	3	3	2	2	-†	-†	-†	-†
$rPEG^{0.55}_{110}$ [5.0 µg mL^{-1}]	4	4	2	3	-†	-†	-†	-†
$rPEG^{0.55}_{110}$ [1.0 µg mL^{-1}]	3	4	2	2	-†	-†	-†	-†
$rPEG^{0.55}_{110}$ [0.1 µg mL^{-1}]	3	3	2	2	-†	-†	-†	-†
$rPEG^{0.74}_{109}$ [5.0 µg mL^{-1}]	3	23	3	2	-†	-†	-†	-†
$rPEG^{0.74}_{109}$ [1.0 µg mL^{-1}]	3	3	2	5	-†	-†	-†	-†
$rPEG^{0.74}_{109}$ [0.1 µg mL^{-1}]	3	3	2	2	-†	-†	-†	-†

*) Sample was contaminated during the cell culture and discarded.

†) A second assay was deemed unnecessary due to success of the initial test.

‡) Excluded outliner.

Table S13. Interleukin-6 (IL-6) concentrations determined by CBA analysis.

Donor	IL-6 [pg mL^{-1}]							
	1	2	3	4	5	6	7	8
Untreated	11	70	20	242	58	112	117	787
R848 [1 µg mL^{-1}]	98783	113245	114487	103224	41024	38029	29742	298497
$mPEG_{117}$ [5.0 µg mL^{-1}]	21	124	65	195	-[†]	-[†]	-[†]	-[†]
$mPEG_{117}$ [1.0 µg mL^{-1}]	33	97	24	209	-[†]	-[†]	-[†]	-[†]
$mPEG_{117}$ [0.1 µg mL^{-1}]	17	88	24	202	-[†]	-[†]	-[†]	-[†]
$rPEG_{112}^{0.21}$ [5.0 µg mL^{-1}]	-[*]	-[*]	-[*]	-[*]	240	1159	2172	17191
$rPEG_{112}^{0.21}$ [1.0 µg mL^{-1}]	-[*]	-[*]	-[*]	-[*]	3597	111	125	699
$rPEG_{112}^{0.21}$ [0.1 µg mL^{-1}]	-[*]	-[*]	-[*]	-[*]	83	100	111	3288
$rPEG_{112}^{0.43}$ [5.0 µg mL^{-1}]	116	250	36	92779[‡]	-[†]	-[†]	-[†]	-[†]
$rPEG_{112}^{0.43}$ [1.0 µg mL^{-1}]	27	414	25	140	-[†]	-[†]	-[†]	-[†]
$rPEG_{112}^{0.43}$ [0.1 µg mL^{-1}]	16	104	19	132	-[†]	-[†]	-[†]	-[†]
$rPEG_{110}^{0.55}$ [5.0 µg mL^{-1}]	183	730	97	2773	-[†]	-[†]	-[†]	-[†]
$rPEG_{110}^{0.55}$ [1.0 µg mL^{-1}]	18	504	13	165	-[†]	-[†]	-[†]	-[†]
$rPEG_{110}^{0.55}$ [0.1 µg mL^{-1}]	14	79	16	139	-[†]	-[†]	-[†]	-[†]
$rPEG_{109}^{0.74}$ [5.0 µg mL^{-1}]	25	126369[‡]	68	541	-[†]	-[†]	-[†]	-[†]
$rPEG_{109}^{0.74}$ [1.0 µg mL^{-1}]	66	559	18	8815	-[†]	-[†]	-[†]	-[†]
$rPEG_{109}^{0.74}$ [0.1 µg mL^{-1}]	16	91	17	283	-[†]	-[†]	-[†]	-[†]

*) Sample was contaminated during the cell culture and discarded.

†) A second assay was deemed unnecessary due to success of the initial test.

‡) Excluded outliner.

Table S14. Interleukin-10 (IL-10) concentrations determined by CBA analysis.

Donor	IL-10 [pg mL^{-1}]							
	1	2	3	4	5	6	7	8
Untreated	2	4	4	9	3	1	1	1
R848 [1 µg mL^{-1}]	5539	4615	5291	3421	2094	930	593	358
$mPEG_{117}$ [5.0 µg mL^{-1}]	2	5	5	7	-†	-†	-†	-†
$mPEG_{117}$ [1.0 µg mL^{-1}]	3	6	2	5	-†	-†	-†	-†
$mPEG_{117}$ [0.1 µg mL^{-1}]	2	5	3	5	-†	-†	-†	-†
$rPEG_{112}^{0.21}$ [5.0 µg mL^{-1}]	-*	-*	-*	-*	7	5	3	9
$rPEG_{112}^{0.21}$ [1.0 µg mL^{-1}]	-*	-*	-*	-*	8	2	1	2
$rPEG_{112}^{0.21}$ [0.1 µg mL^{-1}]	-*	-*	-*	-*	3	1	1	3
$rPEG_{112}^{0.43}$ [5.0 µg mL^{-1}]	8	10	2	5352‡	-†	-†	-†	-†
$rPEG_{112}^{0.43}$ [1.0 µg mL^{-1}]	2	21	2	9	-†	-†	-†	-†
$rPEG_{112}^{0.43}$ [0.1 µg mL^{-1}]	2	6	2	4	-†	-†	-†	-†
$rPEG_{110}^{0.55}$ [5.0 µg mL^{-1}]	14	20	12	167	-†	-†	-†	-†
$rPEG_{110}^{0.55}$ [1.0 µg mL^{-1}]	2	24	2	8	-†	-†	-†	-†
$rPEG_{110}^{0.55}$ [0.1 µg mL^{-1}]	2	4	2	4	-†	-†	-†	-†
$rPEG_{109}^{0.74}$ [5.0 µg mL^{-1}]	2	2421‡	5	13	-†	-†	-†	-†
$rPEG_{109}^{0.74}$ [1.0 µg mL^{-1}]	10	18	3	320	-†	-†	-†	-†
$rPEG_{109}^{0.74}$ [0.1 µg mL^{-1}]	2	4	2	17	-†	-†	-†	-†

*) Sample was contaminated during the cell culture and discarded.

†) A second assay was deemed unnecessary due to success of the initial test.

‡) Excluded outliner.

References

(1) Steube, M.; Johann, T.; Plank, M.; Tjaberings, S.; Gröschel, A. H.; Gallei, M.; Frey, H.; Müller, A. H. E. Kinetics of Anionic Living Copolymerization of Isoprene and Styrene Using in Situ NIR Spectroscopy: Temperature Effects on Monomer Sequence and Morphology. *Macromolecules* **2019**, *52* (23), 9299–9310. DOI: 10.1021/acs.macromol.9b01790.

(2) Spyros, A. Quantitative determination of the distribution of free hydroxylic and carboxylic groups in unsaturated polyester and alkyd resins by 31 P ‐ NMR spectroscopy. *J. Appl. Polym. Sci.* **2002**, *83* (8), 1635–1642. DOI: 10.1002/app.10069.

(3) *National Cancer Institute's Nanotechnology Characterization Laboratory Assay Cascade Protocols,* National Cancer Institute (US), 2005.

(4) Cedrone, E.; Potter, T. M.; Neun, B. W.; Tyler, A.; Dobrovolskaia, M. A. *National Cancer Institute's Nanotechnology Characterization Laboratory Assay Cascade Protocols*:*Multiplex Enzyme-Linked Immunosorbent Assay (ELISA) for Detection of Human Cytokines in Culture Supernatants: Version 1*, 2005. DOI: 10.17917/P2T3-8P84.

(5) Potter, T. M.; Cedrone, E.; Neun, B. W.; Dobrovolskaia, M. A. *National Cancer Institute's Nanotechnology Characterization Laboratory Assay Cascade Protocols*:*Preparation of Human Whole Blood and Peripheral Blood Mononuclear Cell Cultures to Analyze Nanoparticles' Potential to Induce Cytokines In Vitro: Version 3.1*, 2005. DOI: 10.17917/C7G596.

(6) Neun, B. W.; Cedrone, E.; Dobrovolskaia, M. A. *National Cancer Institute's Nanotechnology Characterization Laboratory Assay Cascade Protocols*:*Analysis of Platelet Aggregation by Cell Counting: Version 4*, 2005. DOI: 10.17917/0165-RC67.

(7) Neun, B.; Rodriguez, J.; Ilinskaya, A.; Dobrovolskaia, M. *NCL Method ITA-12*.

(8) Sedlacek, O.; Janouskova, O.; Verbraeken, B.; Hoogenboom, R. Straightforward Route to Superhydrophilic Poly(2-oxazoline)s via Acylation of Well-Defined Polyethylenimine. *Biomacromolecules* **2019**, *20* (1), 222–230. DOI: 10.1021/acs.biomac.8b01366.

(9) Odian, G., Ed. *Principles of Polymerization, Fourth Edition*, 4th ed.; John Wiley & Sons Incorporated, 2004.

(10) Blankenburg, J.; Kersten, E.; Maciol, K.; Wagner, M.; Zarbakhsh, S.; Frey, H. The poly(propylene oxide- co -ethylene oxide) gradient is controlled by the polymerization method: determination of reactivity ratios by direct comparison of different copolymerization models. *Polym. Chem.* **2019**, *10* (22), 2863–2871. DOI: 10.1039/C9PY00500E.

(11) Beckingham, B. S.; Sanoja, G. E.; Lynd, N. A. Simple and Accurate Determination of Reactivity Ratios Using a Nonterminal Model of Chain Copolymerization. *Macromolecules* **2015**, *48* (19), 6922–6930. DOI: 10.1021/acs.macromol.5b01631.

(12) Jaacks, V. A novel method of determination of reactivity ratios in binary and ternary copolymerizations. *Makromol. Chem.* **1972**, *161* (1), 161–172. DOI: 10.1002/macp.1972.021610110.

(13) Solov'yanov, A. A.; Kazanskii, K. S. The kinetics and mechanism of anionic polymerization of ethylene oxide in ether solvents. *Polym. Sci.* **1972**, *14* (5), 1186–1195. DOI: 10.1016/0032-3950(72)90162-1.

(14) Duda, A.; Penczek, S. Determination of the Absolute Propagation Rate Constants in Polymerization with Reversible Aggregation of Active Centers. *Macromolecules* **1994**, *27* (18), 4867–4870. DOI: 10.1021/ma00096a002.
